# Mafu Yishen Formula ameliorates membranous nephropathy by promotion of regulatory T cell differentiation: a multi-omics study

**DOI:** 10.1186/s13020-025-01272-1

**Published:** 2026-01-06

**Authors:** Qihan Zhao, Haoran Dai, Naiqian Zhang, Shuxian Huang, Xiaoyu Cui, Yanyu Cui, Hanxue Jiang, Wu Liu, Yangzi Chen, Yalin Zheng, Qian Ding, Yuehong Hu, Gen Li, Xinyue Tang, Yang Zheng, Hongliang Rui, Baoli Liu

**Affiliations:** 1 Nephrology and Rheumatology Immunology Diagnosis and Treatment Center, Beijing Hospital of Traditional Chinese Medicine, Capital Medical University, Beijing, 100010 China; 2 Beijing University of Chinese Medicine, Beijing, 100105 China; 3 Laboratory for Clinical Medicine, Capital Medical University, Beijing, 100069 China; 4 School of Traditional Chinese Medicine, Capital Medical University, Beijing, 100069 China; 5 School of Pharmacy & State Key Laboratory of Quality Research in Chinese Medicine & Laboratory of Drug Discovery From Natural Resources and Industrialization, Macau University of Science and Technology, Taipa, Macau SAR China; 6 Beijing Institute of Chinese Medicine, Beijing, 100010 China

**Keywords:** Mafu Yishen Formula, Membranous nephropathy, Regulatory T cell, PI3K/AKT signaling pathway, Immune tolerance, Multi-omics

## Abstract

**Background:**

Primary membranous nephropathy (PMN) is one of the main causes of nephrotic syndrome in adults, with edema as the primary symptom. The Mafu Yishen Formula (MFYS) is derived from a combination of classical prescriptions for treating edema diseases recorded in the *Treatise on Cold Damage and Miscellaneous Diseases*. While clinical efficacy of MFYS in treating PMN has been supported by evidence-based studies, its specific mechanisms remain unclear. This study aims to investigate the therapeutic effects and molecular mechanisms of MFYS in the treatment of membranous nephropathy, with a focus on whether MFYS promotes regulatory T cells (Treg) differentiation and modulates immune responses.

**Methods:**

The passive Heymann nephritis (PHN) rat model was employed to simulate human PMN. Rats were treated with either low- or high-dose Mafu Yishen Formula (MFYS) or cyclosporine A (CsA) as a positive control. Urinary protein levels, serum biochemical parameters, renal pathological changes, and podocyte injury were evaluated. Immunofluorescence and flow cytometry were used to assess renal IgG deposition, B cell proliferation, and the proportion of Treg. Serum cytokine levels were measured using appropriate assays. The absorbed components of MFYS were identified via metabolomic analysis. Integrated strategies including network pharmacology, spleen transcriptomics, and proteomics were applied to identify key targets and signaling pathways. In vitro Treg polarization assays, supplemented with pathway inhibitors, were conducted to validate mechanistic findings. Molecular docking simulations were performed to explore interactions between active components of MFYS and potential target proteins.

**Results:**

MFYS significantly reduced urinary protein levels in PHN rats, improved biochemical indicators such as serum albumin and blood lipids. Pathological examination revealed that MFYS alleviated glomerular and podocyte injury, while also reducing intrarenal IgG deposition and suppressing splenic B cell activation. Serum tests indicated that MFYS increased levels of IL-2 and IL-10 while decreasing levels of IL-6 and IL-17. Notably, MFYS significantly increased the proportion of Tregs. Integrated network pharmacology and multi-omics analysis consistently revealed that MFYS upregulates the PI3K/AKT signaling pathway and enhances mitochondrial oxidative phosphorylation. Furthermore, MFYS intervention down regulated pSTAT3 expression while promoting PGC-1α expression. In vitro experiments further confirmed that MFYS directly promotes the differentiation of naïve T cells into Tregs, an effect that was counteracted by a PI3K/AKT pathway inhibitor. Molecular docking results suggested that some active components of MFYS can bind to AKT1.

**Conclusions:**

MFYS modulates the immune response in PHN rats, reduces IgG deposition, and ameliorates renal and podocyte injury. It promotes Treg differentiation, regulates the cytokine network, and exerts multi-target effects on both inflammation and metabolism.

**Supplementary Information:**

The online version contains supplementary material available at 10.1186/s13020-025-01272-1.

## Introduction

Membranous nephropathy is classified into primary membranous nephropathy (PMN) and secondary membranous nephropathy, with PMN accounting for 70–80% of all cases [1]. Multiple studies have shown that the incidence of PMN is increasing over the years, particularly among patients with glomerular diseases in China [2]. As one of the most common primary glomerular pathological types, PMN is characterized by diffuse thickening of the glomerular basement membrane and the deposition of immune complexes. Transmission electron microscopy reveals electron-dense deposits between podocytes and the basement membrane, accompanied by podocyte swelling and foot process effacement. Autoantibodies from the circulation bind to target antigens on podocytes, forming in situ immune complexes. These lead to damage of the glomerular filtration barrier, resulting in substantial protein loss in the urine. Consequently, PMN patients often present with hypoalbuminemia and edema. This disease significantly impairs physical endurance, reduces quality of life, and approximately one-third of patients cannot be cured and will progress to end-stage renal disease.

The pathogenesis of PMN is believed to be associated with impaired immune regulatory function, which leads to the production of autoantibodies targeting antigens such as PLA2R [3]. Therefore, how to eliminate pathogenic antibodies has become a key issue in the treatment of PMN. Currently, modern medicine primarily employs immunosuppressive therapy for medium- to high-risk PMN patients, using immunosuppressants such as cyclosporine, tacrolimus, and cyclophosphamide. These drugs reduce antibody production by inhibiting T cell proliferation, thereby promoting podocyte repair [4]. In recent years, anti-CD20 monoclonal antibodies have been rapidly adopted for the treatment of PMN, as they eliminate autoantibodies by depleting a large portion of B cells [5]. However, the use of immunosuppressants significantly increases the risk of severe infections and is associated with a high recurrence rate, with some patients unable to tolerate the side effects. Thus, restoring immune tolerance, reducing recurrence rates, and identifying safer treatment strategies remain major challenges in PMN therapy.

Traditional Chinese Medicine (TCM) serves as a potential treatment option for PMN and is widely used in clinical practice in China. Our team combined Mahuang Fuzi Decoction and Shenzhuo Decoction from *Treatise on Cold Damage and Miscellaneous Diseases* for the treatment of PMN and obtained clinical evidence. A previous cohort study involving 198 PMN patients (84.4% of whom were medium- to high-risk cases) showed a total remission rate of 58.6% at 24 months and 70.0% at 36 months, with 12 patients experiencing adverse events [6]. Notably, patients received only Chinese herbal medicine during the treatment period without the use of immunosuppressants, providing strong evidence for the efficacy and safety of TCM in treating PMN. Preliminary studies also indicated that this formula alleviates podocyte injury in model rats by inhibiting the IL-6/pSTAT3 pathway [7]. Based on clinical experience, we have now optimized the original herbal compound by adding additional herbs, resulting in a formula comprising 10 medicinal ingredients (Mahuang, Fuzi, Huangqi, Fuling, Baizhu, Fangji, Shengjiang, Zexie, Guizhi, Gancao). This compound has been named Mafu Yishen Formula (MFYS) and has demonstrated improved efficacy in clinical practice.

Although MFYS has been used to treat a considerable number of patients clinically, its mechanism of action and core active components remain unclear. The therapeutic profile of MFYS is characterized by a high long-term remission rate and a low recurrence rate, leading us to hypothesize that traditional Chinese medicine may exert its effects by modulating immune tolerance in PMN rather than directly suppressing antibody responses. Investigations into the immunomodulatory cytokine interleukin (IL)-35 have revealed that the level of IL-35 can predict the therapeutic response of our traditional Chinese medicine therapy [8]. This suggests that MFYS may have the potential to enhance immune regulation in PMN. Growing evidence indicates that Treg deficiency is an important pathogenic mechanism in PMN. Multiple studies have shown reduced serum Treg proportions and decreased Foxp3 expression in PMN patients [9, 10]. Given that Tregs can suppress immune responses and antibody production, they have emerged as a therapeutic target for autoimmune diseases. While some researchers have proposed adoptive Treg therapy for PMN [11], its widespread application has been limited by high costs. In this context, traditional Chinese medicine may represent a viable alternative approach.

In this study, the classic PMN animal model—passive Heymann nephritis (PHN) rats—was established and treated with different doses of MFYS. The PHN rat is the most extensively utilized animal model for studying membranous nephropathy. In this model, the disease is induced by the injection of anti-Fx1A serum from immunized sheep, leading to the deposition of heterologous sheep IgG in the subepithelial space of the glomeruli. This event triggers a host humoral immune response, culminating in the production and subsequent deposition of autologous rat IgG. The formation of these immune complexes along the glomerular basement membrane constitutes the hallmark pathological lesions of membranous nephropathy [12]. Experimental results confirmed that MFYS improved biochemical indicators such as urinary protein in the model rats. MFYS also ameliorated renal and podocyte injury in PHN rats. More importantly, MFYS significantly reduced renal IgG deposition while increasing the proportion of Treg cells and modulating cytokine profiles. Integrated spleen transcriptome and proteome analyses revealed that MFYS upregulates the PI3K/AKT pathway and enhances oxidative phosphorylation. Both in vivo and in vitro experiments further validated the role and mechanism of MFYS in promoting Treg differentiation. Among the 26 prototype components of MFYS detected in the blood, we also predicted specific constituents that may bind to AKT1. Based on multi-omics technology, this study elucidates the mechanism by which MFYS treats PMN through immunomodulation and offers a novel therapeutic perspective focused on enhancing immune regulatory capacity for PMN management.

## Materials and methods

### Preparation of MFYS

MFYS consists of ten Chinese herbal medicines listed in Table 1, all sourced from Beijing Hospital of Traditional Chinese Medicine affiliated with Capital Medical University. The traditional decoction method was employed to prepare MFYS: Ephedra (Mahuang) and Prepared Aconite Lateral Root (Zhi Fuzi) were first decocted in water for 0.5 h, after which the remaining herbs were added and further decocted for half an hour. The dregs were then filtered out, and the liquid was retained. The dregs were decocted again for 20 min, and the resulting liquids from both decoctions were combined and concentrated according to the equivalent dose. MFYS high dose (MFYS-H) was prepared using the doses specified in Table 1, while MFYS low dose (MFYS-L) was prepared at half the dose, with separate decoctions for each. Based on clinical dosages for human patients, the rat doses were calculated by body surface area conversion (human clinical dose × equivalent dose coefficient of 6.3). The dosage of MFYS was determined based on our previous clinical and experimental studies [6, 7]. The final oral gavage dose was 1 ml/100 g body weight per day.

**Table 1:** Composition of MFYS

Latin name	Chinese name	English name	Plant part	Weight (g)
Ephedra sinica Stapf	Mahuang	Ephedra	Stem	10
Aconitum carmichaelii Debx	Zhi Fuzi	Prepared Aconite	Tuber	12
Astragalus membranaceus (Fisch.) Bunge	Sheng Huangqi	Raw Astragalus	Root	30
Poria cocos (Schw.) Wolf	Fuling	Poria	Sclerotium	15
Atractylodes macrocephala Koidz	Chao Baizhu	Stir-fried Atractylodes	Root	12
Stephania tetrandra S. Moore	Fangji	Stephania tetrandra	Root	10
Zingiber officinale Roscoe	Shengjiang	Fresh Ginger	Rhizome	10
Alisma orientale (Sam.) Juzep	Zexie	Alisma	Tuber	10
Cinnamomum cassia Presl	Guizhi	Cinnamon Twig	Twig	10
Glycyrrhiza uralensis Fisch	Zhi Gancao	Prepared Licorice	Root	6

Cyclosporin A (CsA) was purchased from Hangzhou Zhongmei Huadong Pharmaceutical Co., Ltd. (Batch No.: FJB1006004, 25 mg/capsule). CsA capsules were dissolved in water and administered via oral gavage at a dose of 25 mg/kg/day, also adjusted for rat equivalent dosing. The control (CTL) group received 1 ml/100 g/day of water. The treatment lasted for 12 weeks.

### Animal model and grouping

Animal experiments were conducted following internationally recognized guidelines and approved by the Animal Ethics Committee of Beijing Institute of Traditional Chinese Medicine. 30 male Sprague–Dawley rats aged 5–6 weeks, weighing 120–140 g, were purchased from Beijing Huafukang Biotechnology Co., Ltd. (License No.: SCXK (Beijing) 2016–0002). Rats were housed in a specific pathogen-free environment with constant temperature (22 ± 2 ℃) and humidity (55 ± 5%), with a 12-h light–dark cycle and free access to food and water. After 3 days of acclimatization, 30 male SD rats were randomly divided into groups: 6 as CTL group, injected with 0.6 ml/100 g normal saline via the tail vein, and 24 injected with 0.6 ml/100 g sheep anti-rat Fx1A antibody serum (PTX-002S, Probetex, USA) via the tail vein. One week after the injection of antibodies or saline, the 24-h urinary protein excretion was measured in all rats. A result greater than 15 mg was considered indicative of successful model establishment. These 24 PHN rats were then randomly divided into four groups: the model group (MOD), CsA, MFYS-L and MFYS-H. This was designated as week 0, and drug intervention was initiated.

### Rat biochemical indicators

24-h urine was collected from rats every 2 weeks, and 24-h urinary protein excretion was measured. After 12 weeks of treatment, blood was drawn from the auxiliary artery under anesthesia with 1% pentobarbital sodium. Serum was obtained after centrifugation (3000 rpm, 4 °C, 10 min). Urinary protein concentration was measured using the CBB method, and 24-h urinary protein (24hUTP) was calculated. Serum biochemical indicators, including albumin (ALB), serum creatinine (Scr), urea nitrogen (UREA), total cholesterol (CHO), triglycerides (TG) and low-density lipoprotein (LDL), were measured using an automatic biochemical analyzer.

### Renal tissue light microscopy and ultrastructural pathology

Rat kidney and spleen tissues were fixed in 4% paraformaldehyde for 48 h, dehydrated, embedded in paraffin, and cut into 3 µm sections. Hematoxylin and eosin (HE), and periodic acid-silver methenamine -Masson trichrome (P + M) staining were performed according to standard protocols. To evaluate the damage to the glomerular filtration barrier, the ultrastructure of the glomerulus was observed using transmission electron microscopy (TEM). After fixing 1–2 mm^3^ of renal cortical tissue, samples were prepared according to standard protocols and then scanned under a TEM at a magnification of 14,000 ×. Three representative non-overlapping digital micrographs were taken for each glomerulus. The thickness of the glomerular basement membrane (GBM) and the width of podocyte foot processes were quantified, following methods consistent with previous studies [7]. The thickness of the GBM was calculated as ∑GBM area/∑GBM length. The data were analyzed using Image J.

### Immunofluorescence and immunohistochemical assays

Paraffin-embedded renal cortical tissues were sectioned into 3-μm-thick slices, and paraffin-embedded splenic tissues were sectioned into 5-μm-thick slices. After complete dewaxing, antigen retrieval was performed using citrate buffer (pH 6.0) at > 95 ℃. Following permeabilization with 1% Triton X-100, the sections were blocked with 5% bovine serum albumin (BSA) solution. Subsequently, the sections were incubated overnight at 4 ℃ with primary antibodies, which included, as needed: rabbit anti-Synaptopodin antibody (Abcam, #ab259976), rabbit anti-Desmin antibody (CST, #5332), rabbit anti-Bcl-6 antibody (BIOSS, #bs-2734R), AF488-labeled goat anti-rat IgG (Abcam, #ab150157), and AF488-labeled donkey anti-sheep IgG (Abcam, #ab150177). For non-fluorescently labeled primary antibodies, AF488-labeled donkey anti-rabbit IgG (H + L) highly cross-adsorbed secondary antibody (Invitrogen, #A21206) was added. After washing off excess primary antibodies, the sections were incubated with secondary antibodies at 37 °C for 1 h, followed by nuclear staining with DAPI. After washing, the sections were mounted with anti-fade mounting medium. For immunohistochemical staining, sections were incubated overnight at 4 °C with rabbit anti-Podocin antibody (Abcam, #ab50339), followed by incubation with an HRP-labeled secondary antibody (Bioss, #SP-00223, Beijing). Finally, DAB development was performed with hematoxylin counterstaining, where brown coloration indicated positive staining. All sections were observed using either a confocal microscope (LSM 900, ZEISS) or a light microscope. The average fluorescence intensity was analyzed with Image J software, with at least 15 fields of view quantified per group.

### Flow cytometry analysis

Lymphocytes were extracted from the spleen tissues of rats or mice according to the kit instructions (DAKEWEI, #7211011). ①For rat Treg cell staining: First, the following surface antibodies were added to the cell suspension: anti-CD45-Pacific Blue (BD, #558108), anti-CD3-FITC (BD, #561801), anti-CD4-APC-Cy7 (BD, #565432), anti-CD25-PE (BD, #554866), and anti-CD8-PE-Cy7 (eBioscience, #A15385). Subsequently, the cells underwent membrane permeabilization and fixation. Next, intracellular staining was performed using the anti-APC-Foxp3 antibody (eBioscience, #17-5773-82). After thorough washing, the samples were analyzed by flow cytometry. The regulatory T cell proportion (Treg%) was defined as the percentage of CD25⁺FOXP3⁺ cells among CD45⁺CD3⁺CD4⁺CD8⁻ cells. ②For in vitro-cultured mouse T cells: After culture completion, the cells were collected, and the following surface antibodies were added to the suspension: anti-CD4-FITC (BioLegend, #100406) and anti-CD25-BV650 (BioLegend, #102038). The cells were stained at room temperature for 20 min, followed by membrane permeabilization and fixation. Then, the cells were incubated with the anti-FOXP3-PE antibody (BioLegend, #126404) at room temperature for 30 min, washed thoroughly, and subsequently detected. The Treg% was defined as the percentage of CD25⁺FOXP3⁺ cells among CD4⁺ T cells. ③For rat B cell staining: The following surface antibodies were added to the cell suspension: anti-CD45RA-APC (BioLegend, #202313) and anti-IgM-FITC (4A BIOTECH, #SMF105-1000), followed by staining at room temperature for 20 min. After washing, the samples were analyzed.

### Enzyme-linked immunosorbent assay (ELISA)

Cytokines in rat serum, including IL-2, IL-6, IL-10, and IL-17 (FineTest, #ER0039, #ER0042, #ER0033, #ER0035), were detected according to the kit instructions.

### Liquid chromatography-mass spectrometry analysis of MFYS crude extract and rat serum

Metabolite detection was performed using UHPLC-Q Exactive HFX (Thermo, USA) on the MFYS crude drug solution, serum from the MFYS-H group, and serum from the MOD group (triplicate measurements). Liquid chromatography parameters: Column: Waters HSS T3 (100 × 2.1 mm, 1.8 μm); Mobile phase: A = ultrapure water (containing 0.1% formic acid), B = acetonitrile (containing 0.1% formic acid); Flow rate: 0.3 mL/min; Column temperature: 40°C; Injection volume: 2 μL; Gradient elution program: 0 min A/B (100:0, v/v), 1 min A/B (100:0, v/v), 12 min A/B (5:95, v/v), 13 min A/B (5:95, v/v), 13.1 min A/B (100:0, v/v), 17 min A/B (100:0, v/v). Mass spectrometry conditions: Sheath gas: 40 arb, Auxiliary gas: 10 arb, Ion spray voltage: + 3000 V/−2800 V, Temperature: 350°C, Capillary temperature: 320 °C. Scanning mode: Full-ms-ddMS^2^, Polarity: positive/negative ion mode. Primary mass scan range (m/z): 70–1050 Da, Primary resolution: 70,000, Secondary resolution: 17,500. Raw data were preprocessed using Progenesis QI software (Waters Corporation, Milford, USA), and metabolite identification was achieved by matching against a self-built traditional Chinese medicine MS/MS database.

### Network pharmacology analysis of MFYS in the treatment of PMN

Based on liquid chromatography-mass spectrometry detection, metabolites commonly present in both the MFYS crude solution and the serum of MFYS-H rats, but absent in MOD rat serum, were identified, leading to the discovery of 26 prototype blood-absorbed components of MFYS. Target genes of these blood-absorbed components were retrieved using the BATMAN-TCM and Binding DB databases. Analysis of the prototype blood-absorbed components across databases yielded 779 target genes. Disease-related genes of PMN were collected from GeneCards, DISGENET, and OMIM, resulting in 4698 genes. The intersection between the target genes of MFYS blood-absorbed components and PMN disease genes identified 373 potential therapeutic targets of MFYS for PMN. Cytoscape was used to visualize the target genes associated with MFYS treatment of PMN, while the STRING database was employed for protein–protein interaction (PPI) analysis. Further analysis using Cytoscape identified core genes among these targets. Gene Ontology (GO) and Kyoto Encyclopedia of Genes and Genomes (KEGG) enrichment analyses were performed on the 373 target genes.

### Transcriptomic and proteomic analysis of rat spleen

Transcriptome sequencing was performed on 3 rat spleen tissues from CTL, MOD and MFYS-H group based on Illumina sequencing platform. After some routine steps and analysis methods of RNA sequencing, including RNA extraction and detection, library construction, computer sequencing and quality control, reference genome comparison and quantitative analysis. Quantitative proteomic analysis of spleen tissues from the MOD and MFYS-H groups was performed using 4D-DIA proteomics technology, following the standard analytical workflow provided by QLBio (Beijing) Co., Ltd. According to the obtained data, we used the software package DESeq2 R (1.16.1) to analyze the genes of DEG (P < 0.05 and log2FoldChange > 0.5), which were regarded as up-regulated genes. Genes with P < 0.05 and log2FoldChange < -0.5 were regarded as down-regulated genes and heat maps were drawn. GO and KEGG enrichment analyses were performed on the differentially expressed genes, with pathways showing a *P* < 0.05 considered statistically significant.

### Western blotting

Protein extracts were obtained from spleen tissues or in vitro cultured cells using RIPA buffer supplemented with protease and phosphatase inhibitors, and protein concentrations were determined via a BCA assay. Equal amounts of protein were separated by 7.5% SDS-PAGE and transferred to PVDF membranes. After blocking with NcmBlot blocking buffer (NCM Biotech), the membranes were incubated overnight at 4°C with primary antibodies, including Rabbit Anti-phospho-PI3 Kinase p110 beta (Ser1070) (Bioss, #bs-6417R), Rabbit Anti-PI3 Kinase p110β (Selleck, #F1205),Rabbit Anti-Phospho-AKT1/ AKT2/ AKT3 (Ser 473/ Ser 474/ Ser 472) (Selleck, #F1644), Rabbit Anti-AKT (Selleck, #F0004), Mouse Anti-PGC1α (Proteintech, #66369-1-Ig), Rabbit Anti-Phospho-Stat3 (Tyr705) (CST, #9145), Rabbit Anti-Stat3(CST, #12640) and Mouse anti-β-actin (Proteintech, #6945). This was followed by incubation with HRP-conjugated secondary antibodies for 1 h at room temperature. Protein bands were visualized using ECL substrate, detected with a chemiluminescence imaging system, and quantified using ImageJ software, with normalization to loading controls (β-actin).

### In vitro culture of mouse T cells

Naive CD4⁺ T cells were isolated from the spleens of 6-week-old male C57BL/6J mice using a Mouse Naive CD4⁺ T Cell Isolation Kit (Miltenyi Biotec, #130-104-453). A culture medium was prepared to establish an in vitro differentiation environment for Treg cells, containing 10% FBS, 2 mM β-ME, 3 μg/ml anti-CD3 antibody, 3 μg/ml anti-CD28 antibody, 5 ng/ml recombinant IL-2, and 10 ng/ml recombinant TGF-β. Serum from the MOD group and the MFYS-H group were designated as control serum (Ctl-S) and MFYS-containing serum (MFYS-S), respectively. After centrifugation, heat inactivation, and sterilization through a 0.22 μm microporous filter, the sera were stored at −20 °C for later use. No rat serum was added to the culture medium of the control group (CTL). The PI3K inhibitor LY294002 was purchased from Bide Pharm. For the cell experiments, the following groups were established: CTL, 2.5% Ctl-S, 2.5% MFYS-S, and 2.5% MFYS-S + LY294002 (10 μM). After 3.5 days of in vitro culture, flow cytometry analysis and protein extraction were performed. Each group was tested in triplicate.

### Molecular docking

The three-dimensional crystal structure of the AKT1 protein was retrieved from the RCSB Protein Data Bank (PDB). The protein structure was preprocessed using AutoDockTools 1.5.7. The three-dimensional structure files of the core active compounds were obtained from the PubChem database and subjected to energy minimization using OpenBabel 3.1.1 under the MMFF94s force field to ensure that the ligand conformation reached the lowest energy state. Semi-flexible molecular docking was performed using AutoDock Vina, and the absolute value of the docking binding energy (ΔG, kcal/mol) was calculated.

### Statistical analysis

Data following a normal distribution are expressed as the mean ± standard deviation, whereas non-normally distributed data are presented as the median and interquartile range. Statistical analyses were performed using GraphPad Prism 10.0 software. Differences between two groups were evaluated using the Student's t-test, while comparisons among multiple groups were analyzed using one-way ANOVA. A P value of less than 0.05 was considered statistically significant.

## Results

### MFYS improves urinary and serum biochemical indicators in PHN rats

**Fig. 1:**
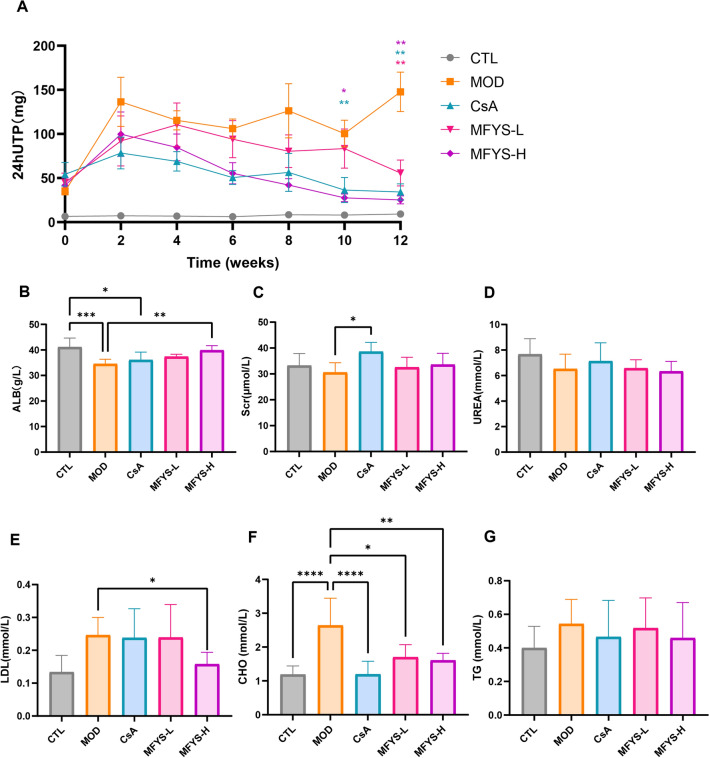
Improvement of biochemical indicators in PHN rats by MFYS. **A**–**G** Urine and serum biochemical indicators of PHN rats in each group (n = 6). Data are presented as mean ± standard deviation. *P < 0.05, **P < 0.01, ***P<0.001, ****P < 0.0001, indicate statistically significant differences between groups. *24hUTP* 24-h urinary total protein, *ALB* serum albumin, *Scr* serum creatinine, *UREA* blood urea nitrogen, *LDL* serum low-density lipoprotein, *CHO* serum total cholesterol, *TG* serum triglycerides

During the 12-week continuous monitoring period, the 24hUTP level in the MOD group remained elevated after 2 weeks, while it gradually decreased in the treatment groups (Fig. 1A). At week 10, both the CsA and MFYS-H groups showed significantly lower 24hUTP levels compared to the MOD group. By week 12, the MFYS-L, MFYS-H, and CsA groups all exhibited significantly lower 24hUTP levels than the MOD group. The ALB level in the MOD group was significantly lower than that in the CTL group, while the CHO level was significantly higher. These alterations were markedly improved in the MFYS-H group (Fig. 1B, F). The LDL level in the MFYS-H group was also significantly lower than that in the MOD group (Fig. 1E). Both the CsA and MFYS-L groups showed significantly lower CHO levels compared to the MOD group (Fig. 1F). No significant differences were observed in Scr and UREA levels between the MFYS-L, MFYS-H, CTL, and MOD groups. However, the CsA group exhibited significantly higher Scr levels than the MOD group (Fig. 1C, D). Additionally, there were no notable differences in TG levels among the groups (Fig. 1G). These results indicate that MFYS-H significantly improved biochemical parameters in PHN rats without nephrotoxicity, demonstrating both efficacy and safety.

### MFYS ameliorates renal lesions and podocyte injury in PHN rats

**Fig. 2:**
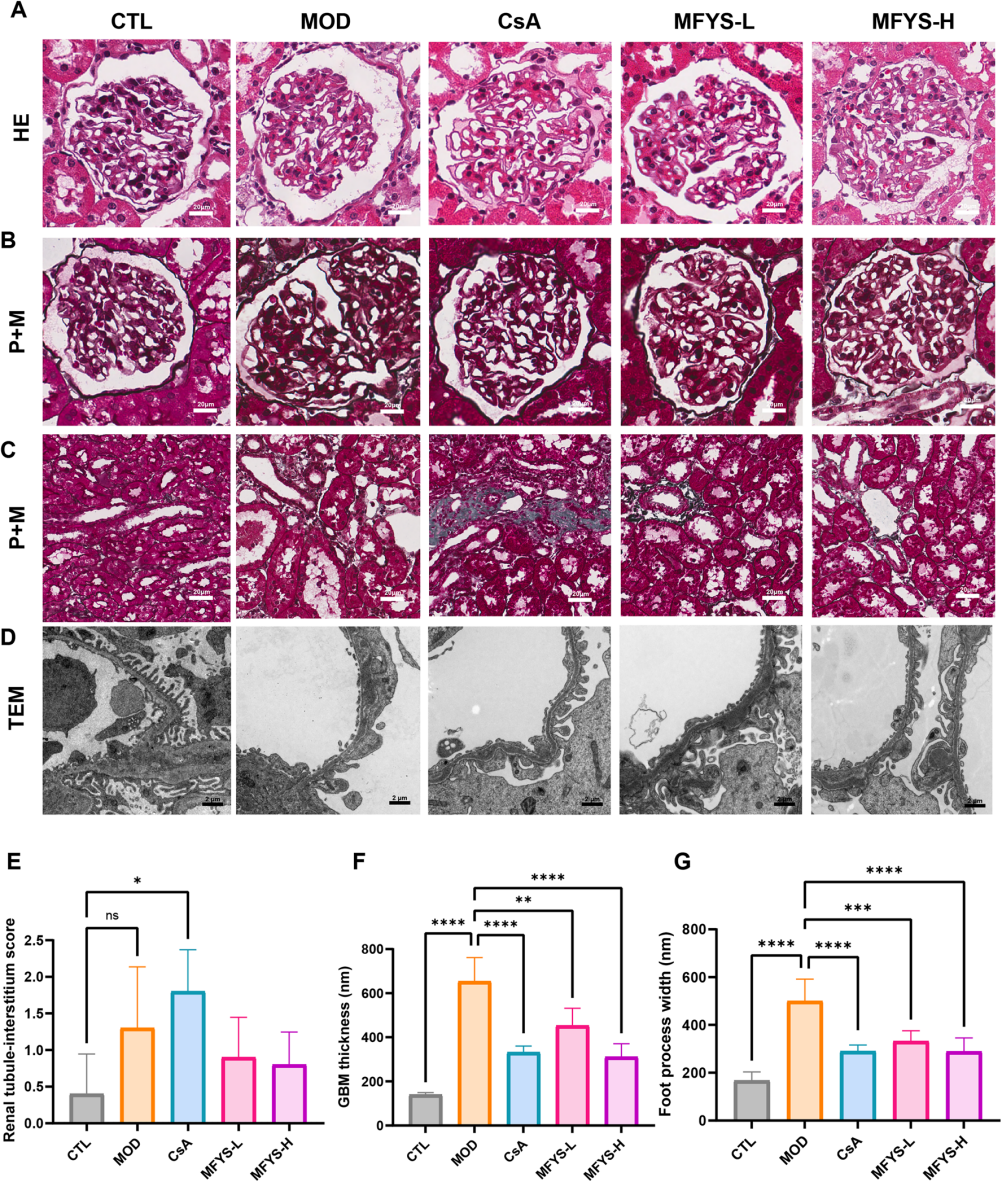
MFYS alleviates renal pathological changes and podocyte injury in PHN (n = 6). **A**–**C** Representative light microscopic pathological images of rats in each group under an optical microscope (× 400), with interstitial injury scores compared (**E**). **D** Representative transmission electron microscopic images of podocyte and basement membrane ultrastructure (× 14,000), with quantification and comparison of basement membrane thickness and podocyte width (**F**, **G**). Data are expressed as mean ± standard deviation. *P < 0.05, **P < 0.01, ***P < 0.001, ****P < 0.0001 indicate statistically significant differences between two groups, and ns indicates no statistically significant difference between two groups

The renal glomerular and interstitial injuries in each group were first observed under light microscopy. HE and P + M staining results showed that the GBM in the MOD group was thickened and appeared rigid, which improved after treatment (Fig. 2A, B). Notably, the CsA group exhibited significant collagen deposition and interstitial damage in the renal interstitium, which may account for the elevated Scr levels in this group (Fig. 2C, E). TEM revealed electron-dense deposits on the GBM, thickened basement membrane, podocyte swelling, and foot process effacement in the MOD group. Both MFYS and CsA treatments significantly reduced GBM thickness and ameliorated foot process swelling (Fig. 2D, F, G).

**Fig. 3:**
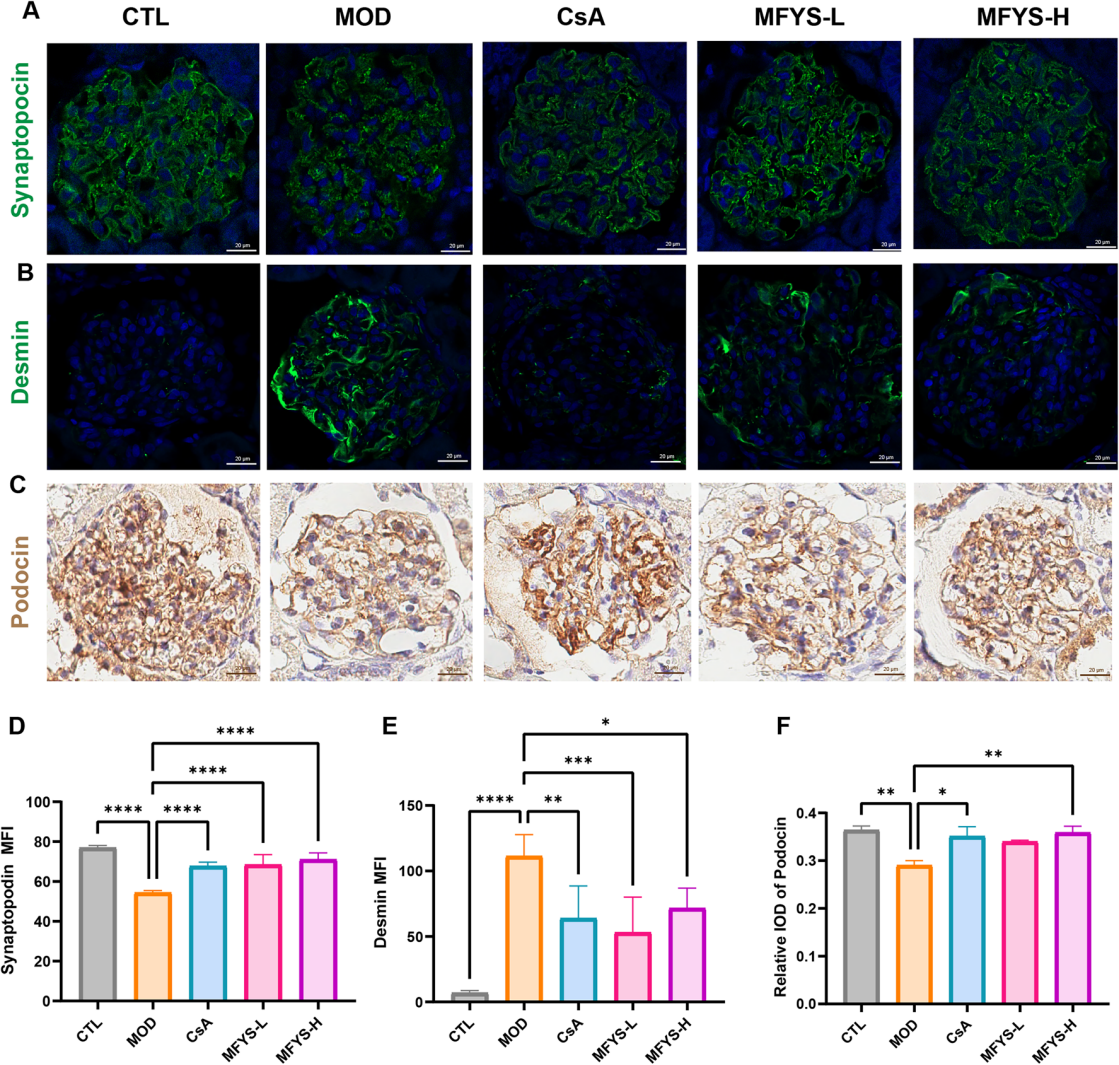
MFYS ameliorates podocyte injury in PHN (n = 6). **A**, **D** Confocal microscopy observation of Synaptopodin staining in glomeruli (green, × 400), with statistical analysis of mean fluorescence intensity (MFI). **B**, **E** Confocal microscopy observation of Desmin staining in glomeruli (green, × 400), with statistical analysis of MFI. **C**, **F** Immunohistochemical staining results of glomerular podocin protein and quantitative analysis of integrated optical density (IOD). Data are expressed as mean ± standard deviation. *P < 0.05, **P < 0.01, ***P < 0.001, ****P < 0.0001 indicate statistically significant differences between the two groups

We further evaluated the expression of podocyte marker proteins. Synaptopodin, an actin-binding protein localized in foot processes that helps maintain the glomerular filtration barrier, was significantly reduced in the MOD group but improved after MFYS and CsA treatment (Fig. 3A, D). Desmin, an intermediate filament protein not expressed in normal podocytes but upregulated upon podocyte injury and dedifferentiation, was significantly increased in the MOD group and decreased after MFYS and CsA treatment (Fig. 3B, E). Podocin, a membrane protein essential for maintaining the slit diaphragm of podocytes, showed reduced expression in the MOD group but was restored in the MFYS-H and CsA groups (Fig. 3C, F). In summary, MFYS significantly alleviated podocyte injury in PHN rats, although the underlying mechanisms require further investigation.

### MFYS reduces rat IgG renal deposition and modulates the immune system in PHN rats

**Fig. 4:**
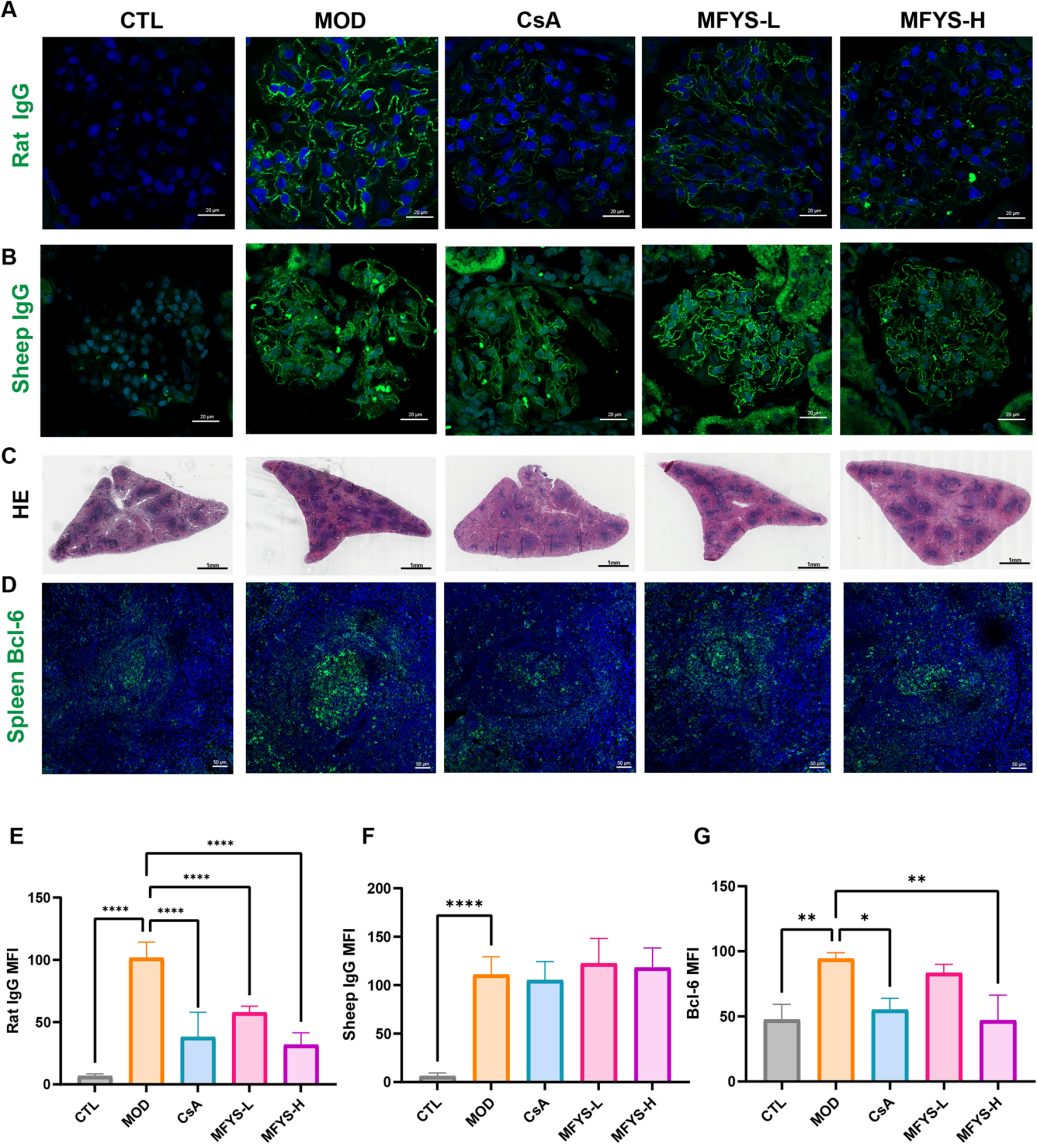
MFYS inhibits antibody response in PHN rats (n = 6). **A**, **B** Representative confocal microscopy images of glomerular rat IgG and sheep IgG staining (green, × 400), with statistical analysis of MFI (**E**–**F**). **C** Representative HE-stained images of rat spleen tissues (× 100). **D**, **G** Representative images of Bcl-6 staining in spleen tissues (green, × 200), with statistical analysis of MFI (**G**). Data are presented as mean ± SD. *P < 0.05, **P < 0.01, ****P < 0.0001 indicate statistically significant differences between groups

Consistent with the pathogenesis of PMN, the injected sheep antibodies act as antigens and bind beneath podocytes, triggering a humoral immune response in rats that leads to the production of autoantibodies, ultimately causing injury to podocytes and the basement membrane. Using immunofluorescence, we detected the levels of rat IgG and sheep IgG deposited in the glomeruli (Fig. 4A, B). The results showed that both MFYS and CsA significantly reduced the deposition of rat IgG, while no statistical difference was observed in sheep IgG levels between the MOD group and the treatment groups (Fig. 4E, F). This result indicates that under consistent modeling conditions, both MFYS and CsA effectively suppressed the production of rat IgG [13]. As the spleen is the largest peripheral lymphoid organ and a major site of antibody response, we further examined the spleens of PHN rats. HE staining revealed that the white pulp structures in the MOD group were more densely organized, which was reduced following treatment (Fig. 4C). Germinal centers in the spleen are critical sites where T cells assist B cells in proliferation and differentiation. Germinal center B cells highly express BCL-6, and its expression level can be used to evaluate the activity of immune responses [14]. As expected, BCL-6 levels were significantly higher in the MOD group compared to the control group and markedly decreased after treatment (Fig. 4D, G). These results demonstrate that both MFYS and CsA inhibited the immune response in PHN rats.

**Fig. 5:**
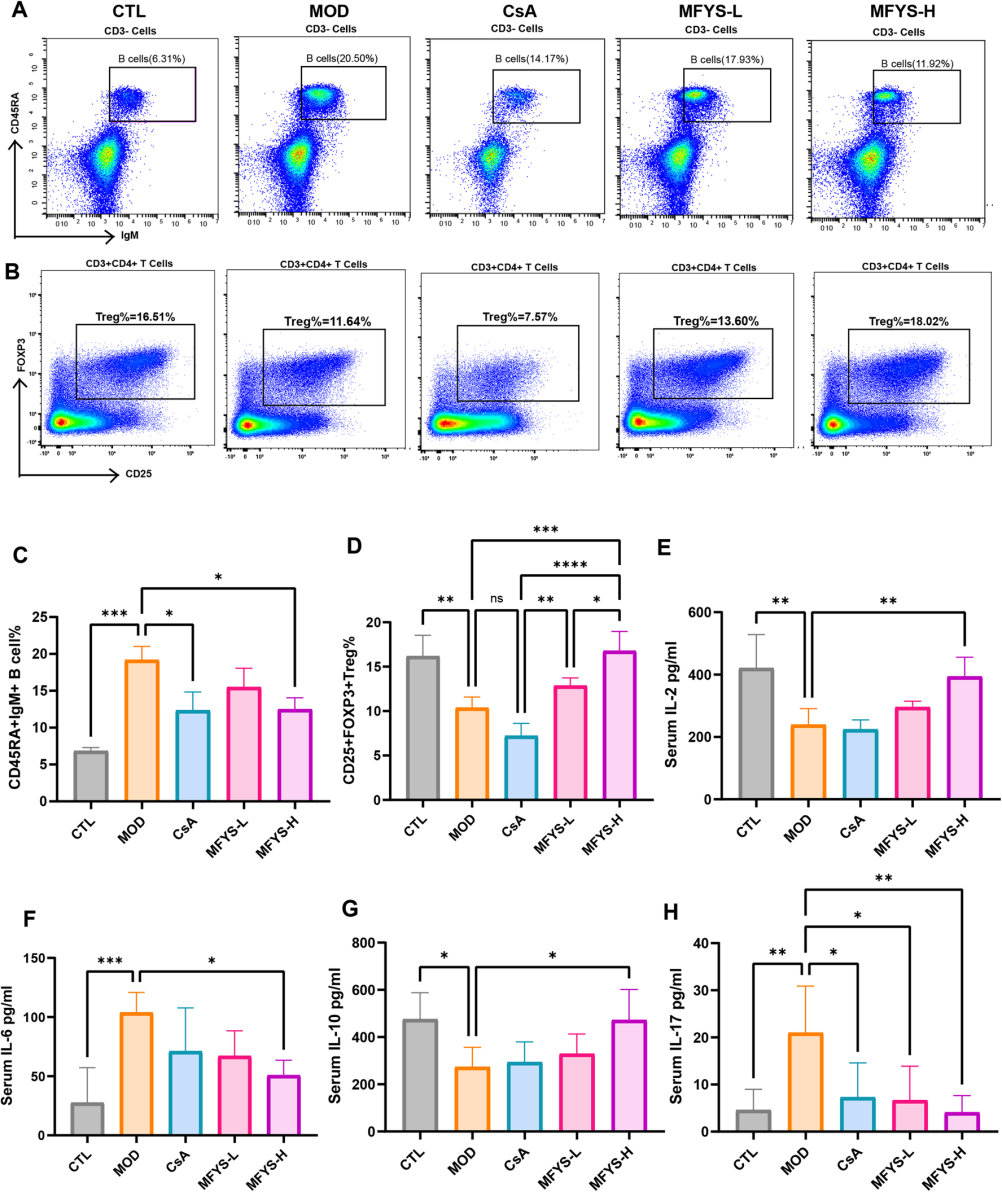
MFYS modulates the immune system in PHN rats (n = 5). **A** Flow cytometric analysis of the percentage of CD45RA + IgM + total B cells among CD3- cells in rat spleen, with statistical analysis shown in (**C**). **B** Flow cytometric analysis of the percentage of CD25 + FOXP3 + Treg cells among CD3 + CD4 + T cells in rat spleen, with statistical analysis shown in (**D**). **E**–**H** ELISA detection and analysis of serum cytokine levels (IL-2, IL-6, IL-10, and IL-17) in each group. Data are expressed as mean ± SD. *P < 0.05, **P < 0.01, ***P < 0.001, ****P < 0.0001 indicate statistically significant differences between groups

To further investigate the specific immunomodulatory effects of MFYS on PHN rats, we examined lymphocyte subsets in the spleen and serum cytokine levels. Flow cytometry results showed that the percentage of CD45RA⁺IgM⁺ total B cells in the MOD group was significantly higher than that in the CTL group, while both the CsA and MFYS-H groups exhibited significantly lower total B cell percentages compared to the MOD group (Fig. 5A, C). Additionally, the proportion of CD25⁺FOXP3⁺ Tregs in the MOD group was significantly lower than that in the CTL group, whereas the MFYS-H group showed a significantly higher Treg percentage than the MOD group. Interestingly, the Treg proportion in the CsA group did not increase compared to the MOD group and was significantly lower than that in both the MFYS-L and MFYS-H groups (Fig. 5B, D). This suggests that enhancing Treg differentiation may represent a unique mechanism through which MFYS suppresses the immune response in PHN rats. Analysis of serum cytokines revealed that MFYS significantly reduced the elevated levels of IL-6 and IL-17 in PHN rats, while upregulating the levels of IL-2 and IL-10 (Fig. 5E–H). The increase in IL-2 may partially contribute to the promotion of Tregs by MFYS.

### Analysis of MFYS blood-absorbed components and network pharmacology predictions

**Fig. 6:**
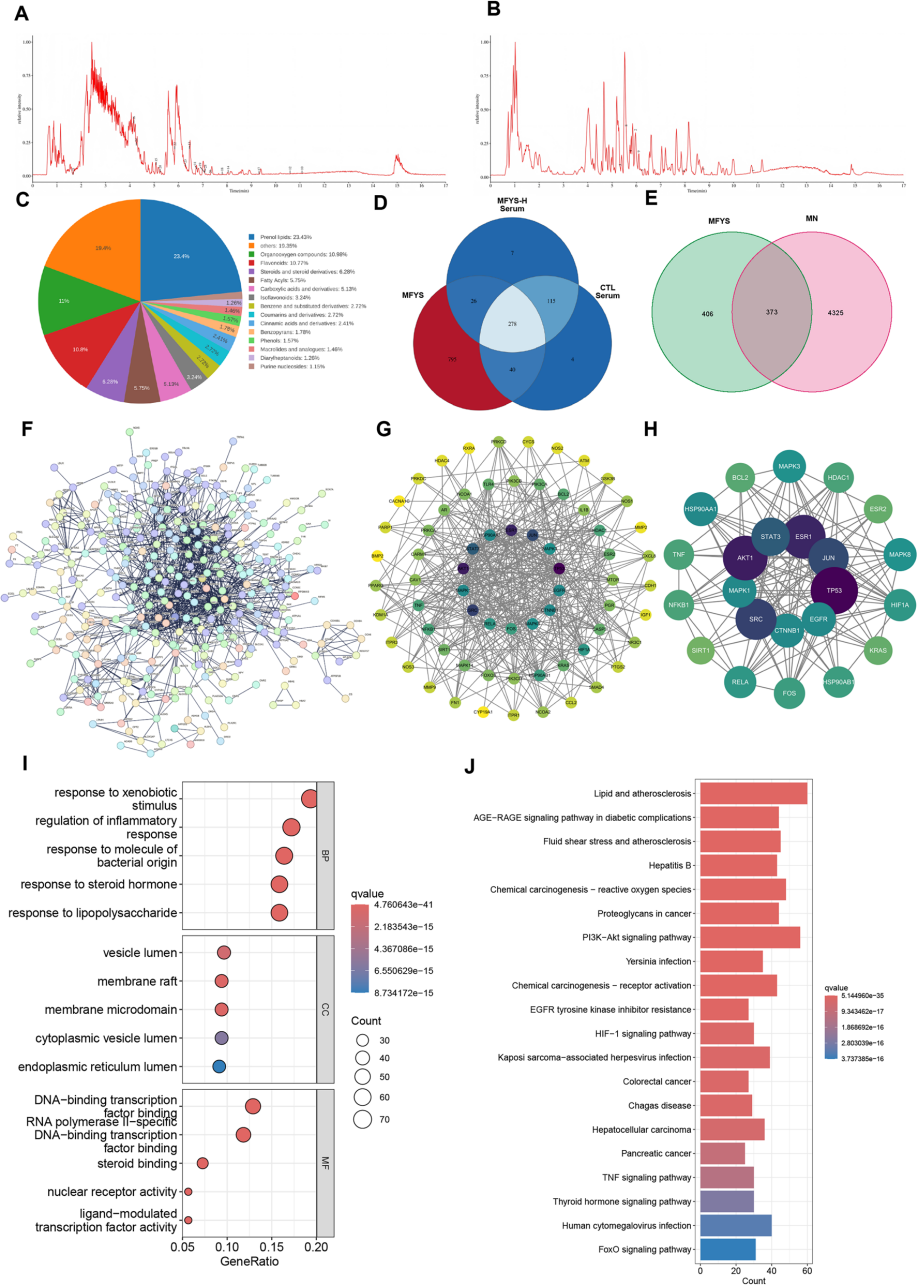
Analysis of MFYS Blood-Absorbed Components and Network Pharmacology Predictions. **A** Base peak ion chromatogram of MFYS detected in positive ion mode. **B** Base peak ion chromatogram of MFYS detected in negative ion mode. **C** Classification statistics of metabolites. **D** Venn diagram of components identified in MFYS crude extract, MFYS-H rat serum, and MOD rat serum. **E** Intersection of potential target genes of MFYS blood-absorbed prototype components and membranous nephropathy disease genes. **F** Protein–protein interaction (PPI) network of target genes related to MFYS treatment of membranous nephropathy. **G**, **H** Two rounds of core gene screening using cytoNCA; darker colors indicate higher functional significance. **I** Bubble plot of GO enrichment analysis for target genes. **J** Bar plot of KEGG enrichment analysis for target genes

To explore the pharmacodynamic material basis and potential molecular mechanisms of MFYS, we conducted component analysis of the crude MFYS extract and serum metabolites from MFYS-H and MOD group rats based on liquid chromatography-mass spectrometry (LC–MS) (Fig. 6A, B, Supplementary Data1). A total of 1139 components were identified in the crude MFYS, 426 types of metabolites were detected in the serum of the MFYS-H group, and 437 metabolites were identified in the MOD group serum. These components were primarily Prenol lipids, Organooxygen compounds, Flavonoids, and Steroids and steroid derivatives (Fig. 6C). After excluding metabolites common to both the MFYS-H and MOD group sera, we identified 26 blood-absorbed components of MFYS (Fig. 6D, Table 2, Supplementary Data2).

**Table 2:** MFYS blood-absorbed components

No	Compound	Mode	RT	Adducts	m/z	ppm
1	alisol c	neg	10.814	M-H	485.327	0.485
2	quercitrin	neg	5.965	M-H	447.094	0.552
3	12-hydroxyjasmonic acid	neg	6.236	M-H + H2O	243.124	2.198
4	alpinone 3-acetate	neg	5.758	M + HCOO	373.093	0.244
5	trans-4-hydroxycyclohexanecarboxylic acid	neg	5.340	M + HCOO	189.077	0.029
6	baicalein	neg	7.946	M-H	269.046	0.189
7	dihydrocucurbitacin f	neg	8.767	M + Cl	555.310	1.023
8	ethyl 3-(4-hydroxyphenyl)propionate	neg	5.575	M + HCOO	239.092	−0.294
9	columbin	neg	6.109	M + CH3COO	417.155	−0.533
10	maslinic acid	pos	11.079	M + H2O + Na	513.356	1.209
11	benzoylhypacoitine	pos	6.766	M + H	574.300	−1.659
12	alisol f	pos	10.594	M + H-H2O	471.346	−1.277
13	tuberosin	pos	6.453	M + NH4-H2O	338.138	−4.183
14	mesaconine	pos	8.041	M + 2CH3CN + H	568.326	4.917
15	3'-geranyl-3-prenyl-2',4',5,7-tetrahydroxyflavone	pos	5.081	M + NH4-H2O	490.264	8.707
16	1-methylinosine	pos	4.170	M + NH4-H2O	282.119	−4.205
17	calycosin	pos	9.313	M + H	285.075	−2.517
18	alisol b	pos	7.796	M + Na	495.346	2.770
19	scoparone	pos	7.076	M + H	207.065	−1.662
20	coclaurine	pos	5.242	M + H2O + Na	326.138	4.419
21	5-deoxy thymidine	pos	1.660	M + 2CH3CN + H	309.154	−7.776
22	isoliquiritigenin	pos	5.852	M + H	257.080	−2.372
23	cinnamaldehyde	pos	6.287	M + H	133.065	−1.408
24	strophanthidin	pos	4.276	M + Na	427.207	−6.298
25	thymol	pos	7.076	M + H-H2O	133.101	−1.199
26	daidzein	pos	7.106	M + H	255.065	−2.123

We retrieved potential intervention targets of the MFYS blood-absorbed components based on database mining, identifying 779 potential targets. Intersection with PMN-related disease genes yielded 373 genes potentially targeted by MFYS (Fig. 6E). A protein–protein interaction (PPI) network was constructed for highly correlated genes among these targets (Fig. 6F). Further screening identified 67 core genes in the first round (Fig. 6G) and 24 core genes in the second round (Fig. 6H), among which AKT1, TP53, ESR1, SRC, JUN, and STAT3 played key roles. Functional enrichment analysis was performed on the 373 genes. In terms of GO enrichment, MFYS was implicated in regulating processes such as response to xenobiotic stimulus, regulation of inflammatory response, and DNA transcription (Fig. 6I). KEGG pathway analysis identified potential signaling pathways involved in MFYS treatment of PMN, including the PI3K-Akt signaling pathway, HIF-1 signaling pathway, TNF signaling pathway, and FoxO signaling pathway (Fig. 6J).

### Transcriptomic analysis reveals key signaling pathways through which MFYS modulates the immune system in PHN

**Fig. 7:**
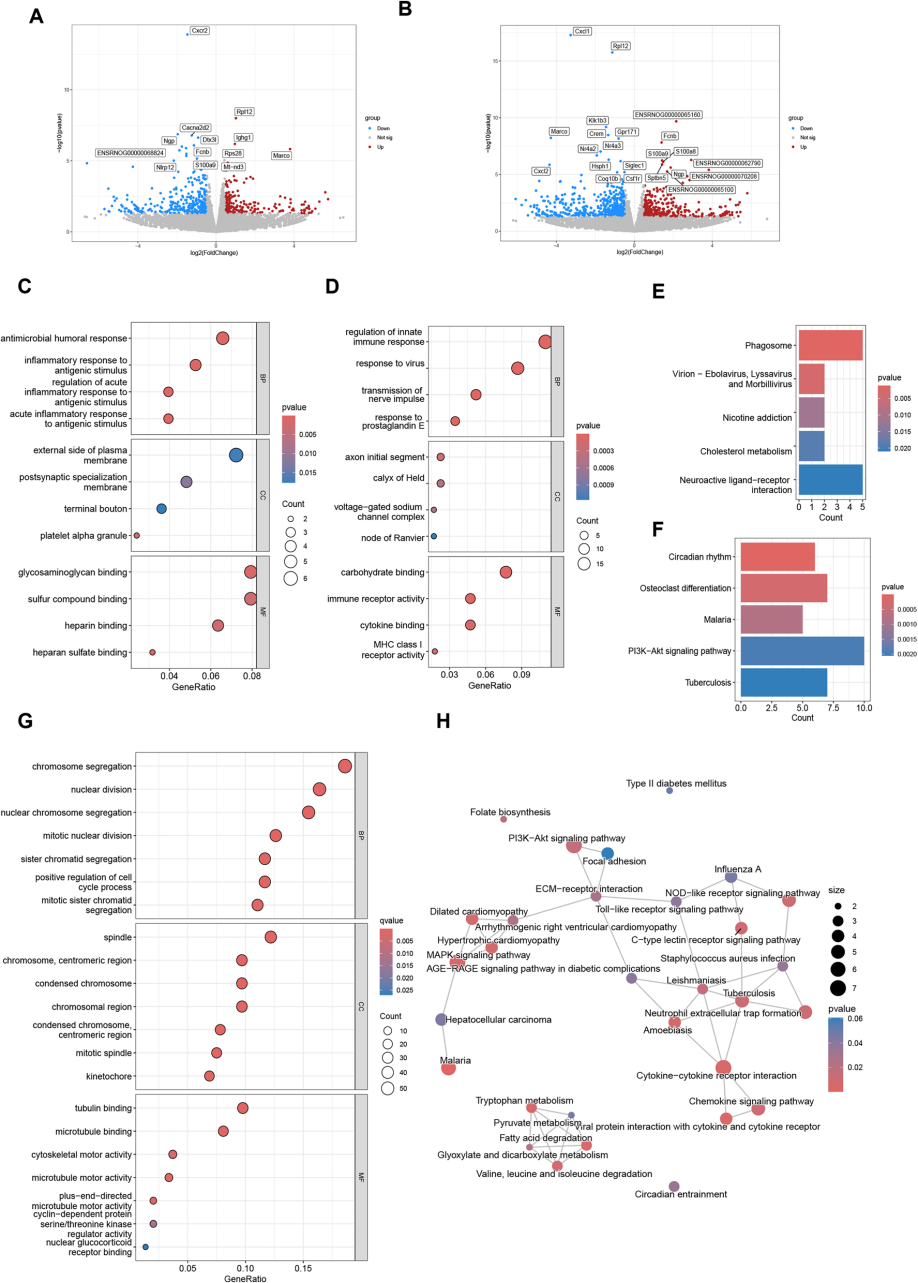
MFYS modulates immune pathways in PHN: insights from splenic transcriptomics. **A** Presents a volcano plot of differentially expressed genes in the MOD group compared to the CTL group, while **B** shows the corresponding volcano plot for the MFYS-H group versus the MOD group. **C** and **D** Display GO enrichment bubble plots for up-regulated and down-regulated genes, respectively, in the MOD group relative to the CTL group. Similarly, **E** and **F** provide KEGG enrichment bar plots for up-regulated and down-regulated genes in the same comparison. **G** Depicts the GO enrichment bubble plot for down-regulated genes in the MFYS-H group compared to the MOD group, and **H** shows the KEGG enrichment bar plot for up-regulated genes in the MFYS-H group versus the MOD group

Transcriptomic analysis was performed on splenic tissues from each group to investigate the mechanisms by which MFYS modulates the immune system in PHN rats, particularly in promoting Treg differentiation. Compared to the CTL group, the MOD group exhibited 197 significantly up-regulated genes and 335 significantly down-regulated genes in the spleen (Fig. 7A). Up-regulated genes included those encoding rat IgG1 (Ighg1) and ribosomal genes involved in protein translation. Enrichment analysis indicated that the up-regulated genes in the MOD group were primarily associated with humoral immunity and inflammatory processes, reflecting the activation of antibody responses in rats (Fig. 7C, E). Down-regulated genes in the MOD group were mainly involved in immune and inflammatory regulation (Fig. 7D). Notably, the PI3K/AKT pathway was down-regulated (Fig. 7F). Compared to the MOD group, the MFYS group showed 345 significantly up-regulated genes and 476 significantly down-regulated genes in the spleen (Fig. 7B). Enrichment analysis revealed that the down-regulated genes in the MFYS group were primarily enriched in chromosome- and cell division-related genes, indicating that MFYS suppressed the proliferation and activation of splenic immune cells (Fig. 7G). Notably, KEGG enrichment analysis demonstrated that the up-regulated genes in the MFYS group were enriched in the PI3K/AKT pathway (Fig. 7H). Additionally, metabolic pathways conducive to Treg cell differentiation, such as fatty acid degradation, were also up-regulated (Fig. 7H). In fact, activation of the PI3K/AKT pathway has been shown to promote Treg differentiation [15]. Therefore, up-regulating the PI3K/AKT pathway may represent a key mechanism through which MFYS enhances Treg cell generation.

### Proteomic analysis reveals MFYS-mediated immune and metabolic regulation in spleen of PHN rats

**Fig. 8:**
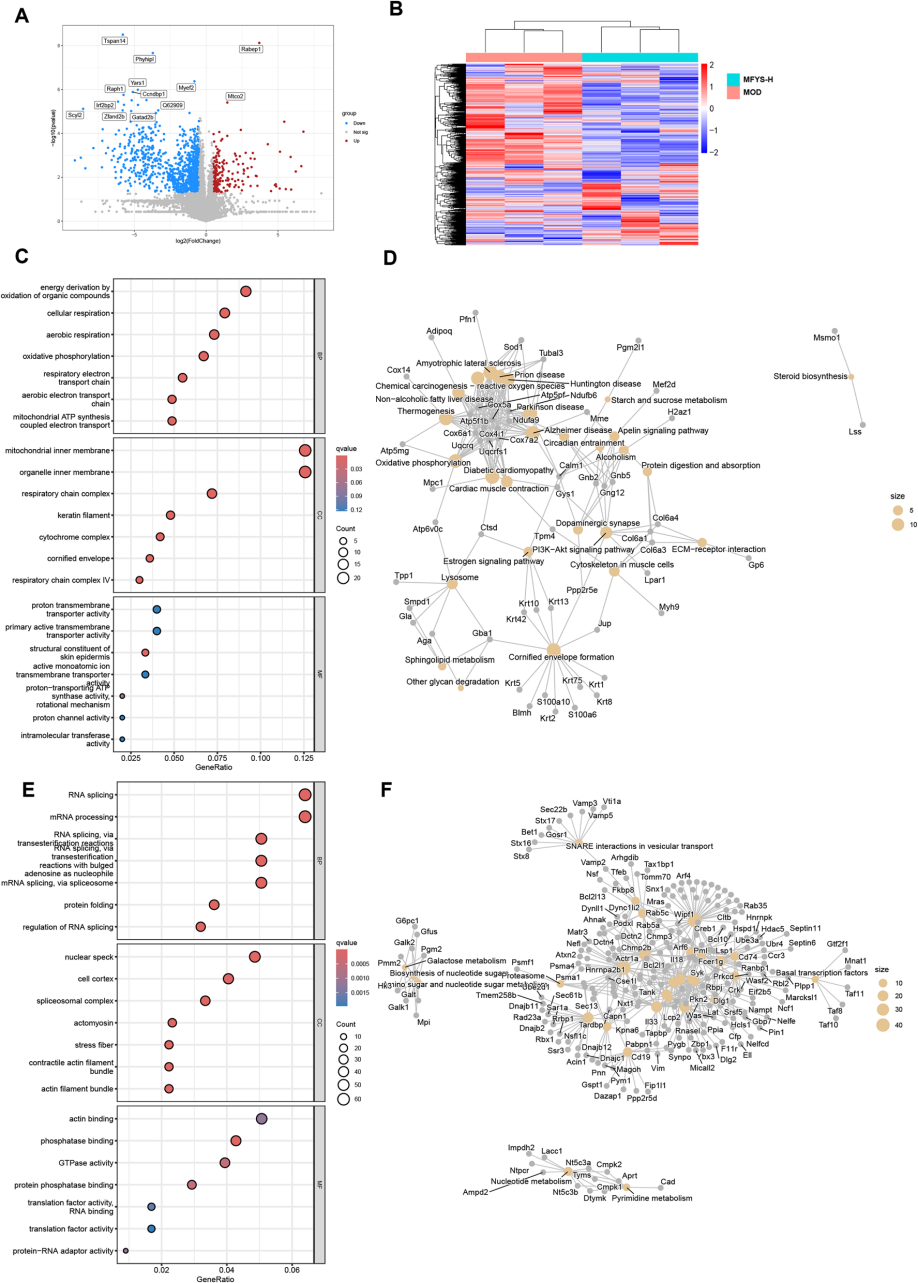
4D-DIA proteomic analysis of rat spleen. **A** Volcano plot of differentially expressed proteins in the MFYS-H group compared to the MOD group; **B** Heatmap of differentially expressed proteins in the MFYS-H group versus the MOD group; **C** GO enrichment bubble plot of up-regulated proteins in the MFYS-H group compared to the MOD group; **D** KEGG enrichment network plot of up-regulated proteins in the MFYS-H group versus the MOD group; **E** GO enrichment bubble plot of down-regulated proteins in the MFYS-H group compared to the MOD group; **F** KEGG enrichment network plot of down-regulated proteins in the MFYS-H group versus the MOD group

To further investigate the mechanisms by which MFYS modulates the immune system in PHN rats, we performed 4D-DIA proteomic analysis on splenic proteins from the MFYS-H and MOD groups. Compared to the MOD group, the MFYS-H group exhibited 201 up-regulated and 1115 down-regulated proteins in the spleen (Fig. 8A, B). GO enrichment analysis indicated that the up-regulated proteins were primarily enriched in mitochondrial oxidative phosphorylation and mitochondrial respiratory chain-related pathways (Fig. 8C). KEGG enrichment analysis also revealed up-regulation of the oxidative phosphorylation pathway (Fig. 8D). In addition, the PI3K − Akt signaling pathway was significantly up-regulated. The down-regulated proteins were mainly enriched in pathways such as transcriptional regulation and Fc gamma R − mediated phagocytosis (Fig. 8E, F), reflecting the inhibitory effect of MFYS on the immune response in the rat spleen.

### Integrated analysis and validation experiments reveal that MFYS upregulates the PI3K/AKT pathway

**Fig. 9:**
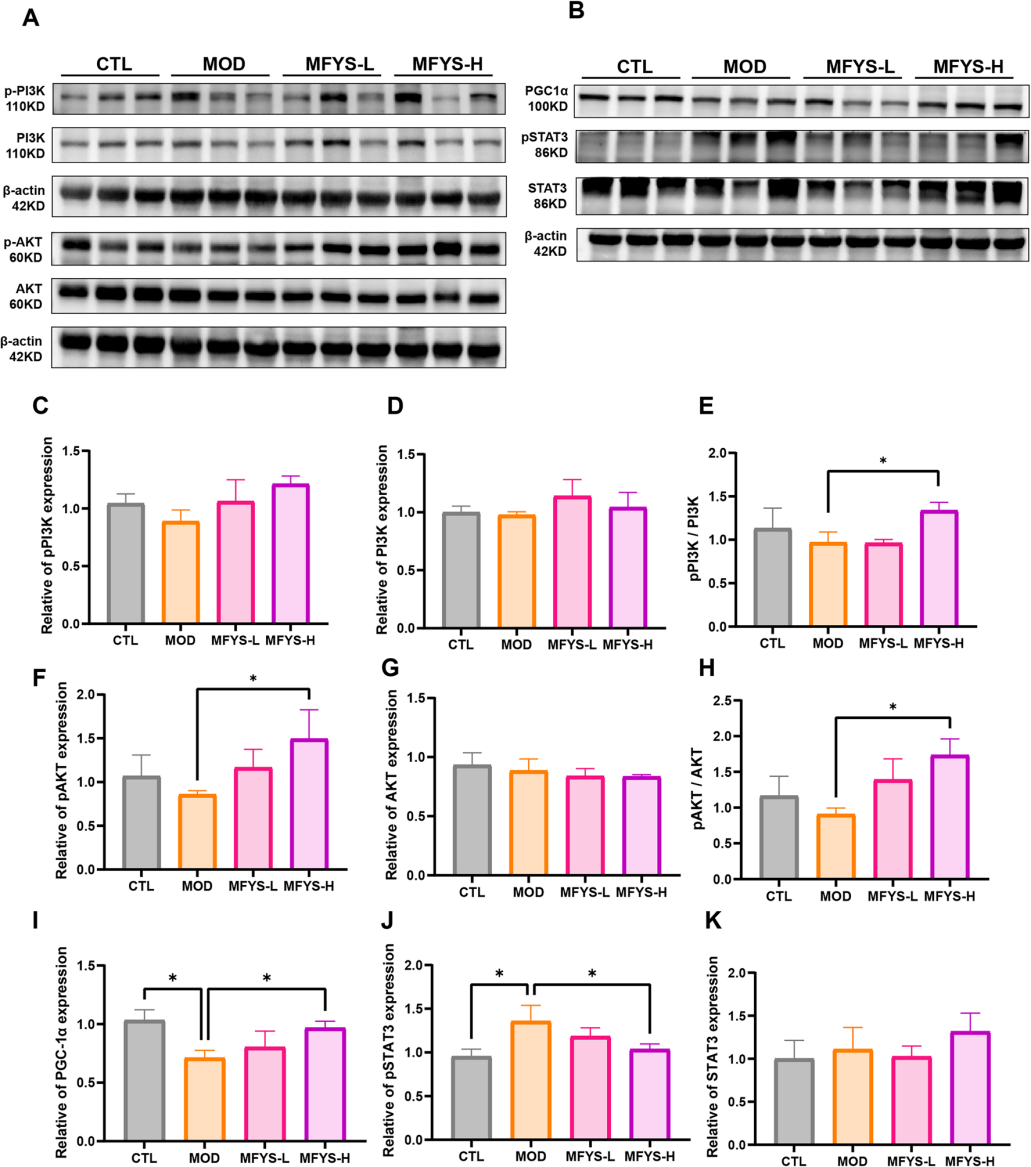
Expression of pathway-related proteins in rat spleen after MFYS intervention. **A** shows Western blot results and statistical analysis (**C**–**H**) of pPI3K, PI3K, pAKT, and AKT protein expression in splenic tissues. **B** shows Western blot results and statistical analysis (**I**–**K**) of PGC-1α, pSTAT3, and STAT3 protein expression. Data are expressed as mean ± SD, with *P < 0.05 indicating statistically significant differences between groups

Based on transcriptomic and proteomic data from rat spleen, we found that MFYS upregulates the PI3K/AKT pathway, which was also enriched in the network pharmacology analysis. Therefore, we hypothesized that the PI3K/AKT pathway may be one of the mechanisms by which MFYS regulates immune function. To verify this hypothesis, we examined the expression levels of proteins related to the PI3K/AKT pathway in rat spleen (Fig. 9A, C–H). The results showed that MFYS increased the ratios of pPI3K/PI3K and pAKT/AKT, confirming its activating effect on this pathway. In addition, given the promoting effect of MFYS on mitochondrial oxidative phosphorylation and its anti-inflammatory properties, we also assessed the expression of PGC-1α (a key regulator of mitochondrial biogenesis) and pSTAT3/STAT3 (a pro-inflammatory transcription factor) (Fig. 9B, I–K). The findings demonstrated that MFYS enhanced PGC-1α expression while suppressing STAT3 phosphorylation.

### MFYS promotes Treg differentiation via upregulation of the PI3K/AKT pathway in vitro

**Fig. 10:**
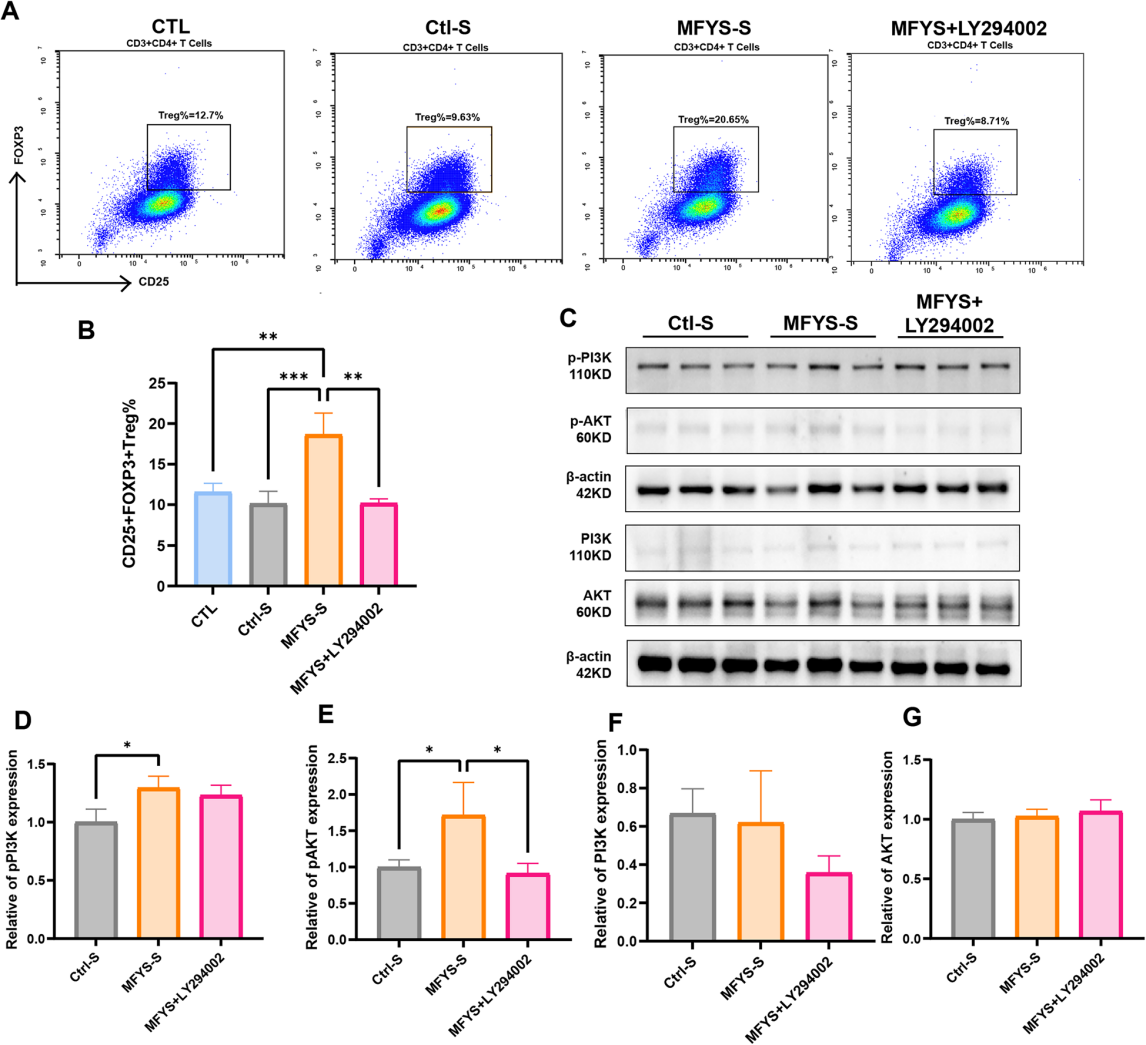
MFYS Promotes Treg Differentiation by Activating the PI3K/AKT Pathway In Vitro. **A** Representative flow cytometry gating plots and quantitative analysis (**B**) of the CTL, Ctl-S, MFYS-S, and MFYS-S + LY294002 groups. **C** Western blot results and statistical analysis (**D**–**G**) of pPI3K, PI3K, pAKT, and AKT protein expression in T cells across groups. Data are expressed as mean ± SD. *P < 0.05, **P < 0.01, indicates a statistically significant difference between groups

To further investigate the direct effect and mechanism of MFYS on Treg cell differentiation, we conducted in vitro experiments under various intervention conditions to polarize naive mouse T cells into Tregs. The results showed that MFYS-S significantly increased the proportion of Treg differentiation compared to the Ctl-S group (Fig. 10A, B), an effect that was abolished by the PI3K/AKT pathway inhibitor LY294002. We also examined the activation status of the PI3K/AKT pathway in each group of cells (Fig. 10C). As expected, MFYS-S activated the PI3K/AKT pathway in T cells, while this activation was inhibited by LY294002 (Fig. 10D–G). These data demonstrate that MFYS promotes Treg differentiation by activating the PI3K/AKT pathway.

### Molecular docking of MFYS blood-absorbed components with AKT1

Combining the previous network pharmacology analysis with in vivo and in vitro experiments, we found that MFYS promotes Treg cell differentiation by activating the PI3K/AKT pathway. The activation of AKT was particularly evident in in vitro experiments, and AKT1 is the predominant subtype functioning in Treg cells. Therefore, we hypothesized that the blood-entering components of MFYS might bind to the AKT1 protein and conducted molecular docking experiments to verify this. The results showed that the binding energies of components such as columbin, quercitrin, benzoylhypacoitine, and calycosin with AKT1 were all less than -6 kcal/mol (Supplementary Data 3). Among them, quercitrin was validated using a standard compound (Supplementary Data 4), further confirming its binding reliability.​​

## Discussion

This study found that MFYS significantly improved biochemical indicators and renal injury in PHN rats, suggesting its potential therapeutic effect on PMN. Compared with the conventional immunosuppressant CsA, MFYS not only exhibited similar or even superior effects in ameliorating renal pathological damage and podocyte injury, but also demonstrated distinct characteristics in its mechanism of action. Notably, MFYS does not simply suppress the immune response; instead, it functions by modulating immune tolerance. Specifically, it promotes Treg differentiation while restoring the balance between pro-inflammatory and anti-inflammatory cytokines, thereby inhibiting the humoral immune response and reducing antibody-mediated injury to podocytes. Integrating network pharmacology, multi-omics analysis, and in vivo and in vitro mechanistic validation, we revealed the central role of the PI3K/AKT signaling pathway in the immunomodulatory effects of MFYS, and further predicted that certain serum-absorbed components may directly target AKT1. In summary, MFYS, as an immune tolerance-oriented therapeutic approach, offers a novel strategy for the treatment of PMN.

Currently, the standard treatment for PMN primarily relies on immunosuppressants, including rituximab, calcineurin inhibitors, and cyclophosphamide. Although these drugs can reduce antibody production and induce remission, they have significant limitations: numerous toxic side effects, poor tolerability, and a high long-term relapse rate. In fact, studies have shown that an increase in patient Treg% can predict the therapeutic efficacy of rituximab [16]. Treg cells, as central players in mediating immune tolerance, are reduced in number or functionally impaired in various autoimmune diseases, including PMN [17]. Studies have also indicated that patients with chronic kidney disease commonly exhibit low levels of IL-2 and Treg deficiency [9, 10]. Traditional immunosuppressants primarily inhibit T cells, impairing T cell help to B cells, thereby reducing antibody production. However, this suppression is not specific to T cell subsets but rather constitutes broad inhibition of T cells, inevitably reducing Tregs and resulting in a high relapse rate. For example, as a calcineurin inhibitor, the core mechanism of CsA is to block T cell activation by inhibiting the nuclear factor of activated T cells pathway, which is a broad-spectrum suppression of T cell function. However, the homeostasis maintenance and function of Treg cells also depend to some extent on calcineurin signaling. Therefore, mechanistically speaking, CsA not only fails to promote Treg cells but may even adversely affect their function. Thus, MFYS appears to function more as an immune modulator rather than a mere immunosuppressant, potentially helping to reduce the risk of relapse and providing longer-term efficacy, which highlights the advantage of traditional Chinese medicine in treating autoimmune diseases.

In this study, multi-omics results and validation experiments consistently indicate that the PI3K/AKT pathway is a key node through which MFYS modulates immune responses. Studies have shown that activation of the PI3K/AKT pathway can promote Treg differentiation [15, 18, 19]. Potential mechanisms involved include: (a) maintaining and enhancing the expression of Foxp3, the master transcription factor of Tregs; (b) promoting mitochondrial energy metabolism and participating in metabolic reprogramming conducive to Treg differentiation; (c) influencing IL-2 signaling pathway activation and facilitating IL-2-dependent Treg differentiation [20]. This is highly consistent with the observations in this study that after MFYS intervention, serum IL-2 levels were up-regulated in rats, along with enhanced mitochondrial oxidative phosphorylation and increased PGC-1α expression. Meanwhile, MFYS inhibited STAT3 activation and cytokines such as IL-6 and IL-17, thereby reducing inflammatory responses and modulating humoral immune responses from multiple angles.

Although the findings are significant, this study has certain limitations. First, although the PHN model can simulate some pathological processes of PMN, it still falls short of fully reflecting the heterogeneity of the patient population, particularly the pathogenesis of their autoimmune responses. Second, despite the identification of 26 prototype blood-absorbed components of MFYS, further analysis is required to determine which specific component or components play a key role. Of course, multi-component and multi-target are also among the characteristics and advantages of traditional Chinese medicine treatment. Third, although this study employed multi-omics approaches, limitations in technology and tissue sources made it difficult to precisely pinpoint specific cellular subpopulations. While the multi-omics results consistently pointed to the PI3K/AKT pathway, the present work primarily revealed correlation changes across different omics levels, and the precise regulatory network among the transcriptome, proteome, and metabolome remains to be further elucidated. Although we explored the potential MFYS blood-absorbed components interacting with AKT1 through molecular docking analysis, further experimental validation is still lacking. The therapeutic mechanisms of traditional Chinese medicine formulas are typically characterized by multi-component and multi-target interactions, and the material basis underlying their efficacy remains to be further elucidated [21]. Therefore, MFYS may also modulate immune responses in PHN through additional mechanisms.

In the in vitro experiments, we aimed to verify the effect of MFYS on promoting Treg differentiation. At present, there is no well-established mouse model that mimics membranous nephropathy, and technical limitations make the isolation of naïve CD4⁺ T cells from rats more challenging. Therefore, murine naïve CD4⁺ T cells were used for in vitro differentiation assays, a widely accepted practice in immunological research. Importantly, the differentiation mechanism of Treg cells and the PI3K/AKT signaling pathway are highly conserved between rats and mice, supporting the translatability of our findings. Although LY294002 is a commonly used inhibitor of the PI3K/AKT pathway in T-cell studies, it may also have off-target effects on other kinases such as mTOR. The experimental design was established based on previously published protocols and related studies [15, 22]. Overall, the Treg polarization assay serves as a supplementary experiment to elucidate the mechanism by which MFYS promotes Treg differentiation. Together, these findings highlight the therapeutic potential of MFYS as a novel immune-modulating approach for PMN.

## Conclusion

**Fig. 11:**
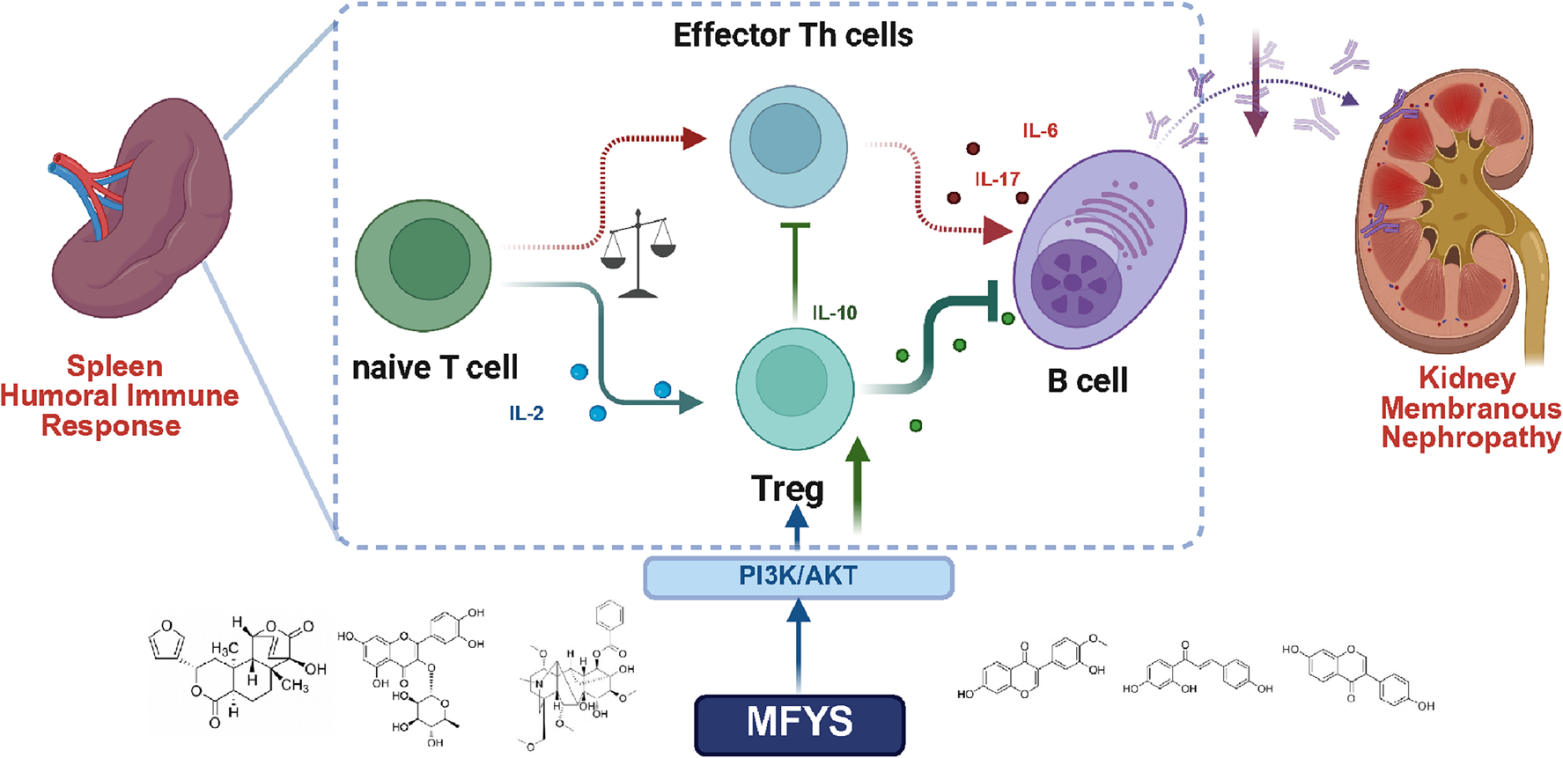
MFYS treats membranous nephropathy by promoting Treg differentiation. (Created in https://BioRender.com)

In summary, MFYS exerts its therapeutic effects on membranous nephropathy in the PHN model by promoting Treg differentiation and reducing rat IgG-mediated podocyte injury, which is associated with the activation of the PI3K/AKT signaling pathway (Fig. 11). This mechanism differs from the traditional "immunosuppressive mode" and aligns more closely with "immune tolerance reconstruction." This study not only provides experimental evidence supporting the application of MFYS but also proposes a novel concept for the treatment of PMN, namely achieving long-term disease control through enhanced immunoregulatory capacity and enabling safer and more effective interventions.

## Supplementary Information

Supplementary material 1.

Supplementary material 2.

Supplementary material 3.

Supplementary material 4.

## Data Availability

Data is provided within the manuscript or supplementary information files. Data will be made available on request.

## Abbreviations

PMN: Primary membranous nephropathy
MFYS: Mafu Yishen Formula
Treg: Regulatory T cells
CsA: Cyclosporine A
PI3K: Phosphoinositide 3 Kinase
TCM: Traditional Chinese Medicine
24hUTP: 24-Hour urinary total protein
ALB: Albumin
Scr: Serum creatinine
UREA: Urea nitrogen
CHO: Total cholesterol
TG: Triglycerides
LDL: Low-density lipoprotein
HE: Hematoxylin and eosin
P + M: Periodic acid-silver methenamine-Masson trichrome
TEM: Transmission electron microscopy
GBM: Glomerular basement membrane
PPI: Protein–protein interaction
GO: Gene Ontology
KEGG: Kyoto Encyclopedia of Genes and Genomes
