# Supplementary figures and images for: Mafu Yishen Formula ameliorates membranous nephropathy by promotion of regulatory T cell differentiation: a multi-omics study
Source: Chin Med. 2026 Jan 6;21:5. doi: 10.1186/s13020-025-01272-1 (PMC12771757; doi:10.1186/s13020-025-01272-1)

**Supplementary data 1**


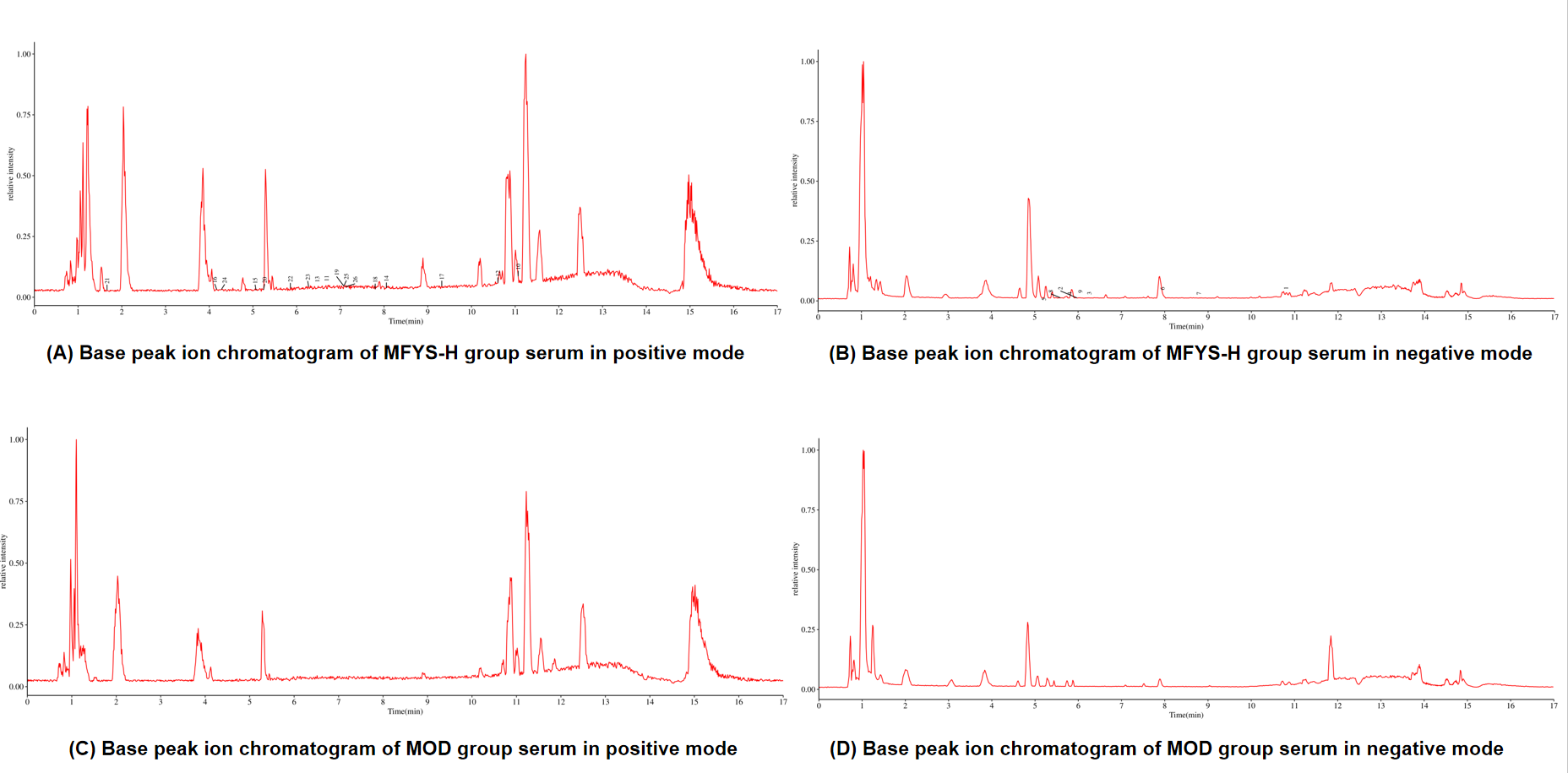

Supplement: Supplementary file 1 — Supplementary material 1. [file 13020_2025_1272_MOESM1_ESM.docx]

# Quercetin

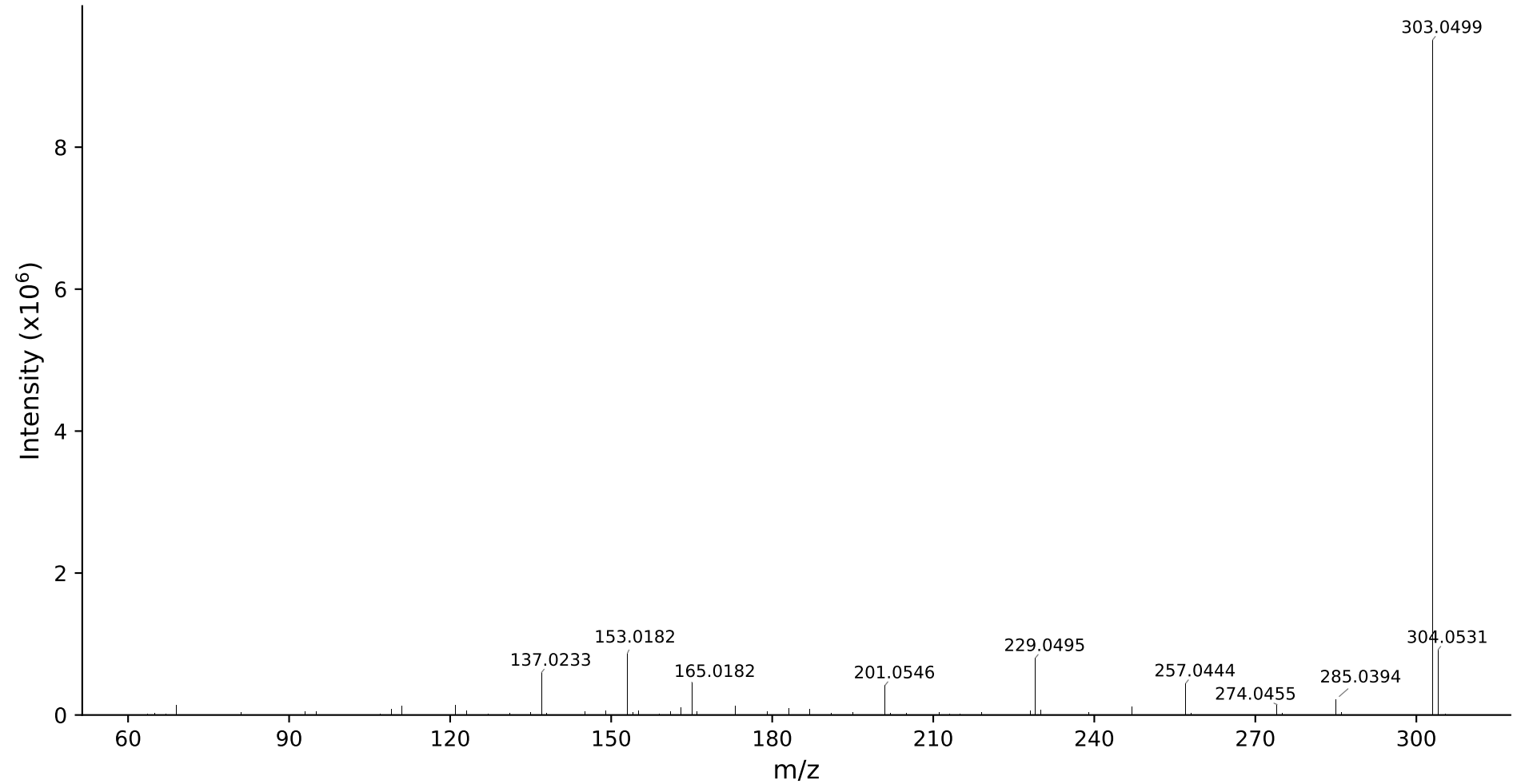

Supplement: Supplementary file 4 — Supplementary material 4. [file 13020_2025_1272_MOESM4_ESM.zip › pos.Quercetin.pdf]
