# Supplementary material for: Mafu Yishen Formula ameliorates membranous nephropathy by promotion of regulatory T cell differentiation: a multi-omics study
Source: Chin Med. 2026 Jan 6;21:5. doi: 10.1186/s13020-025-01272-1 (PMC12771757; doi:10.1186/s13020-025-01272-1)
Supplement: Supplementary file 3 — Supplementary material 3. [file 13020_2025_1272_MOESM3_ESM.docx]

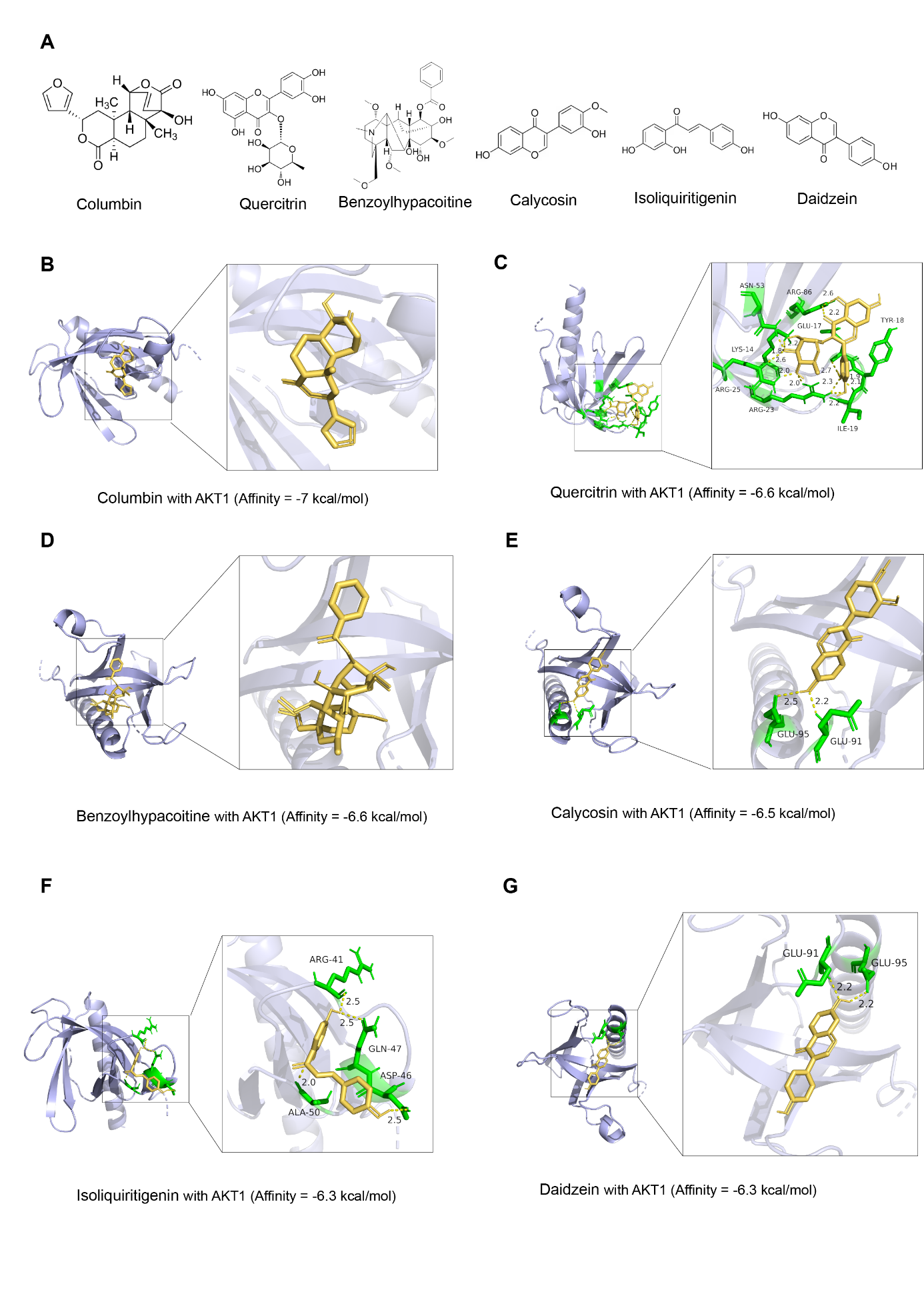


Molecular docking results of representative MFYS components. (A) Chemical structural diagrams of six representative MFYS blood-entering components. (B-G) Visualization images of molecular docking results between MFYS components and AKT1.
