# Supplementary material for: Mafu Yishen Formula ameliorates membranous nephropathy by promotion of regulatory T cell differentiation: a multi-omics study
Source: Chin Med. 2026 Jan 6;21:5. doi: 10.1186/s13020-025-01272-1 (PMC12771757; doi:10.1186/s13020-025-01272-1)
Supplement: Supplementary file 2 — Supplementary material 2. [file 13020_2025_1272_MOESM2_ESM.zip › pos.Sinomenine.pdf]

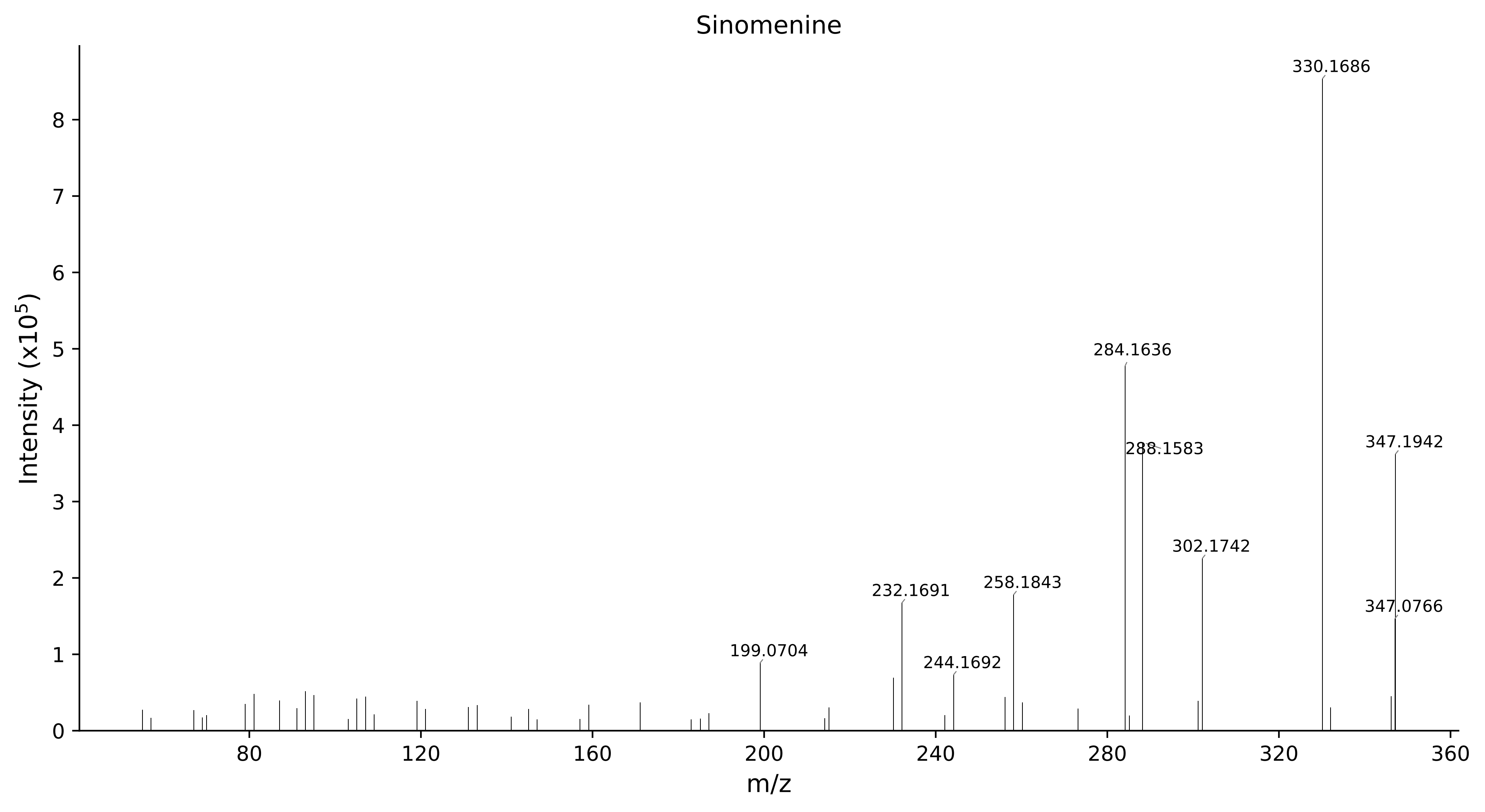

Sinomenine
Intensity (x10^5)
m/z
330.1686
284.1636
288.1583
347.1942
302.1742
232.1691
258.1843
347.0766
199.0704
244.1692
0
1
2
3
4
5
6
7
8
80
120
160
200
240
280
320
360
