# Supplementary material for: Mafu Yishen Formula ameliorates membranous nephropathy by promotion of regulatory T cell differentiation: a multi-omics study
Source: Chin Med. 2026 Jan 6;21:5. doi: 10.1186/s13020-025-01272-1 (PMC12771757; doi:10.1186/s13020-025-01272-1)
Supplement: Supplementary file 2 — Supplementary material 2. [file 13020_2025_1272_MOESM2_ESM.zip › pos.Atractylenolide_III.pdf]

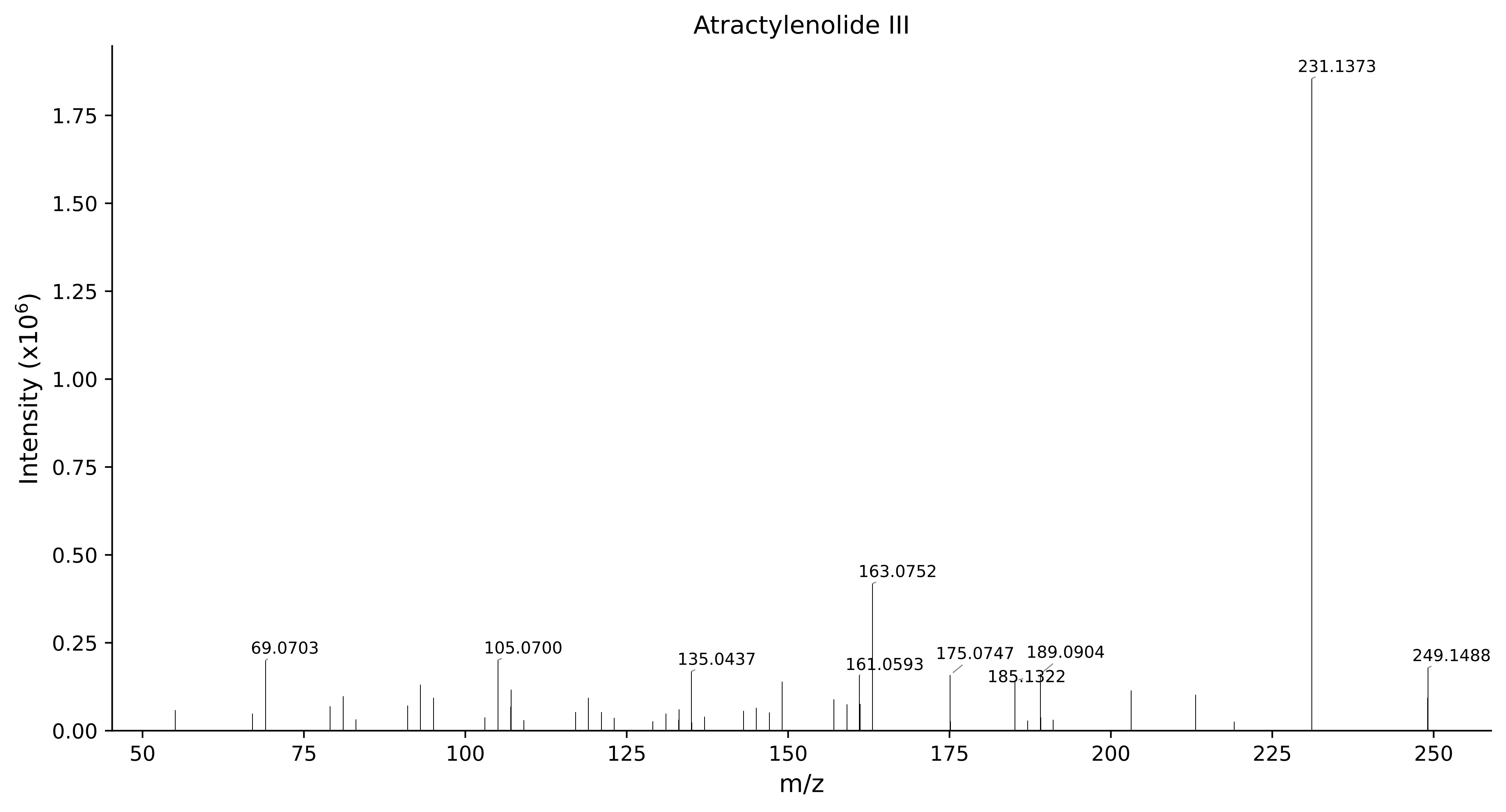

Atractylenolide III
Intensity (x10^6)
m/z
0.00
0.25
0.50
0.75
1.00
1.25
1.50
1.75
50
75
100
125
150
175
200
225
250
231.1373
163.0752
69.0703
105.0700
135.0437
161.0593
175.0747
185.1322
189.0904
249.1488
