# Supplementary material for: Mafu Yishen Formula ameliorates membranous nephropathy by promotion of regulatory T cell differentiation: a multi-omics study
Source: Chin Med. 2026 Jan 6;21:5. doi: 10.1186/s13020-025-01272-1 (PMC12771757; doi:10.1186/s13020-025-01272-1)
Supplement: Supplementary file 2 — Supplementary material 2. [file 13020_2025_1272_MOESM2_ESM.zip › pos.Sibiricaxanthone_B.pdf]

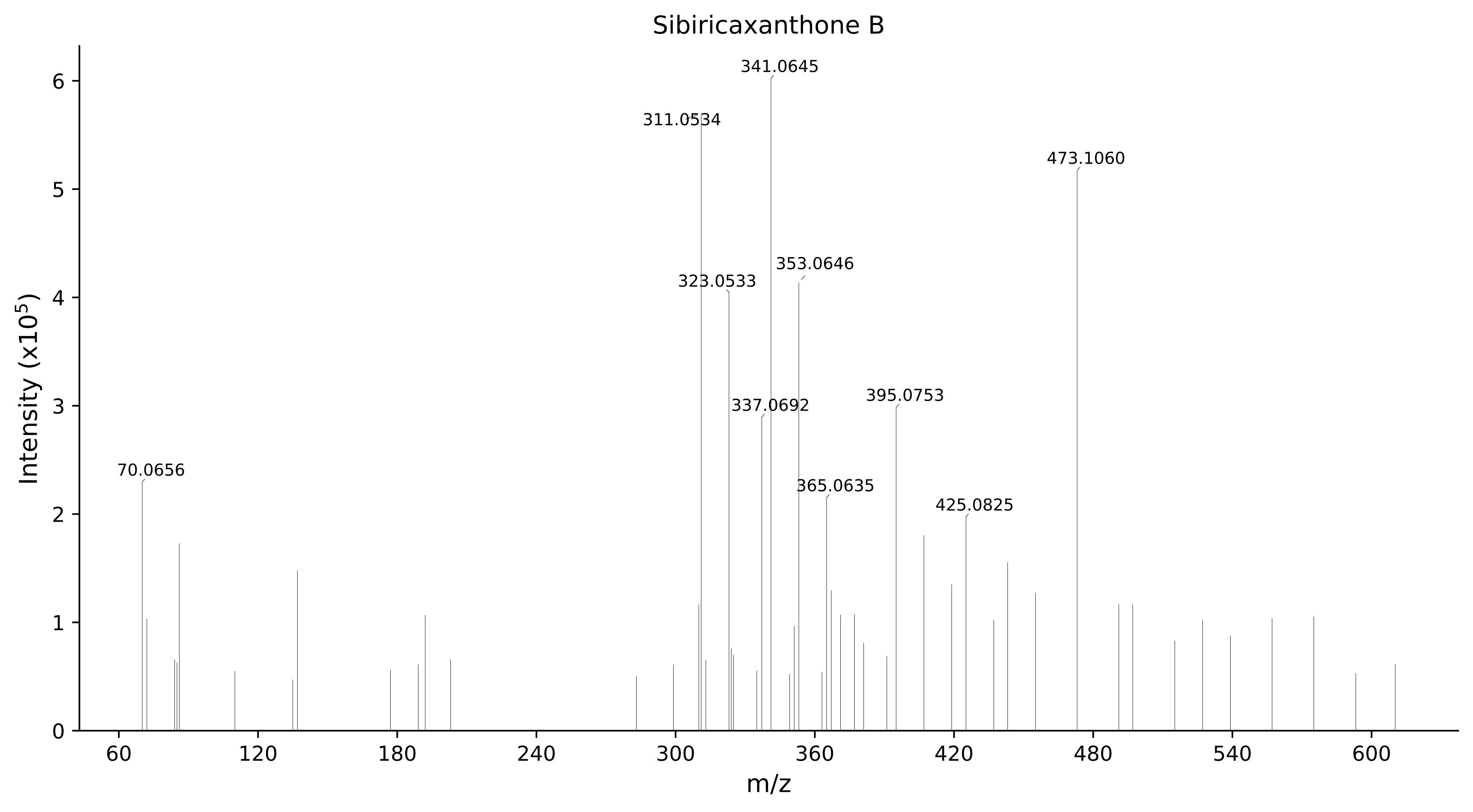
Sibiricaxanthone B
Intensity (x10^5)
m/z
0
1
2
3
4
5
6
60
120
180
240
300
360
420
480
540
600
70.0656
311.0534
323.0533
337.0692
341.0645
353.0646
365.0635
395.0753
425.0825
473.1060
