# Supplementary material for: Mafu Yishen Formula ameliorates membranous nephropathy by promotion of regulatory T cell differentiation: a multi-omics study
Source: Chin Med. 2026 Jan 6;21:5. doi: 10.1186/s13020-025-01272-1 (PMC12771757; doi:10.1186/s13020-025-01272-1)
Supplement: Supplementary file 2 — Supplementary material 2. [file 13020_2025_1272_MOESM2_ESM.zip › pos.Psoralenoside.pdf]

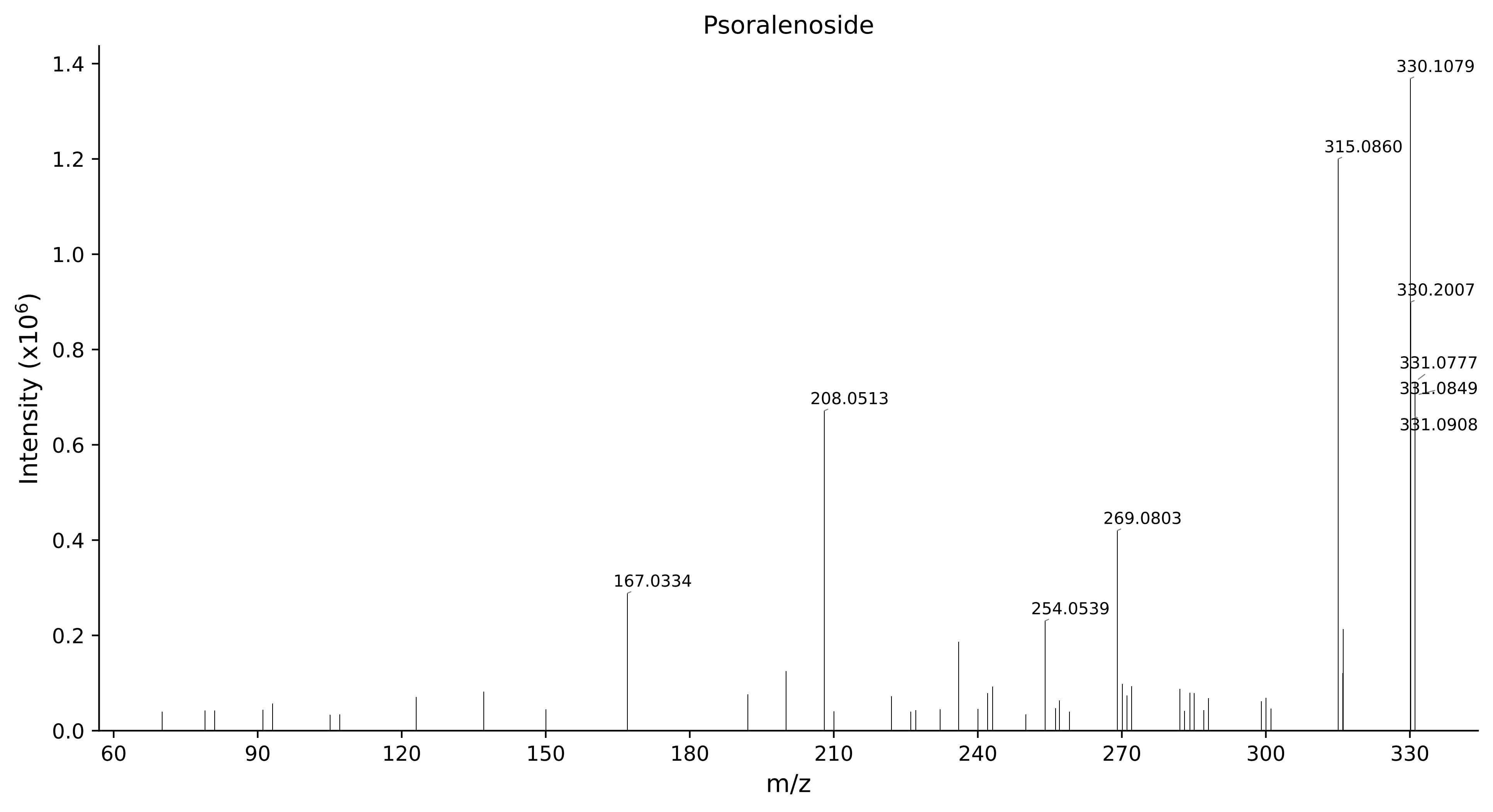
Psoralenoside
Intensity (x10^6)
m/z
330.1079
315.0860
330.2007
331.0777
331.0849
331.0908
208.0513
269.0803
167.0334
254.0539
0.0
0.2
0.4
0.6
0.8
1.0
1.2
1.4
60
90
120
150
180
210
240
270
300
330
