# Supplementary material for: Mafu Yishen Formula ameliorates membranous nephropathy by promotion of regulatory T cell differentiation: a multi-omics study
Source: Chin Med. 2026 Jan 6;21:5. doi: 10.1186/s13020-025-01272-1 (PMC12771757; doi:10.1186/s13020-025-01272-1)
Supplement: Supplementary file 2 — Supplementary material 2. [file 13020_2025_1272_MOESM2_ESM.zip › neg.Cichoric_acid.pdf]

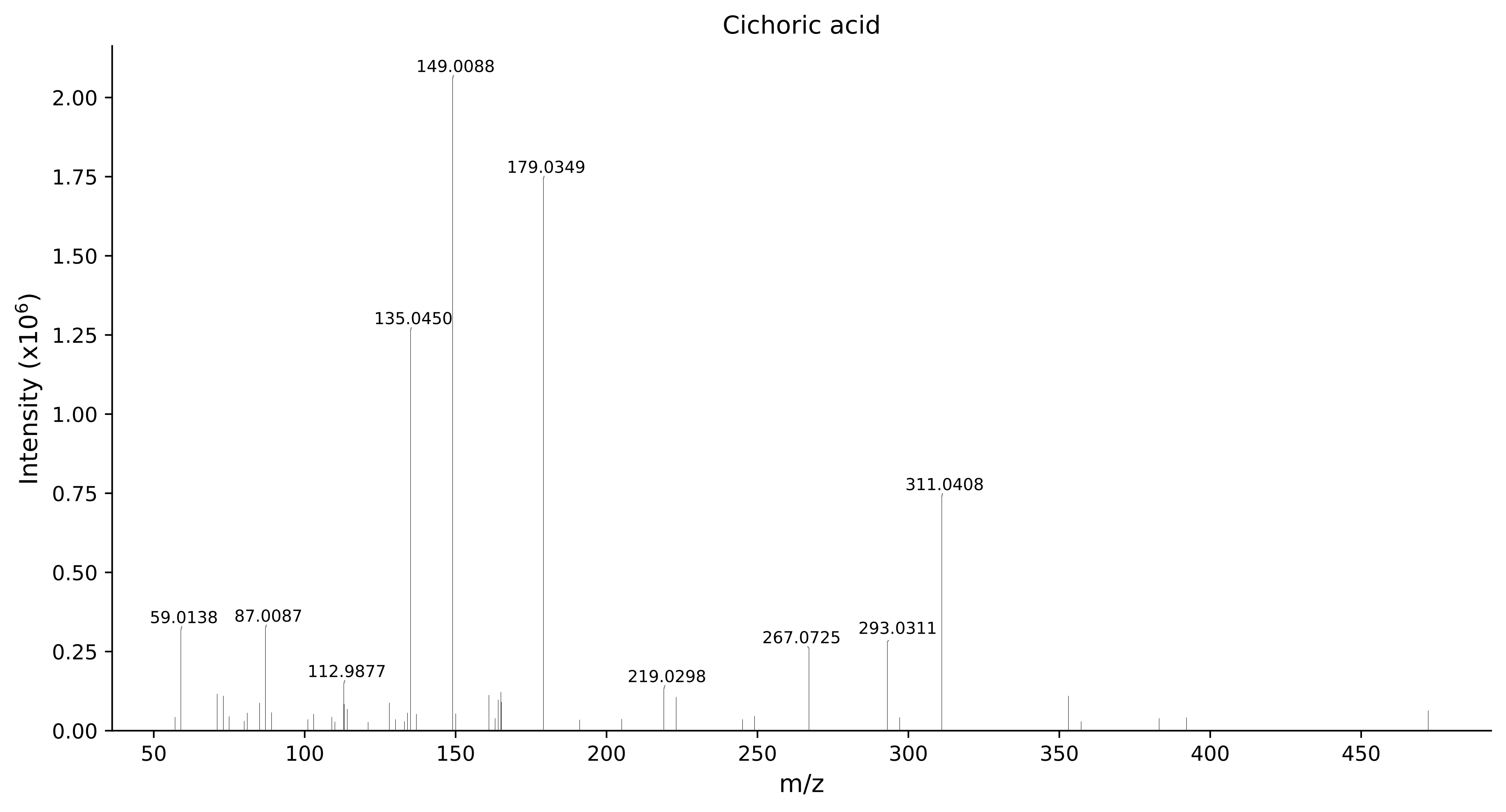
Cichoric acid
Intensity (x10^6)
m/z
149.0088
179.0349
135.0450
311.0408
59.0138
87.0087
112.9877
219.0298
267.0725
293.0311
0.00
0.25
0.50
0.75
1.00
1.25
1.50
1.75
2.00
50
100
150
200
250
300
350
400
450
