# Supplementary material for: Mafu Yishen Formula ameliorates membranous nephropathy by promotion of regulatory T cell differentiation: a multi-omics study
Source: Chin Med. 2026 Jan 6;21:5. doi: 10.1186/s13020-025-01272-1 (PMC12771757; doi:10.1186/s13020-025-01272-1)
Supplement: Supplementary file 2 — Supplementary material 2. [file 13020_2025_1272_MOESM2_ESM.zip › pos.Protostemonine.pdf]

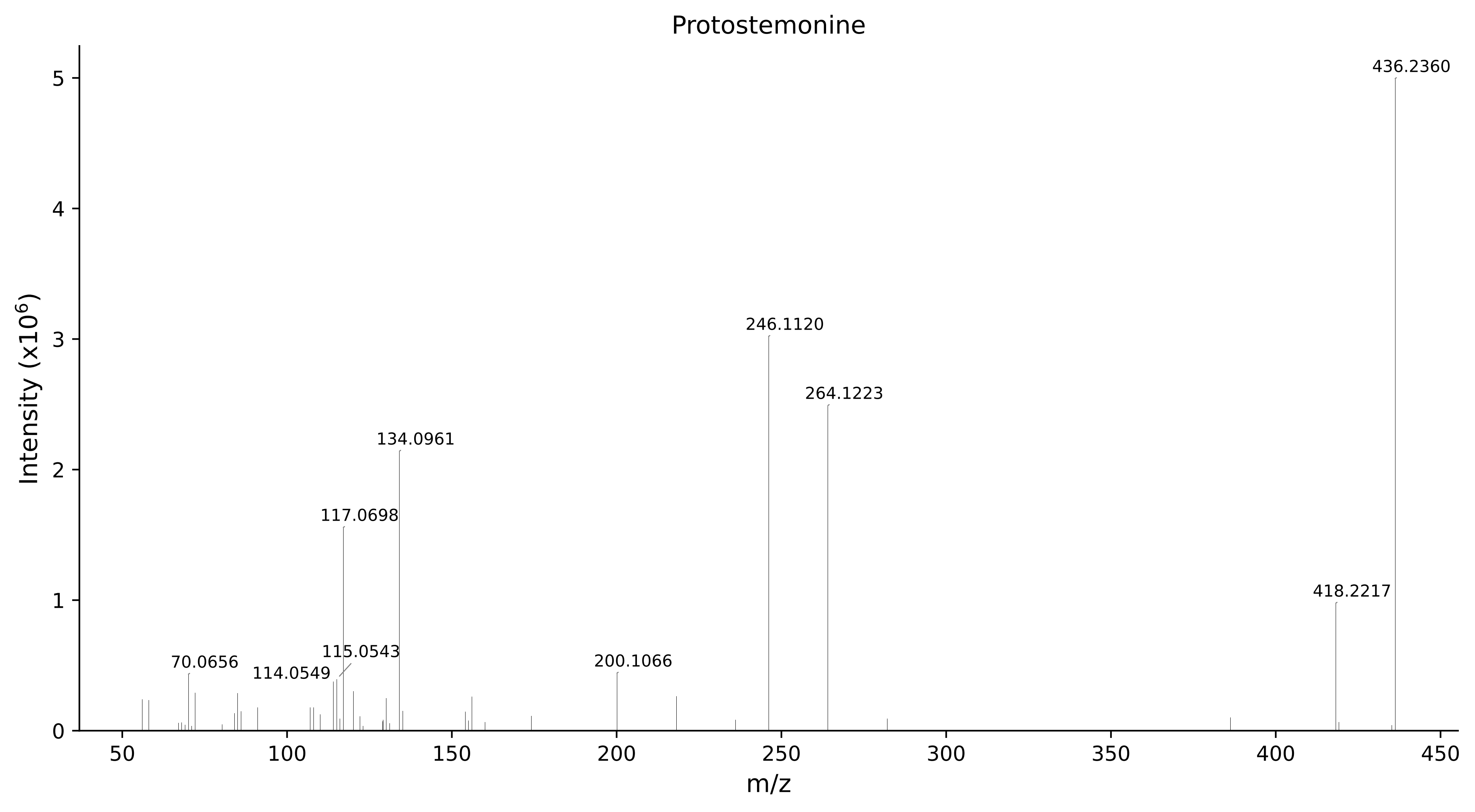

Protostemonine
Intensity (x10^6)
m/z
436.2360
246.1120
264.1223
134.0961
117.0698
418.2217
115.0543
70.0656
114.0549
200.1066
0
1
2
3
4
5
50
100
150
200
250
300
350
400
450
