# Supplementary material for: Mafu Yishen Formula ameliorates membranous nephropathy by promotion of regulatory T cell differentiation: a multi-omics study
Source: Chin Med. 2026 Jan 6;21:5. doi: 10.1186/s13020-025-01272-1 (PMC12771757; doi:10.1186/s13020-025-01272-1)
Supplement: Supplementary file 2 — Supplementary material 2. [file 13020_2025_1272_MOESM2_ESM.zip › neg.16,23-Oxidoalisol_B.pdf]

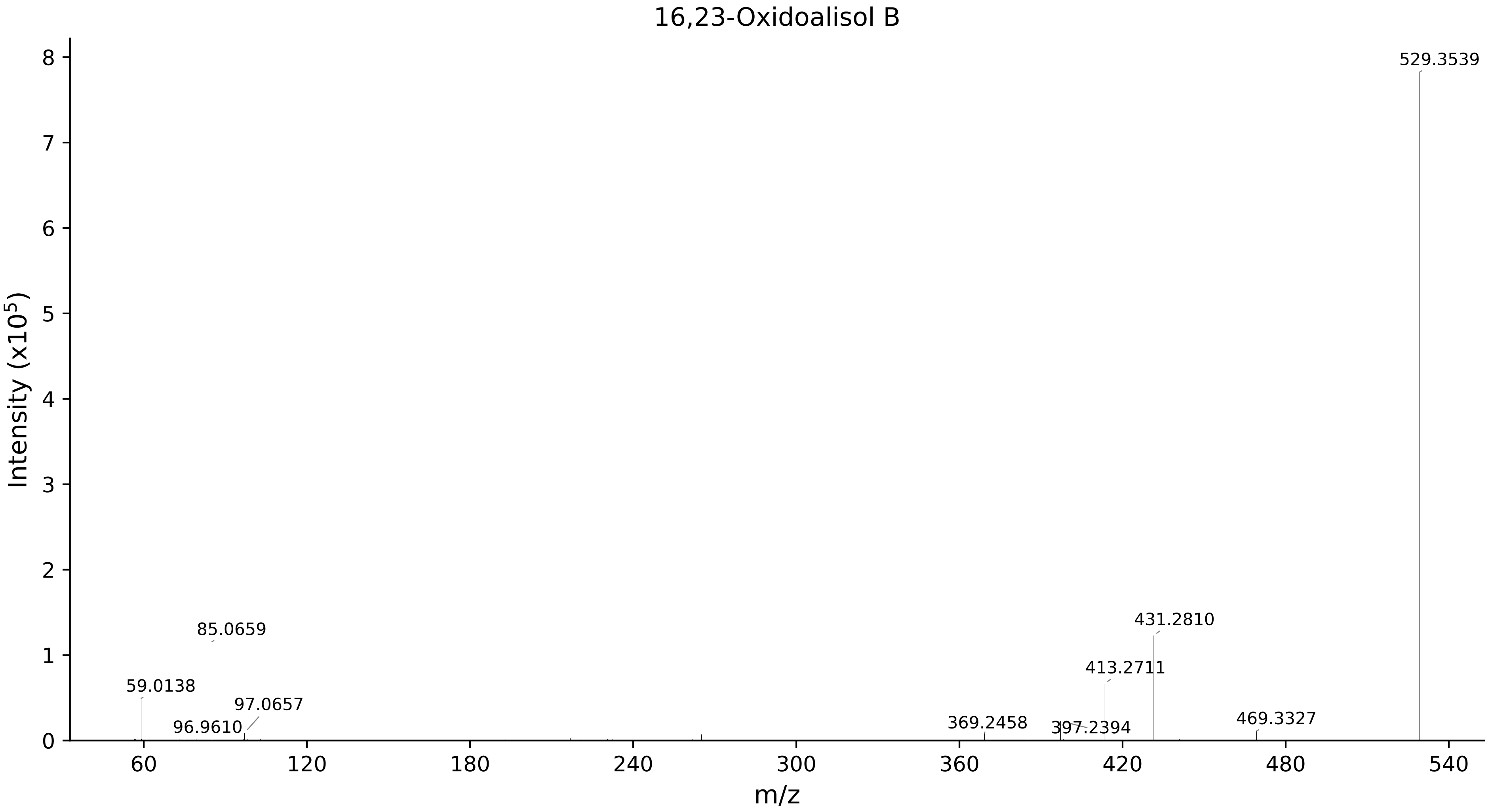
16,23-Oxidoalisol B
Intensity (x10^5)
m/z
529.3539
431.2810
413.2711
469.3327
369.2458
397.2394
85.0659
59.0138
97.0657
96.9610
0
1
2
3
4
5
6
7
8
60
120
180
240
300
360
420
480
540
