# Supplementary material for: Mafu Yishen Formula ameliorates membranous nephropathy by promotion of regulatory T cell differentiation: a multi-omics study
Source: Chin Med. 2026 Jan 6;21:5. doi: 10.1186/s13020-025-01272-1 (PMC12771757; doi:10.1186/s13020-025-01272-1)
Supplement: Supplementary file 2 — Supplementary material 2. [file 13020_2025_1272_MOESM2_ESM.zip › neg.Periandrin_V.pdf]

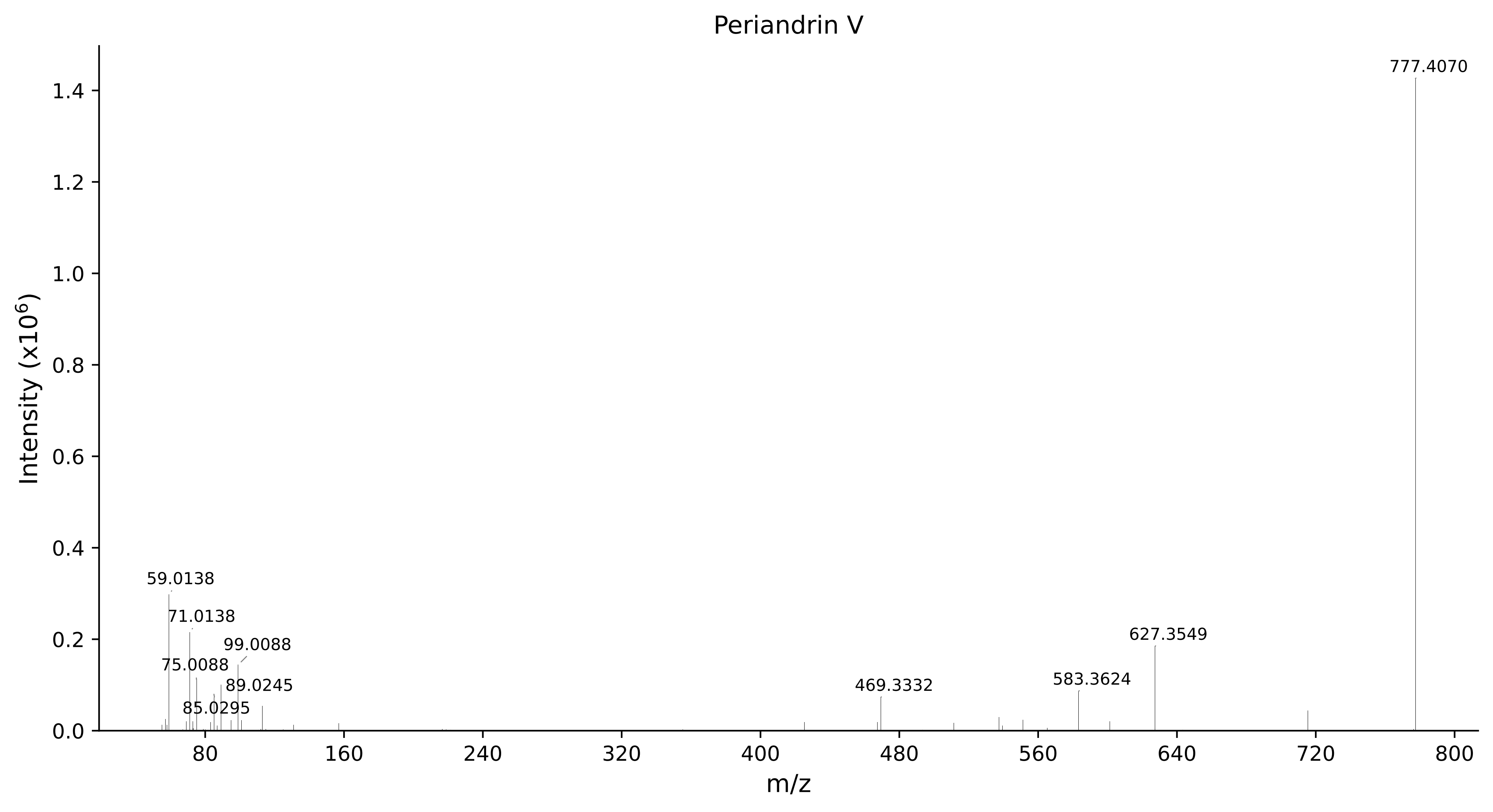

Periandrin V
777.4070
59.0138
71.0138
99.0088
75.0088
89.0245
85.0295
469.3332
583.3624
627.3549
Intensity (x10⁶)
m/z
0.0
0.2
0.4
0.6
0.8
1.0
1.2
1.4
80
160
240
320
400
480
560
640
720
800
