# Supplementary material for: Mafu Yishen Formula ameliorates membranous nephropathy by promotion of regulatory T cell differentiation: a multi-omics study
Source: Chin Med. 2026 Jan 6;21:5. doi: 10.1186/s13020-025-01272-1 (PMC12771757; doi:10.1186/s13020-025-01272-1)
Supplement: Supplementary file 2 — Supplementary material 2. [file 13020_2025_1272_MOESM2_ESM.zip › neg.Saccatoside.pdf]

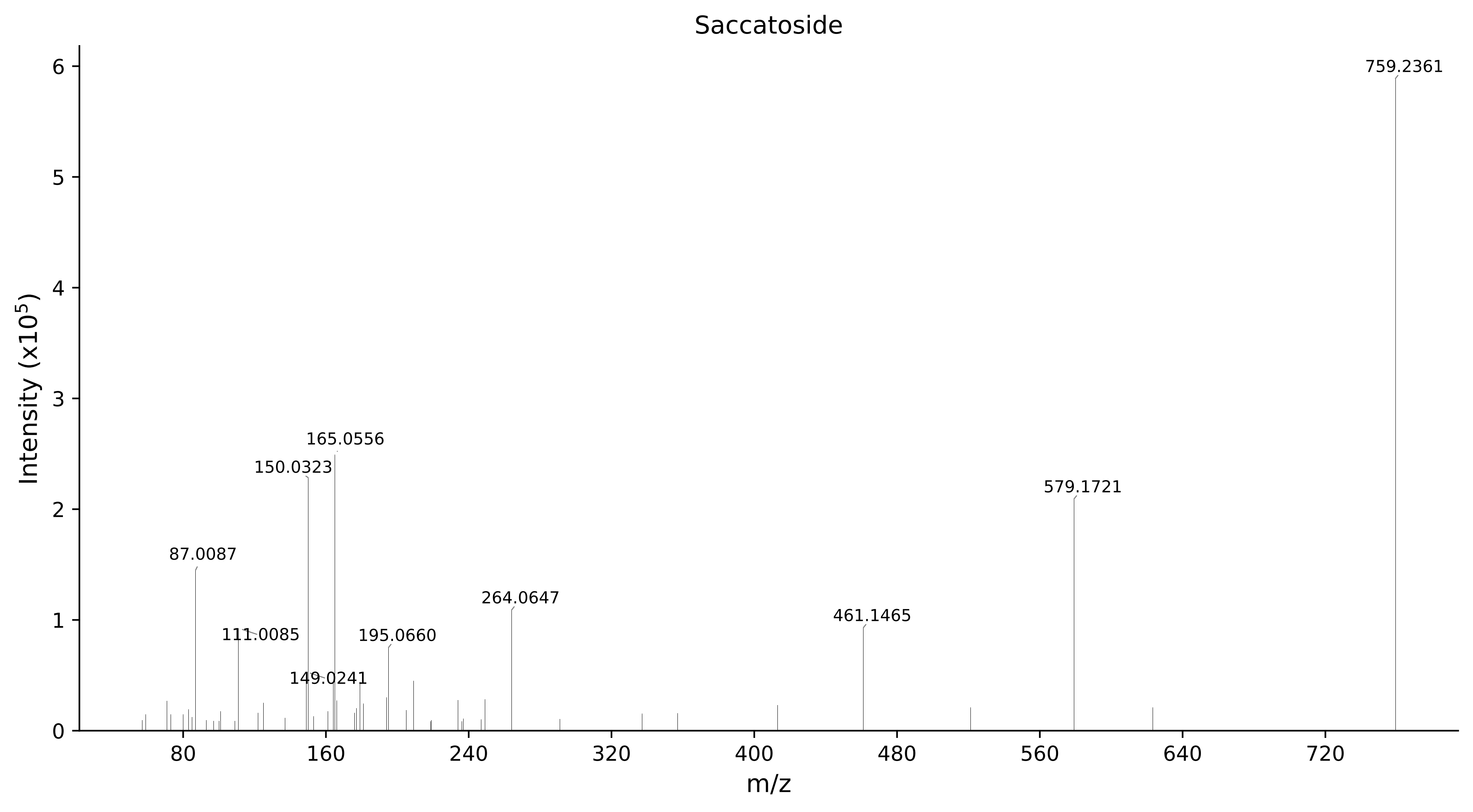

Saccatoside
Intensity (x10^5)
m/z
759.2361
579.1721
165.0556
150.0323
87.0087
264.0647
461.1465
111.0085
195.0660
149.0241
0
1
2
3
4
5
6
80
160
240
320
400
480
560
640
720
