# Supplementary material for: Mafu Yishen Formula ameliorates membranous nephropathy by promotion of regulatory T cell differentiation: a multi-omics study
Source: Chin Med. 2026 Jan 6;21:5. doi: 10.1186/s13020-025-01272-1 (PMC12771757; doi:10.1186/s13020-025-01272-1)
Supplement: Supplementary file 2 — Supplementary material 2. [file 13020_2025_1272_MOESM2_ESM.zip › neg.LysoPC(14_0_0_0).pdf]

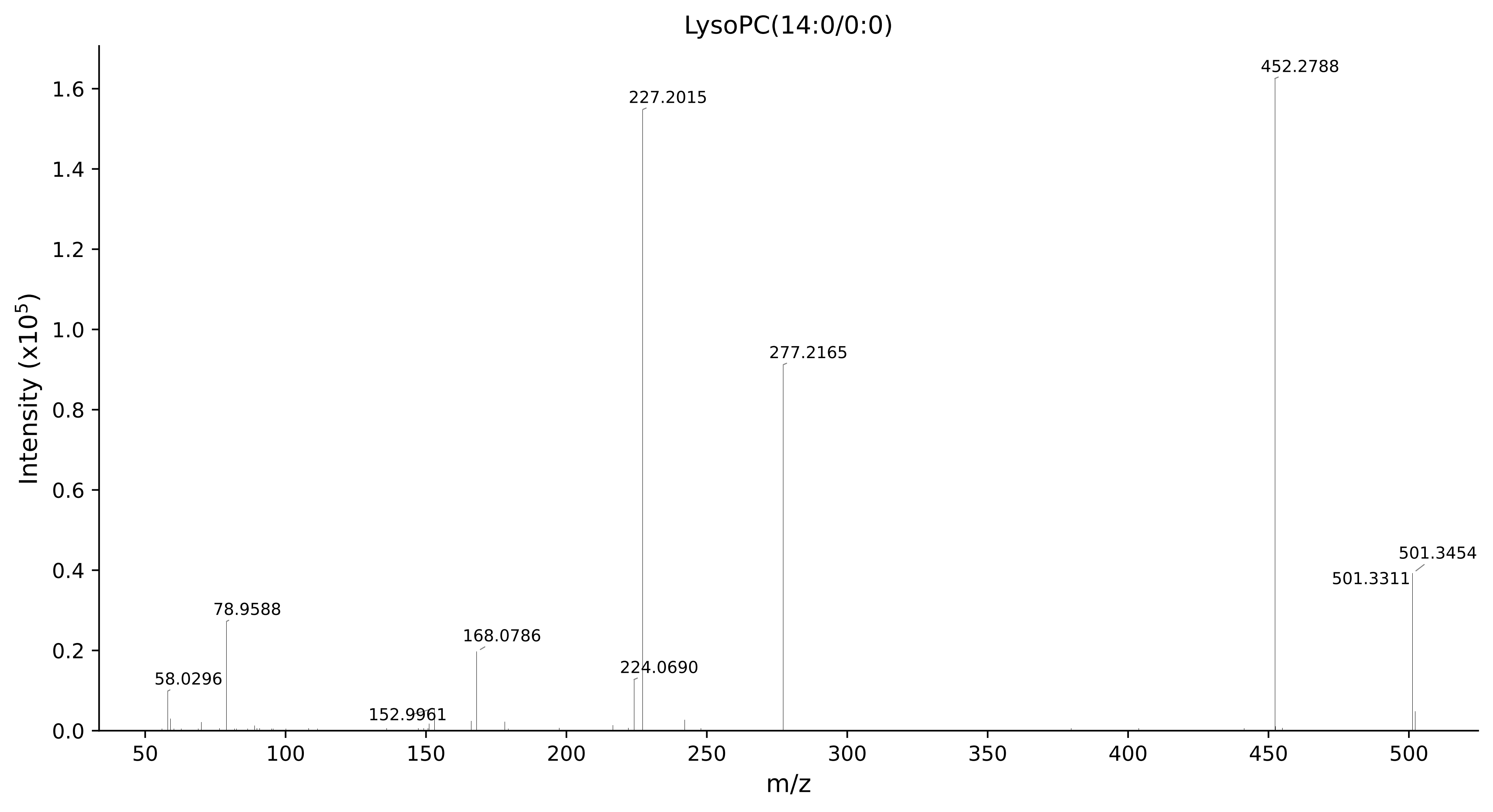

LysoPC(14:0/0:0)
Intensity (x10^5)
m/z
452.2788
227.2015
277.2165
501.3454
501.3311
78.9588
168.0786
224.0690
58.0296
152.9961
1.6
1.4
1.2
1.0
0.8
0.6
0.4
0.2
0.0
50
100
150
200
250
300
350
400
450
500
