# Supplementary material for: Mafu Yishen Formula ameliorates membranous nephropathy by promotion of regulatory T cell differentiation: a multi-omics study
Source: Chin Med. 2026 Jan 6;21:5. doi: 10.1186/s13020-025-01272-1 (PMC12771757; doi:10.1186/s13020-025-01272-1)
Supplement: Supplementary file 2 — Supplementary material 2. [file 13020_2025_1272_MOESM2_ESM.zip › pos.Alangionoside_A.pdf]

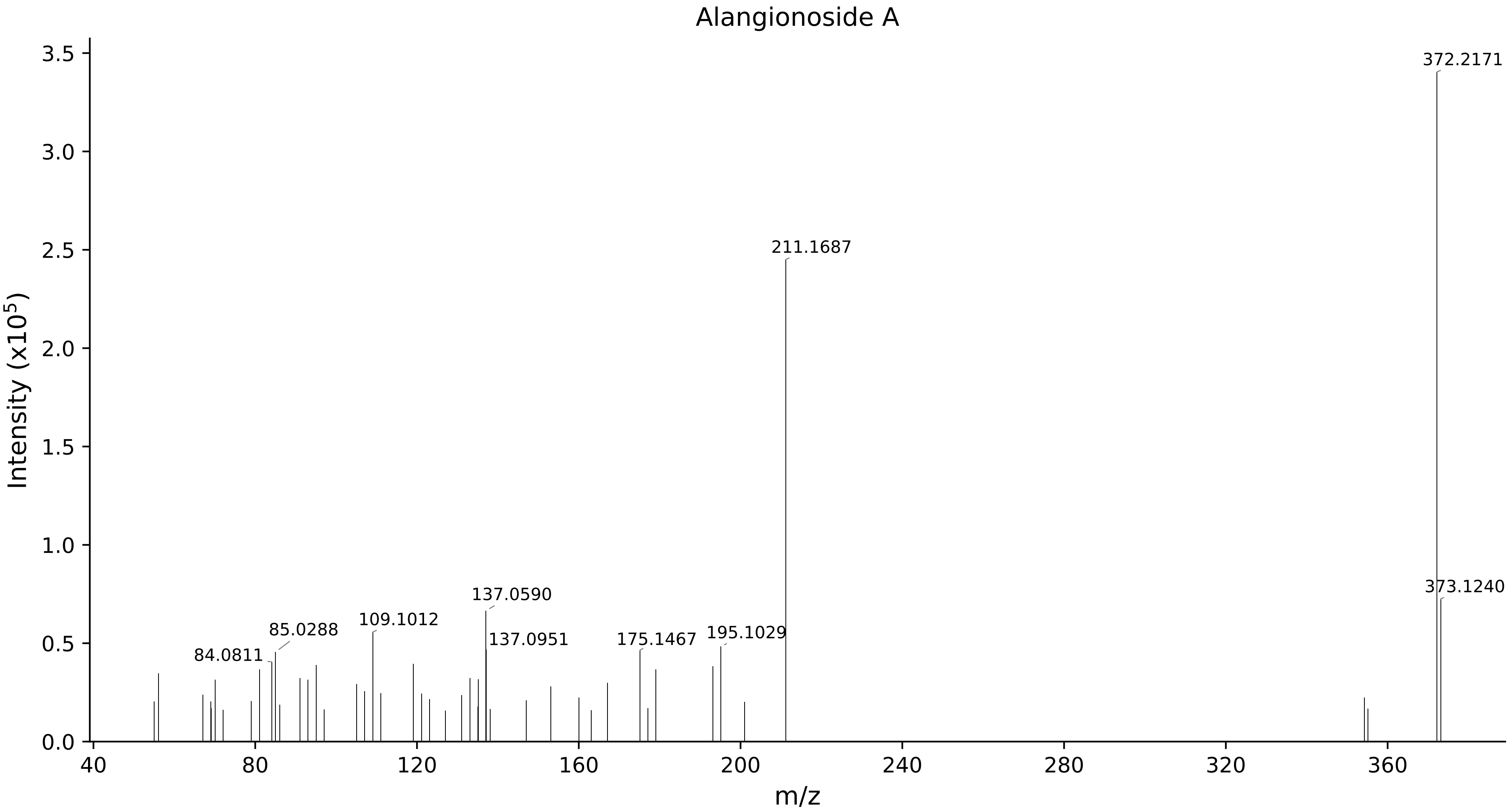

Alangionoside A
Intensity (x10^5)
m/z
372.2171
211.1687
373.1240
137.0590
109.1012
85.0288
84.0811
137.0951
175.1467
195.1029
0.0
0.5
1.0
1.5
2.0
2.5
3.0
3.5
40
80
120
160
200
240
280
320
360
