# Supplementary material for: Mafu Yishen Formula ameliorates membranous nephropathy by promotion of regulatory T cell differentiation: a multi-omics study
Source: Chin Med. 2026 Jan 6;21:5. doi: 10.1186/s13020-025-01272-1 (PMC12771757; doi:10.1186/s13020-025-01272-1)
Supplement: Supplementary file 2 — Supplementary material 2. [file 13020_2025_1272_MOESM2_ESM.zip › pos.ent-11alpha,16alpha-Epoxy-15alpha-hydroxykauran-19-oic_acid.pdf]

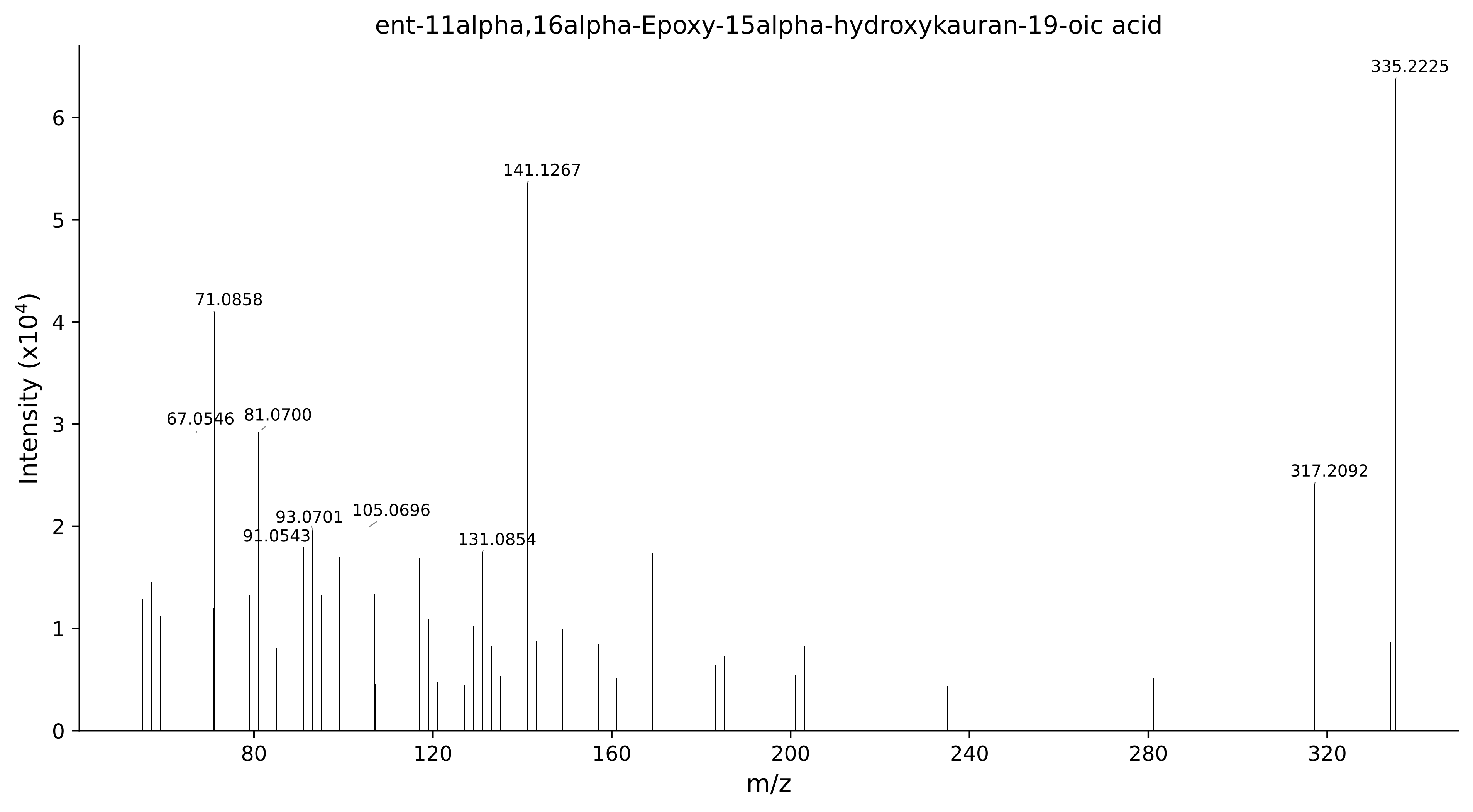
ent-11alpha,16alpha-Epoxy-15alpha-hydroxykauran-19-oic acid
Intensity (x10^4)
m/z
0
1
2
3
4
5
6
80
120
160
200
240
280
320
335.2225
141.1267
71.0858
67.0546
81.0700
93.0701
91.0543
105.0696
131.0854
317.2092
