# Supplementary material for: Mafu Yishen Formula ameliorates membranous nephropathy by promotion of regulatory T cell differentiation: a multi-omics study
Source: Chin Med. 2026 Jan 6;21:5. doi: 10.1186/s13020-025-01272-1 (PMC12771757; doi:10.1186/s13020-025-01272-1)
Supplement: Supplementary file 2 — Supplementary material 2. [file 13020_2025_1272_MOESM2_ESM.zip › pos.Gardoside.pdf]

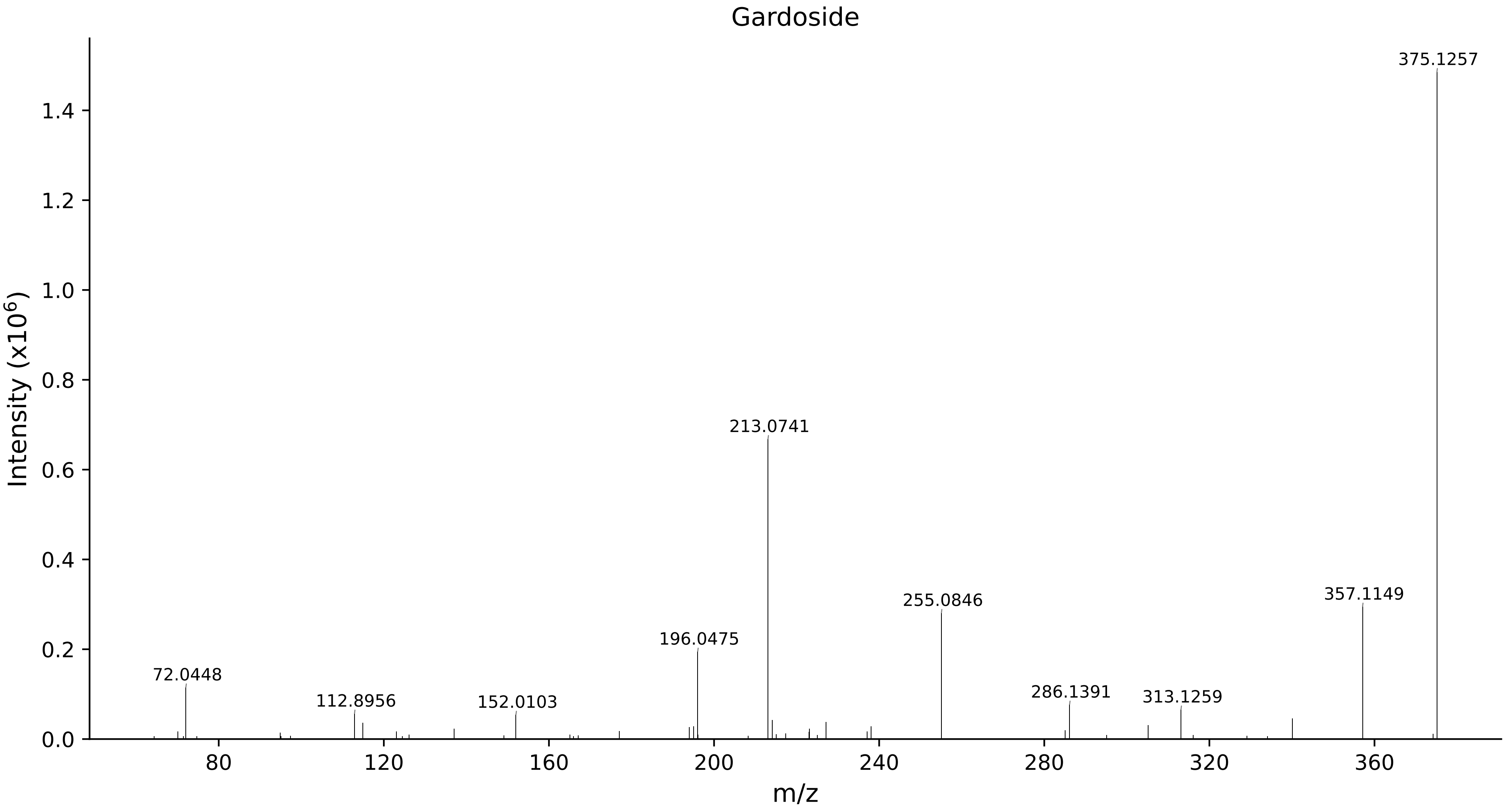
Gardoside
Intensity (x10^6)
m/z
0.0
0.2
0.4
0.6
0.8
1.0
1.2
1.4
80
120
160
200
240
280
320
360
72.0448
112.8956
152.0103
196.0475
213.0741
255.0846
286.1391
313.1259
357.1149
375.1257
