# Supplementary material for: Mafu Yishen Formula ameliorates membranous nephropathy by promotion of regulatory T cell differentiation: a multi-omics study
Source: Chin Med. 2026 Jan 6;21:5. doi: 10.1186/s13020-025-01272-1 (PMC12771757; doi:10.1186/s13020-025-01272-1)
Supplement: Supplementary file 2 — Supplementary material 2. [file 13020_2025_1272_MOESM2_ESM.zip › neg.2-Ethylbutyric_Acid.pdf]

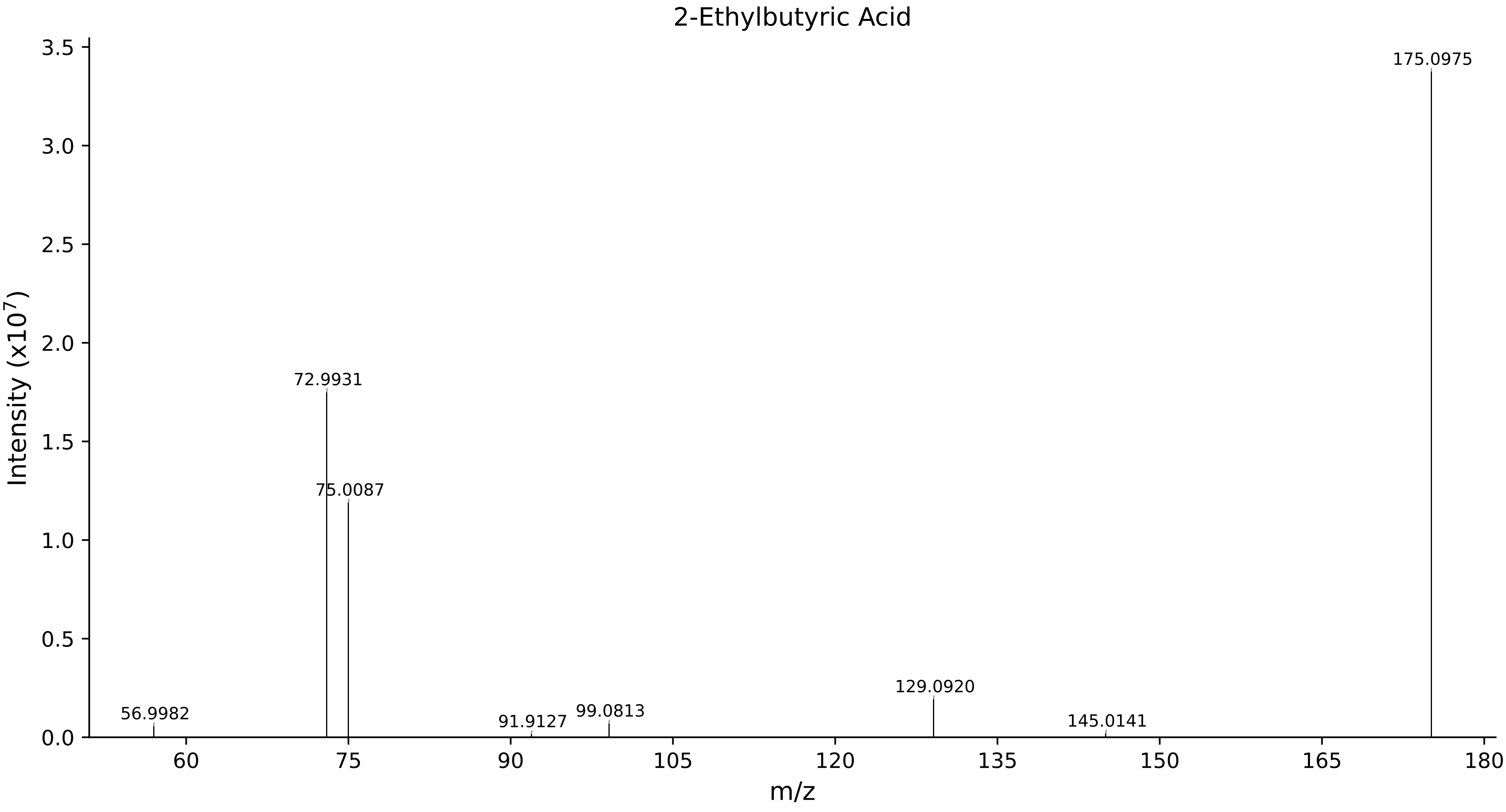

2-Ethylbutyric Acid
Intensity (x10^7)
m/z
3.5
3.0
2.5
2.0
1.5
1.0
0.5
0.0
60
75
90
105
120
135
150
165
180
175.0975
72.9931
75.0087
129.0920
56.9982
91.9127
99.0813
145.0141
