# Supplementary material for: Mafu Yishen Formula ameliorates membranous nephropathy by promotion of regulatory T cell differentiation: a multi-omics study
Source: Chin Med. 2026 Jan 6;21:5. doi: 10.1186/s13020-025-01272-1 (PMC12771757; doi:10.1186/s13020-025-01272-1)
Supplement: Supplementary file 2 — Supplementary material 2. [file 13020_2025_1272_MOESM2_ESM.zip › neg.Curvulinic_acid.pdf]

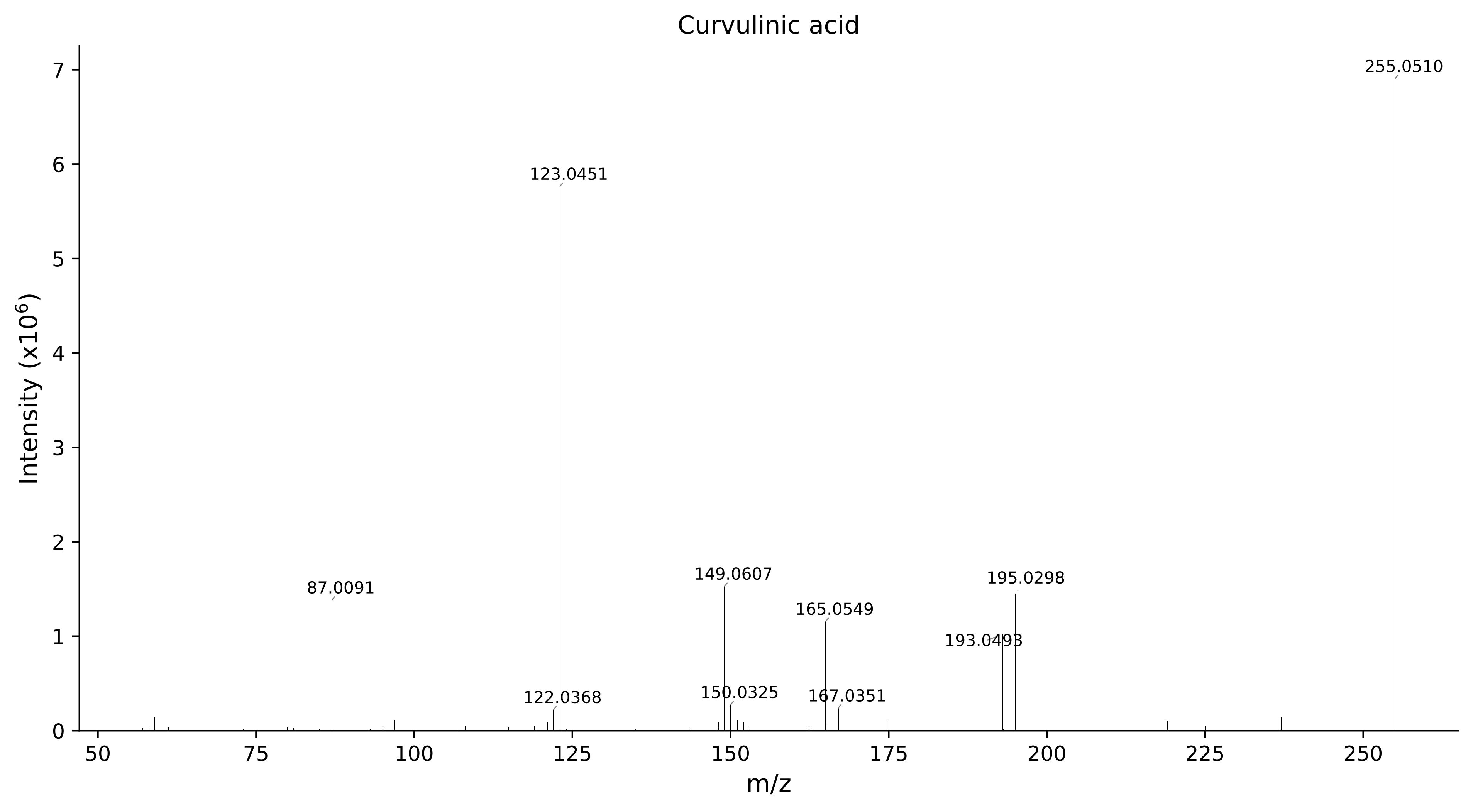
Curvulinic acid
Intensity (x10^6)
m/z
255.0510
123.0451
87.0091
149.0607
195.0298
165.0549
193.0493
122.0368
150.0325
167.0351
0
1
2
3
4
5
6
7
50
75
100
125
150
175
200
225
250
