# Supplementary material for: Mafu Yishen Formula ameliorates membranous nephropathy by promotion of regulatory T cell differentiation: a multi-omics study
Source: Chin Med. 2026 Jan 6;21:5. doi: 10.1186/s13020-025-01272-1 (PMC12771757; doi:10.1186/s13020-025-01272-1)
Supplement: Supplementary file 2 — Supplementary material 2. [file 13020_2025_1272_MOESM2_ESM.zip › neg.Rivulobirin_E.pdf]

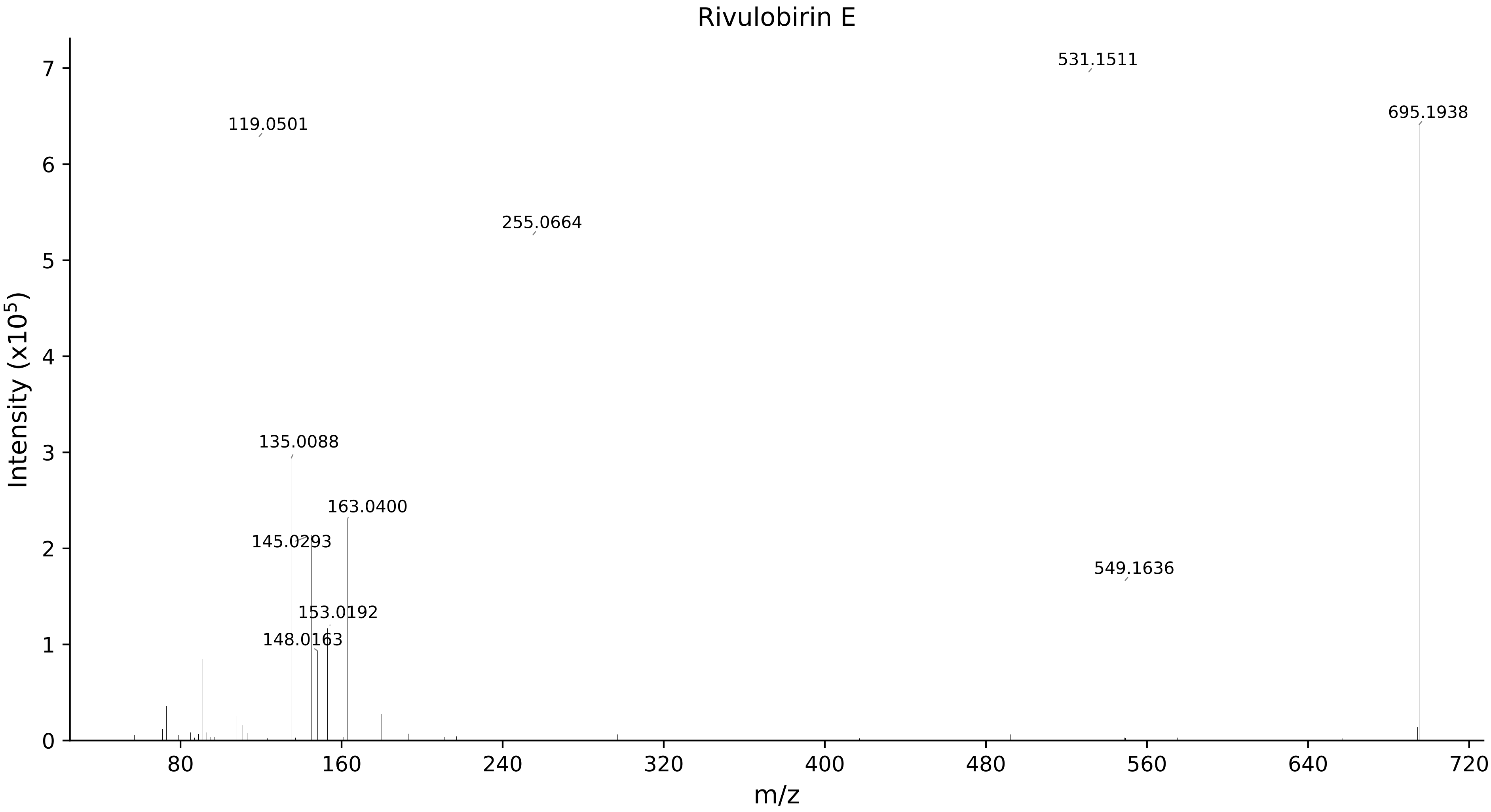

Rivulobirin E
531.1511
695.1938
119.0501
255.0664
135.0088
163.0400
145.0293
549.1636
153.0192
148.0163
Intensity (x10^5)
0
1
2
3
4
5
6
7
80
160
240
320
400
480
560
640
720
m/z
