# Supplementary material for: Mafu Yishen Formula ameliorates membranous nephropathy by promotion of regulatory T cell differentiation: a multi-omics study
Source: Chin Med. 2026 Jan 6;21:5. doi: 10.1186/s13020-025-01272-1 (PMC12771757; doi:10.1186/s13020-025-01272-1)
Supplement: Supplementary file 2 — Supplementary material 2. [file 13020_2025_1272_MOESM2_ESM.zip › neg.Compound_2.pdf]

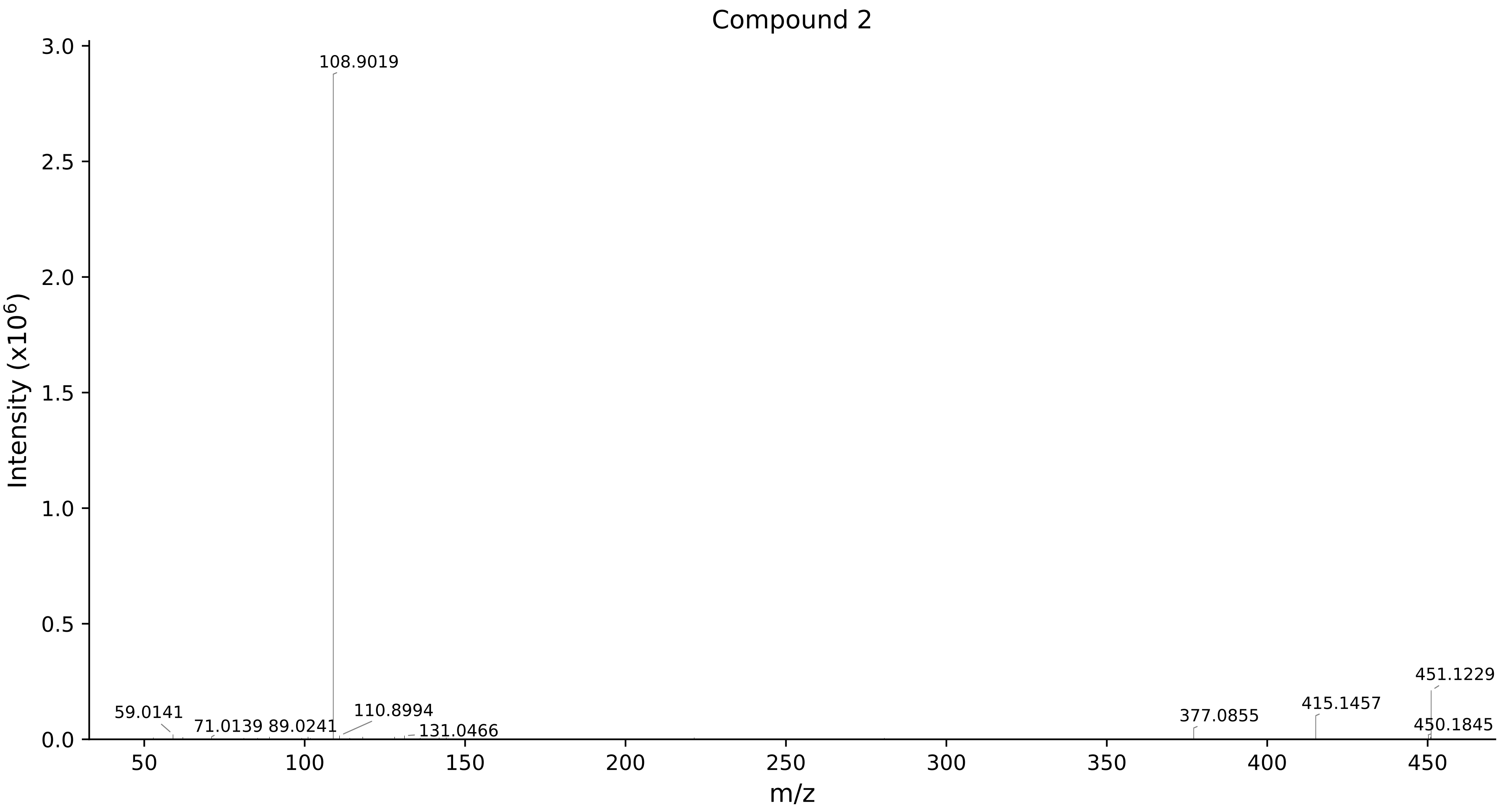
Compound 2
Intensity (x10^6)
m/z
108.9019
59.0141
71.0139
89.0241
110.8994
131.0466
377.0855
415.1457
451.1229
450.1845
0.0
0.5
1.0
1.5
2.0
2.5
3.0
50
100
150
200
250
300
350
400
450
