# Supplementary material for: Mafu Yishen Formula ameliorates membranous nephropathy by promotion of regulatory T cell differentiation: a multi-omics study
Source: Chin Med. 2026 Jan 6;21:5. doi: 10.1186/s13020-025-01272-1 (PMC12771757; doi:10.1186/s13020-025-01272-1)
Supplement: Supplementary file 2 — Supplementary material 2. [file 13020_2025_1272_MOESM2_ESM.zip › pos.Bixin.pdf]

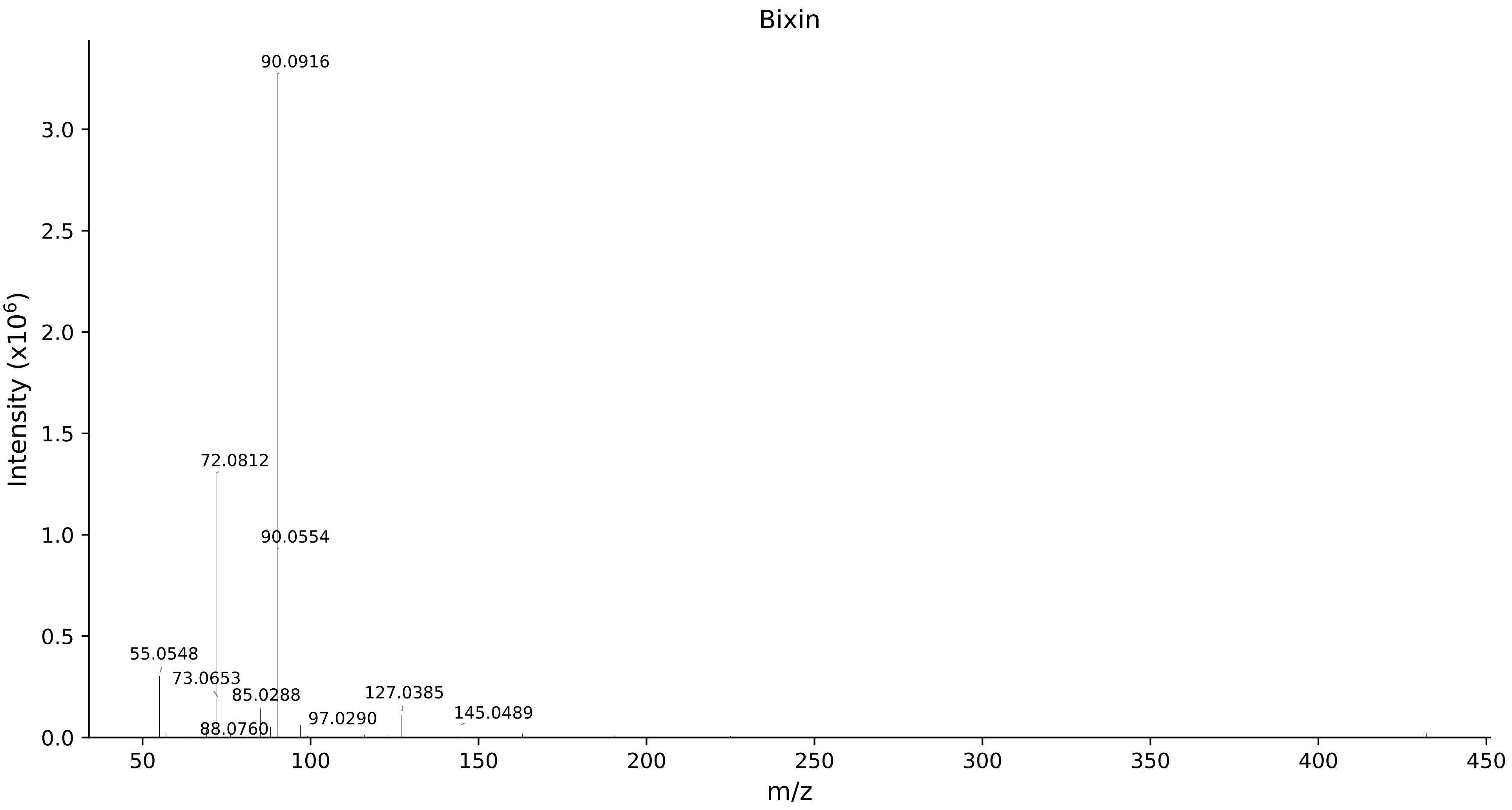
Bixin
90.0916
72.0812
90.0554
55.0548
73.0653
85.0288
127.0385
145.0489
97.0290
88.0760
Intensity (x10^6)
3.0
2.5
2.0
1.5
1.0
0.5
0.0
50
100
150
200
250
300
350
400
450
m/z
