# Supplementary material for: Mafu Yishen Formula ameliorates membranous nephropathy by promotion of regulatory T cell differentiation: a multi-omics study
Source: Chin Med. 2026 Jan 6;21:5. doi: 10.1186/s13020-025-01272-1 (PMC12771757; doi:10.1186/s13020-025-01272-1)
Supplement: Supplementary file 2 — Supplementary material 2. [file 13020_2025_1272_MOESM2_ESM.zip › neg.Lactobionic_acid.pdf]

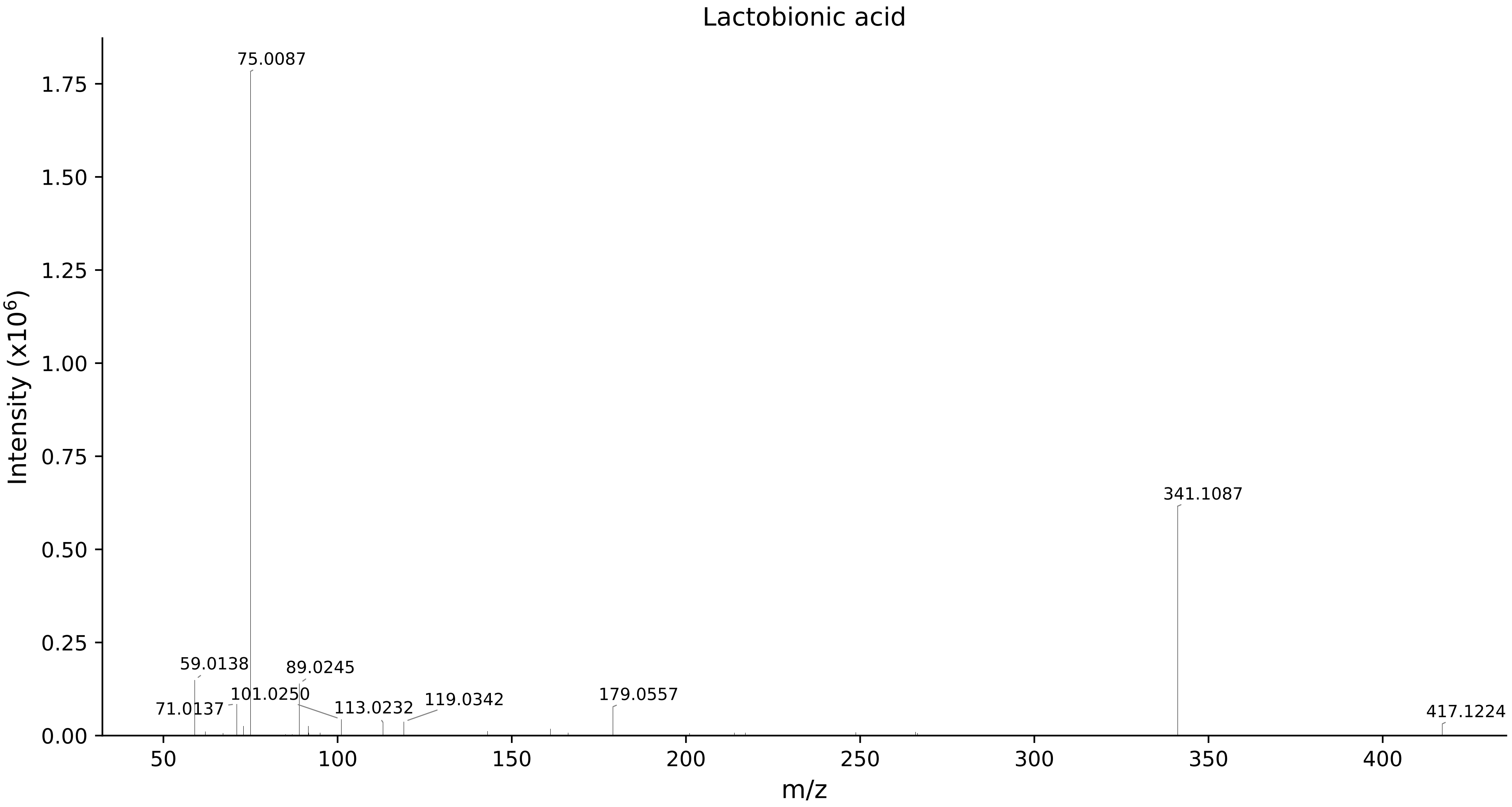

Lactobionic acid
Intensity (x10^6)
1.75
1.50
1.25
1.00
0.75
0.50
0.25
0.00
75.0087
341.1087
59.0138
89.0245
101.0250
71.0137
113.0232
119.0342
179.0557
417.1224
50
100
150
200
250
300
350
400
m/z
