# Supplementary material for: Mafu Yishen Formula ameliorates membranous nephropathy by promotion of regulatory T cell differentiation: a multi-omics study
Source: Chin Med. 2026 Jan 6;21:5. doi: 10.1186/s13020-025-01272-1 (PMC12771757; doi:10.1186/s13020-025-01272-1)
Supplement: Supplementary file 2 — Supplementary material 2. [file 13020_2025_1272_MOESM2_ESM.zip › neg.7-Acetoxy-15-methoxy-10-O-methyldihydrobotrydial.pdf]

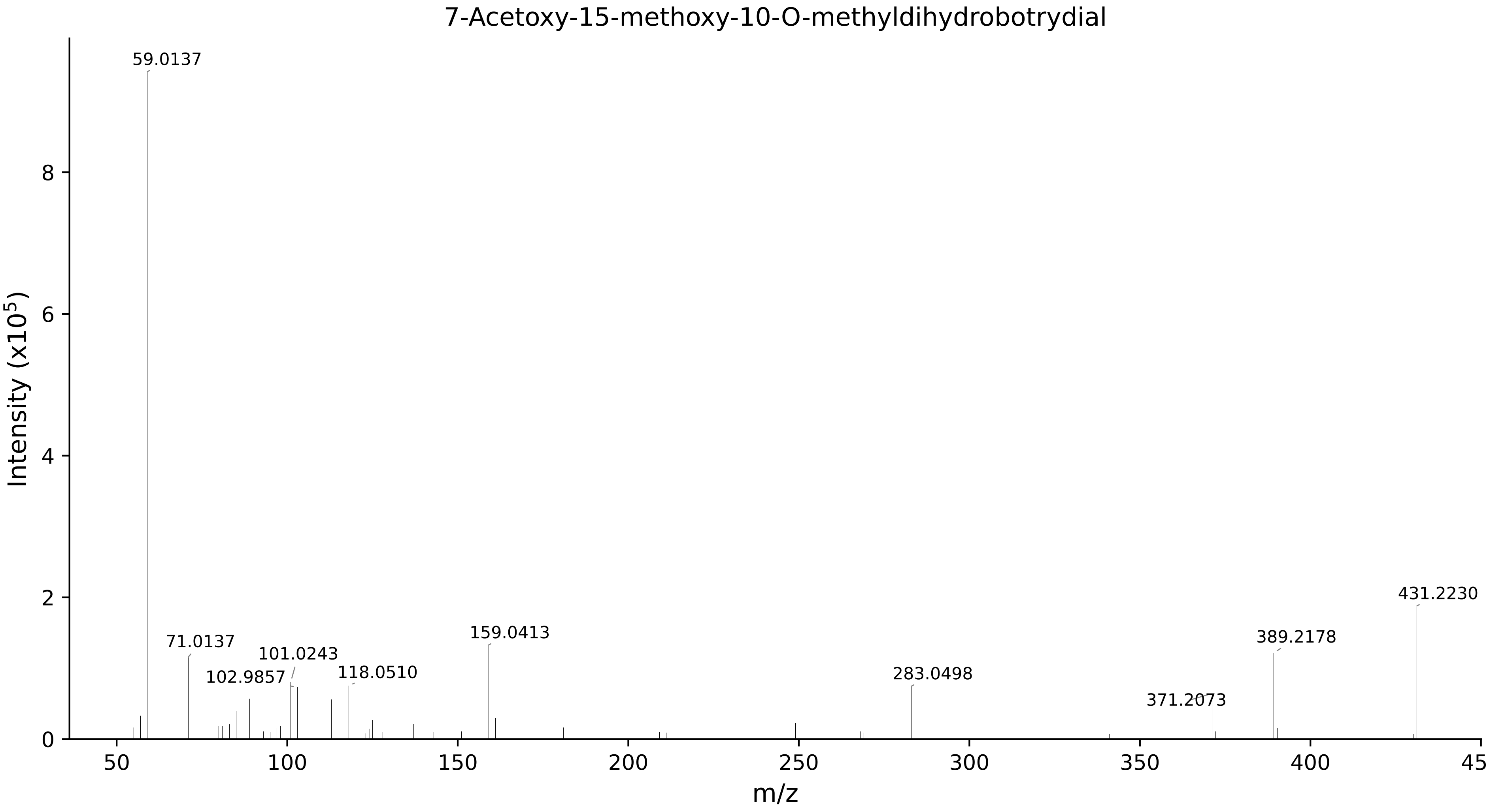

7-Acetoxy-15-methoxy-10-O-methyldihydrobotrydial
59.0137
71.0137
102.9857
101.0243
118.0510
159.0413
283.0498
371.2073
389.2178
431.2230
Intensity (x10^5)
0
2
4
6
8
m/z
50
100
150
200
250
300
350
400
450
