# Supplementary material for: Mafu Yishen Formula ameliorates membranous nephropathy by promotion of regulatory T cell differentiation: a multi-omics study
Source: Chin Med. 2026 Jan 6;21:5. doi: 10.1186/s13020-025-01272-1 (PMC12771757; doi:10.1186/s13020-025-01272-1)
Supplement: Supplementary file 2 — Supplementary material 2. [file 13020_2025_1272_MOESM2_ESM.zip › neg.Resveratrol.pdf]

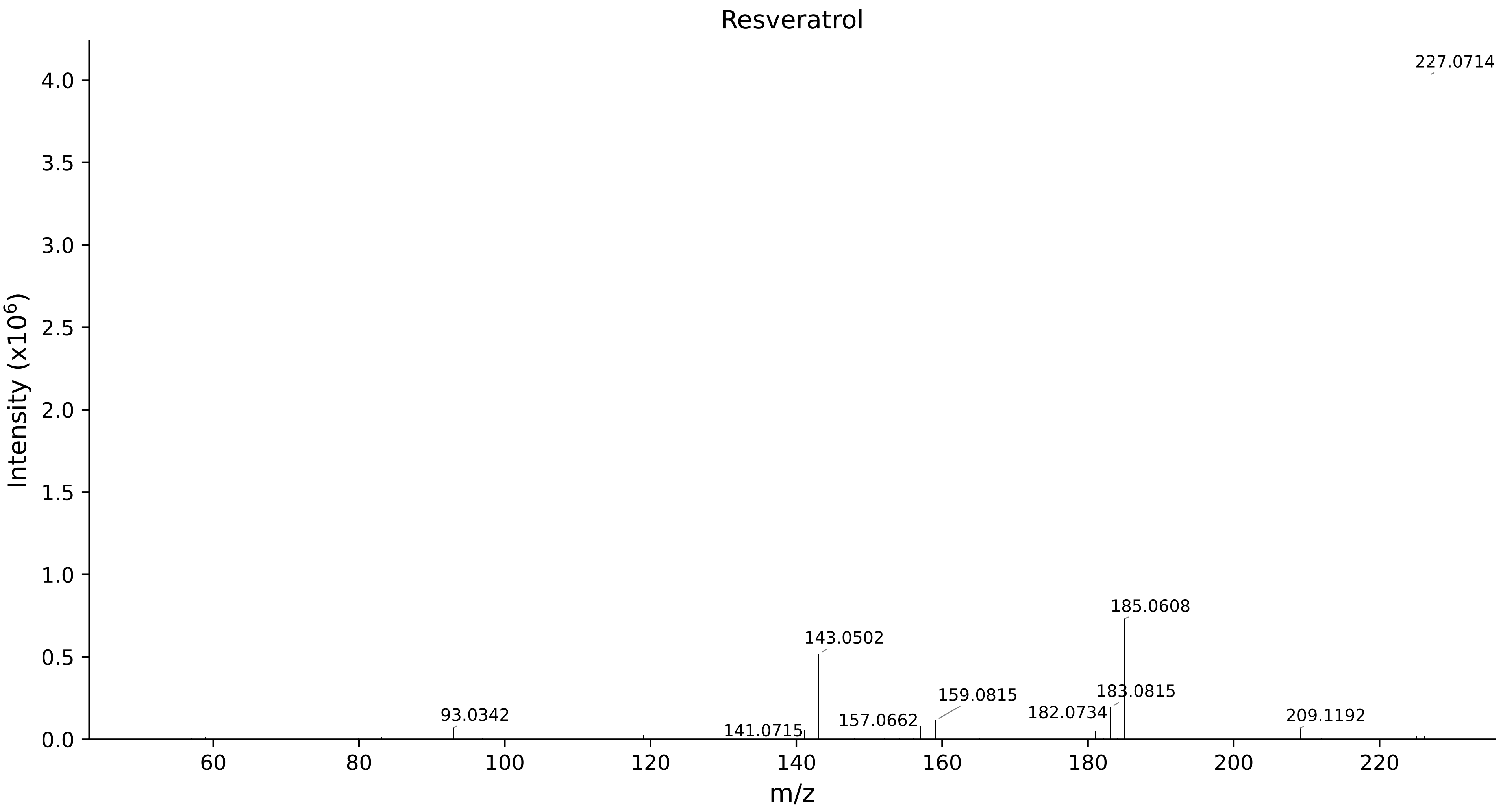
Resveratrol
227.0714
185.0608
143.0502
159.0815
183.0815
182.0734
157.0662
93.0342
209.1192
141.0715
Intensity (x10^6)
4.0
3.5
3.0
2.5
2.0
1.5
1.0
0.5
0.0
60
80
100
120
140
160
180
200
220
m/z
