# Supplementary material for: Mafu Yishen Formula ameliorates membranous nephropathy by promotion of regulatory T cell differentiation: a multi-omics study
Source: Chin Med. 2026 Jan 6;21:5. doi: 10.1186/s13020-025-01272-1 (PMC12771757; doi:10.1186/s13020-025-01272-1)
Supplement: Supplementary file 2 — Supplementary material 2. [file 13020_2025_1272_MOESM2_ESM.zip › pos.1-Hydroxy-2-oxopomolic_acid.pdf]

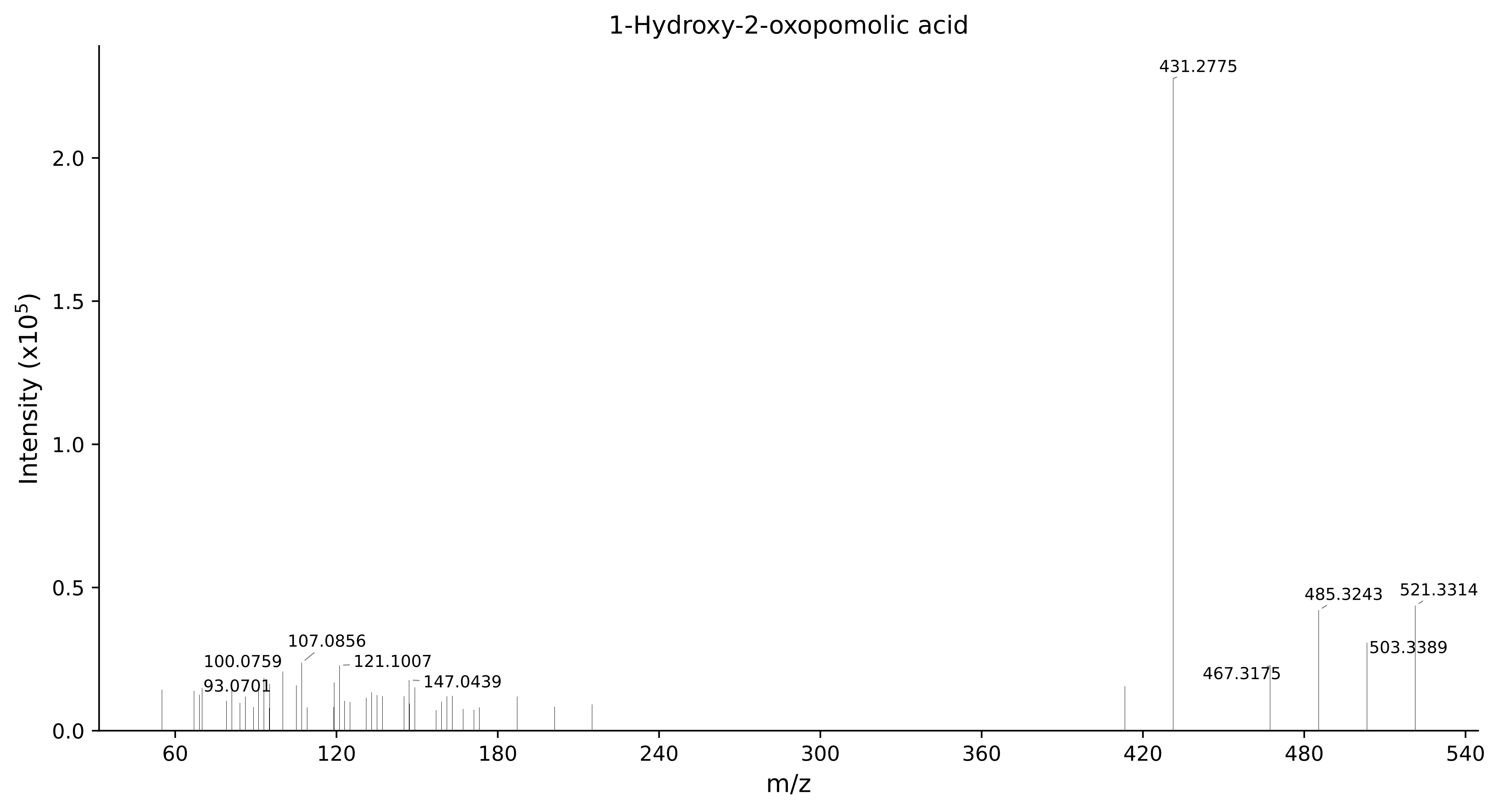

1-Hydroxy-2-oxopomolic acid
Intensity (x10^5)
m/z
2.0
1.5
1.0
0.5
0.0
60
120
180
240
300
360
420
480
540
431.2775
485.3243
521.3314
503.3389
467.3175
107.0856
100.0759
121.1007
147.0439
93.0701
