# Supplementary material for: Mafu Yishen Formula ameliorates membranous nephropathy by promotion of regulatory T cell differentiation: a multi-omics study
Source: Chin Med. 2026 Jan 6;21:5. doi: 10.1186/s13020-025-01272-1 (PMC12771757; doi:10.1186/s13020-025-01272-1)
Supplement: Supplementary file 2 — Supplementary material 2. [file 13020_2025_1272_MOESM2_ESM.zip › neg.Boschnaloside.pdf]

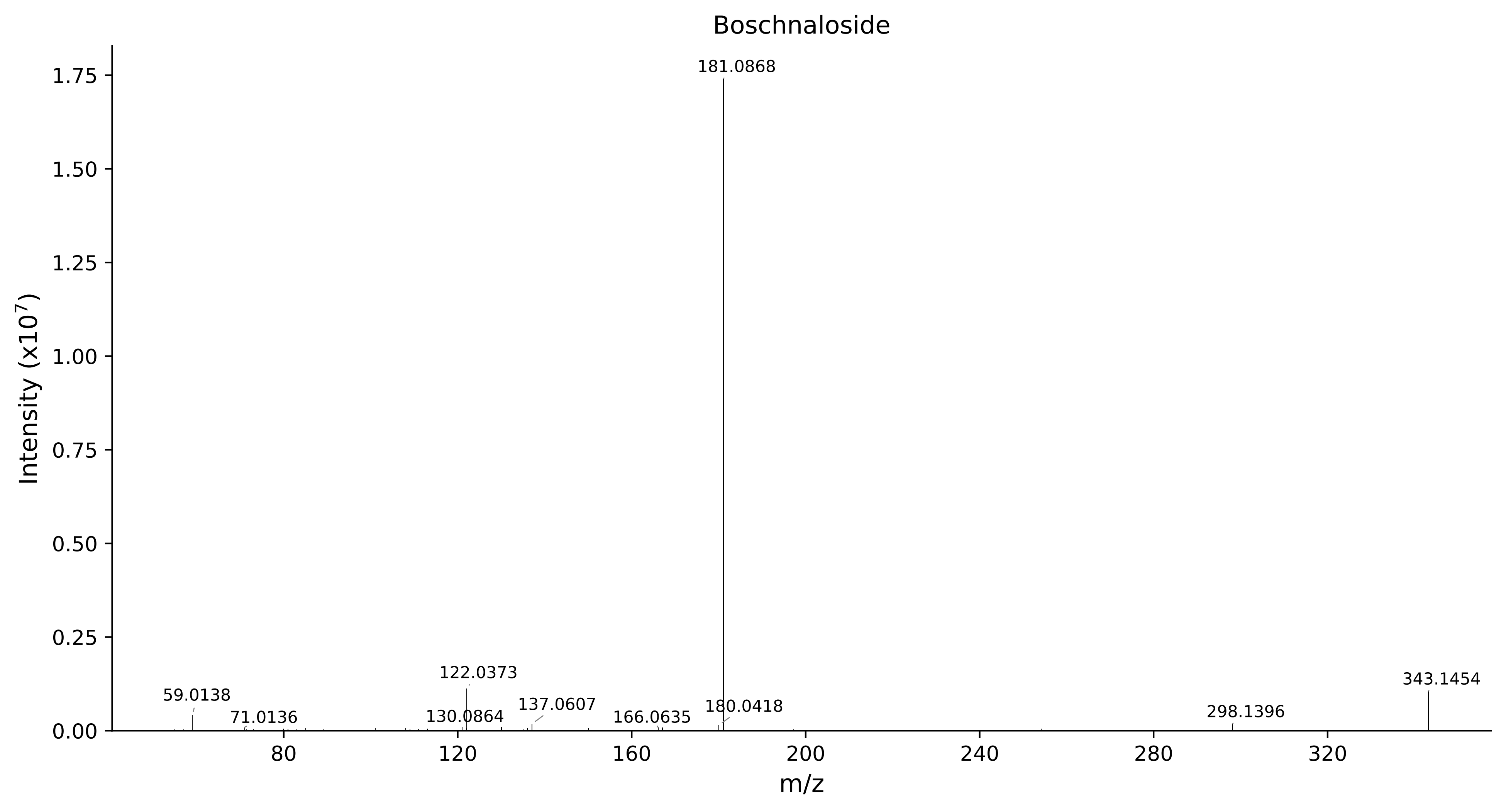

Boschnaloside
Intensity (x10^{7})
1.75
1.50
1.25
1.00
0.75
0.50
0.25
0.00
181.0868
122.0373
59.0138
71.0136
130.0864
137.0607
166.0635
180.0418
298.1396
343.1454
80
120
160
200
240
280
320
m/z
