# Supplementary material for: Mafu Yishen Formula ameliorates membranous nephropathy by promotion of regulatory T cell differentiation: a multi-omics study
Source: Chin Med. 2026 Jan 6;21:5. doi: 10.1186/s13020-025-01272-1 (PMC12771757; doi:10.1186/s13020-025-01272-1)
Supplement: Supplementary file 2 — Supplementary material 2. [file 13020_2025_1272_MOESM2_ESM.zip › pos.Alisol_B_23-acetate.pdf]

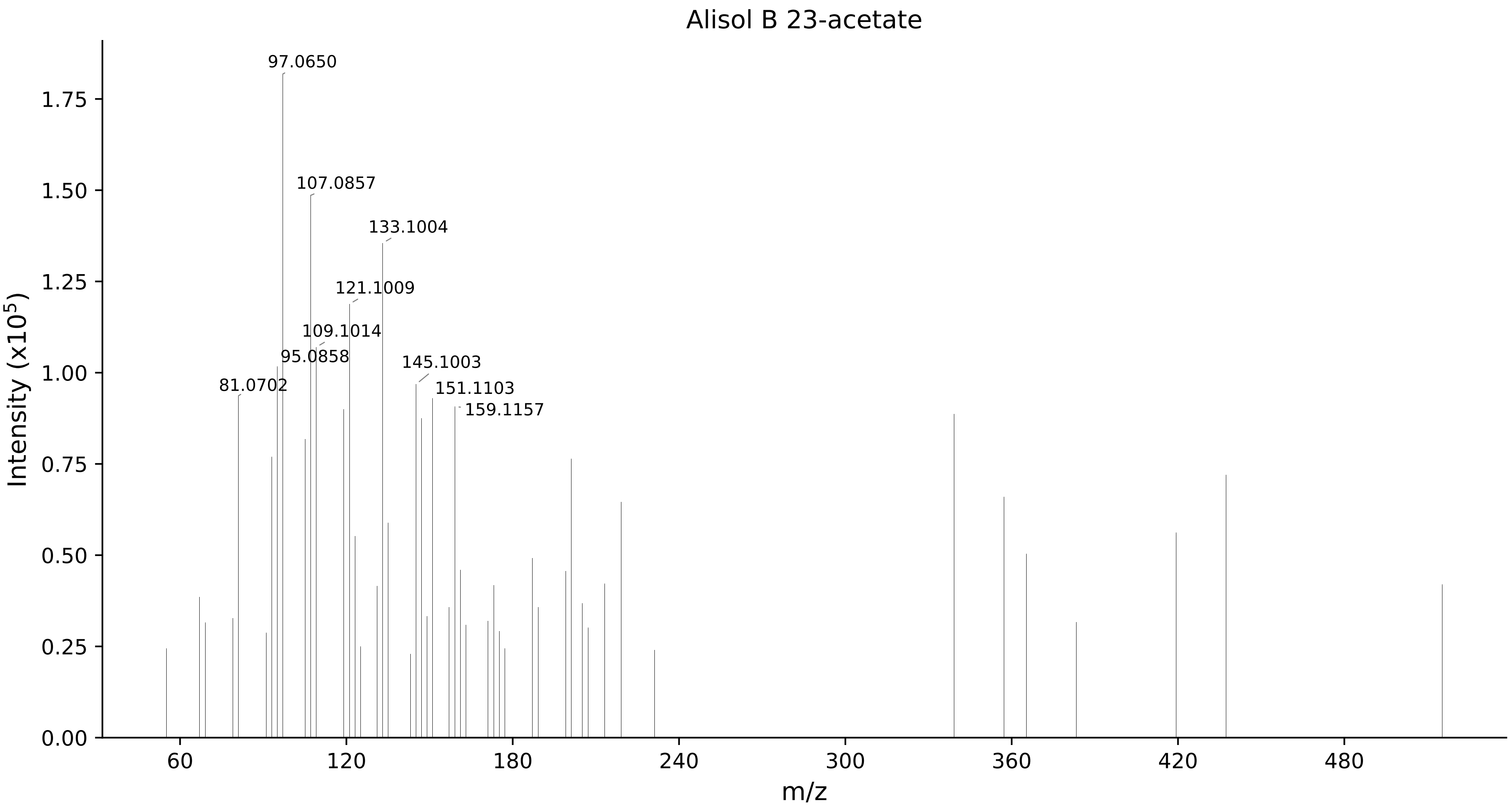

Alisol B 23-acetate
97.0650
107.0857
133.1004
121.1009
109.1014
95.0858
145.1003
81.0702
151.1103
159.1157
Intensity (x10^5)
1.75
1.50
1.25
1.00
0.75
0.50
0.25
0.00
60
120
180
240
300
360
420
480
m/z
