# Supplementary material for: Mafu Yishen Formula ameliorates membranous nephropathy by promotion of regulatory T cell differentiation: a multi-omics study
Source: Chin Med. 2026 Jan 6;21:5. doi: 10.1186/s13020-025-01272-1 (PMC12771757; doi:10.1186/s13020-025-01272-1)
Supplement: Supplementary file 2 — Supplementary material 2. [file 13020_2025_1272_MOESM2_ESM.zip › pos.Piptocarphin_F.pdf]

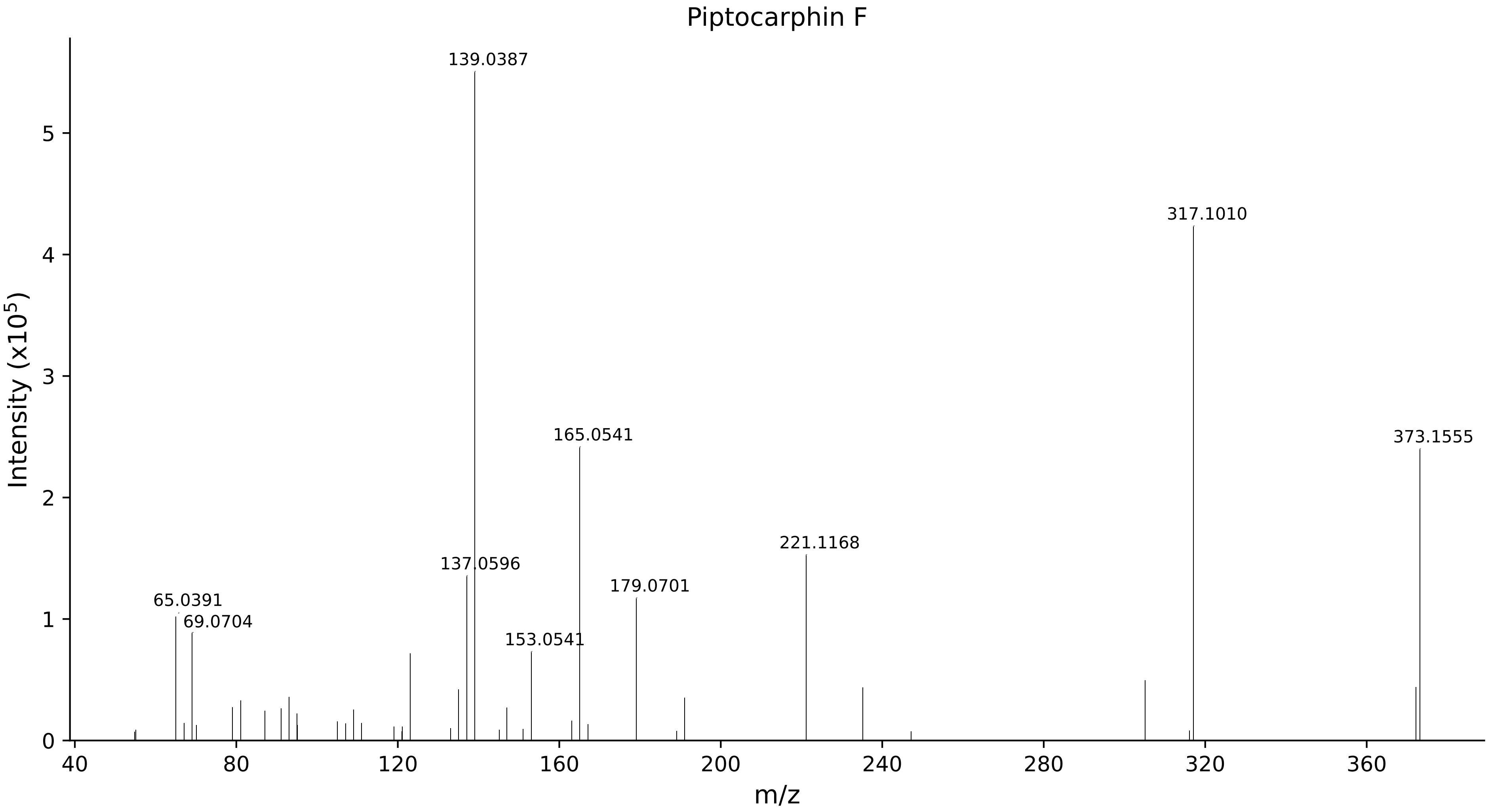

Piptocarphin F
139.0387
317.1010
165.0541
373.1555
221.1168
137.0596
179.0701
65.0391
69.0704
153.0541
Intensity (x10^5)
0
1
2
3
4
5
m/z
40
80
120
160
200
240
280
320
360
