# Supplementary material for: Mafu Yishen Formula ameliorates membranous nephropathy by promotion of regulatory T cell differentiation: a multi-omics study
Source: Chin Med. 2026 Jan 6;21:5. doi: 10.1186/s13020-025-01272-1 (PMC12771757; doi:10.1186/s13020-025-01272-1)
Supplement: Supplementary file 2 — Supplementary material 2. [file 13020_2025_1272_MOESM2_ESM.zip › neg.7-Demethylsuberosin.pdf]

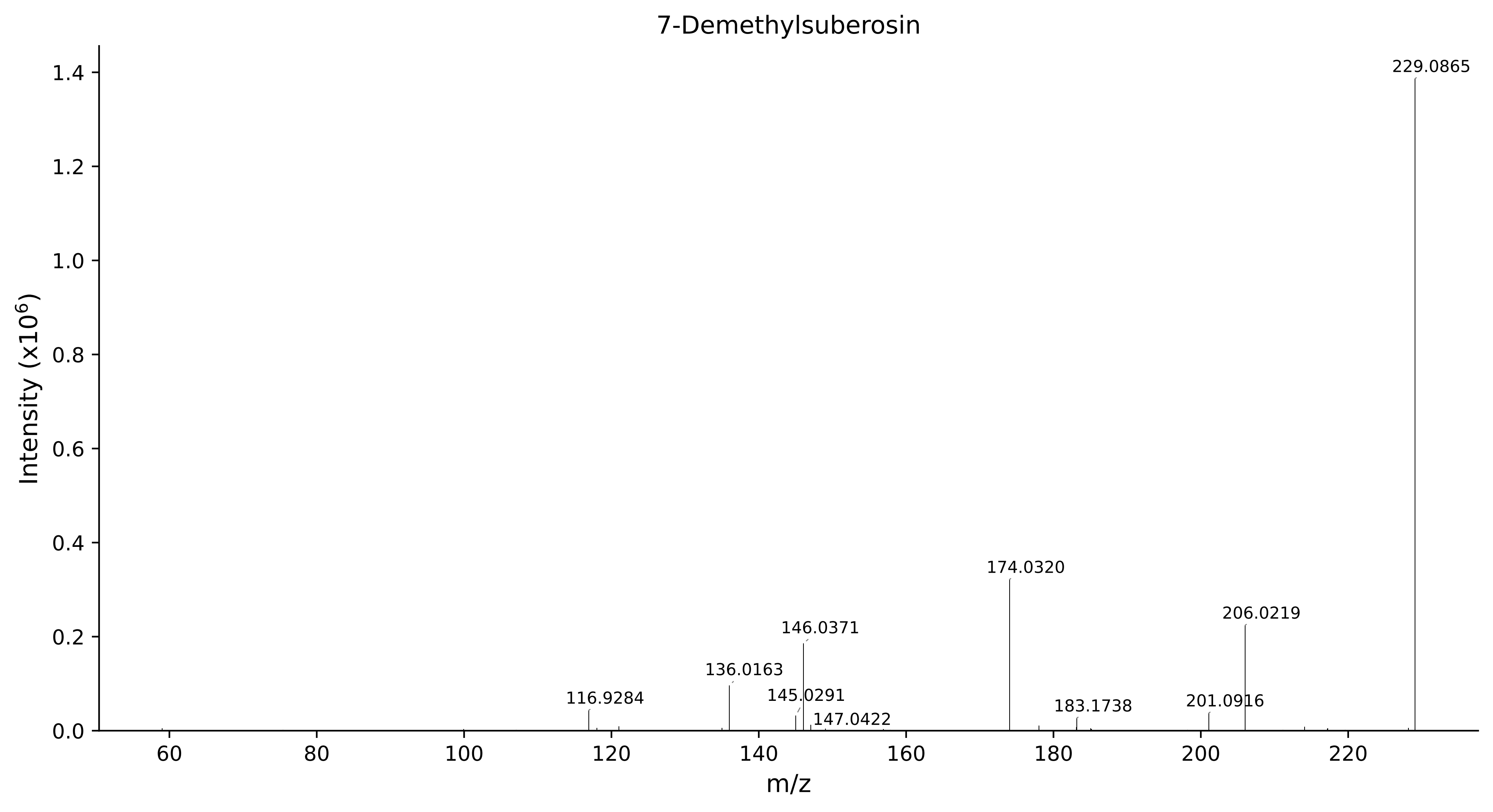
7-Demethylsuberosin
Intensity (x10^6)
m/z
1.4
1.2
1.0
0.8
0.6
0.4
0.2
0.0
60
80
100
120
140
160
180
200
220
229.0865
174.0320
206.0219
146.0371
136.0163
145.0291
116.9284
147.0422
183.1738
201.0916
