# Supplementary material for: Mafu Yishen Formula ameliorates membranous nephropathy by promotion of regulatory T cell differentiation: a multi-omics study
Source: Chin Med. 2026 Jan 6;21:5. doi: 10.1186/s13020-025-01272-1 (PMC12771757; doi:10.1186/s13020-025-01272-1)
Supplement: Supplementary file 2 — Supplementary material 2. [file 13020_2025_1272_MOESM2_ESM.zip › pos.Neolinustatin.pdf]

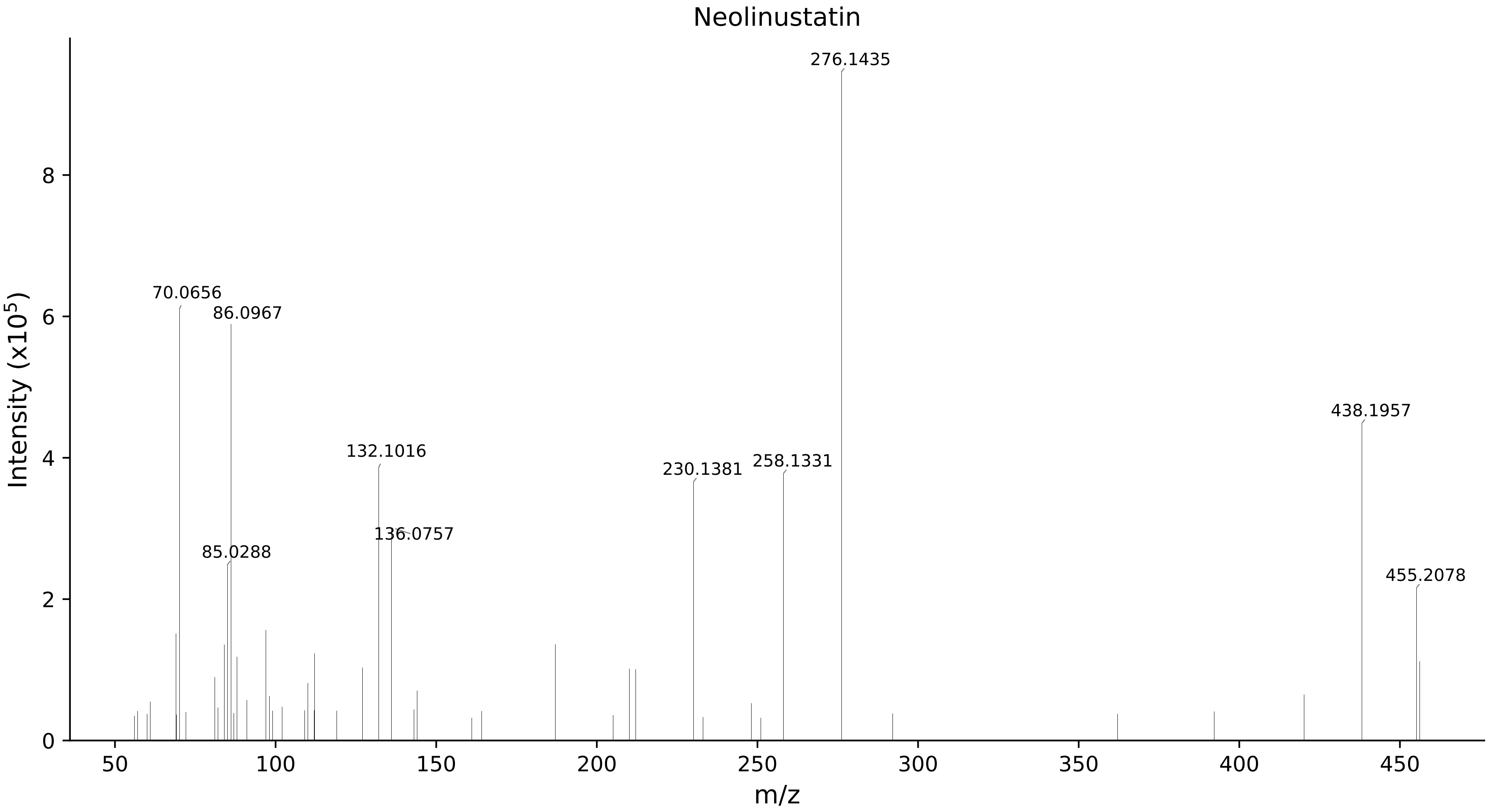

Neolinustatin
276.1435
70.0656
86.0967
438.1957
132.1016
230.1381
258.1331
136.0757
85.0288
455.2078
Intensity (x10^5)
m/z
0
2
4
6
8
50
100
150
200
250
300
350
400
450
