# Supplementary material for: Mafu Yishen Formula ameliorates membranous nephropathy by promotion of regulatory T cell differentiation: a multi-omics study
Source: Chin Med. 2026 Jan 6;21:5. doi: 10.1186/s13020-025-01272-1 (PMC12771757; doi:10.1186/s13020-025-01272-1)
Supplement: Supplementary file 2 — Supplementary material 2. [file 13020_2025_1272_MOESM2_ESM.zip › pos.Quercetin-3-O-glucose-6''-acetate.pdf]

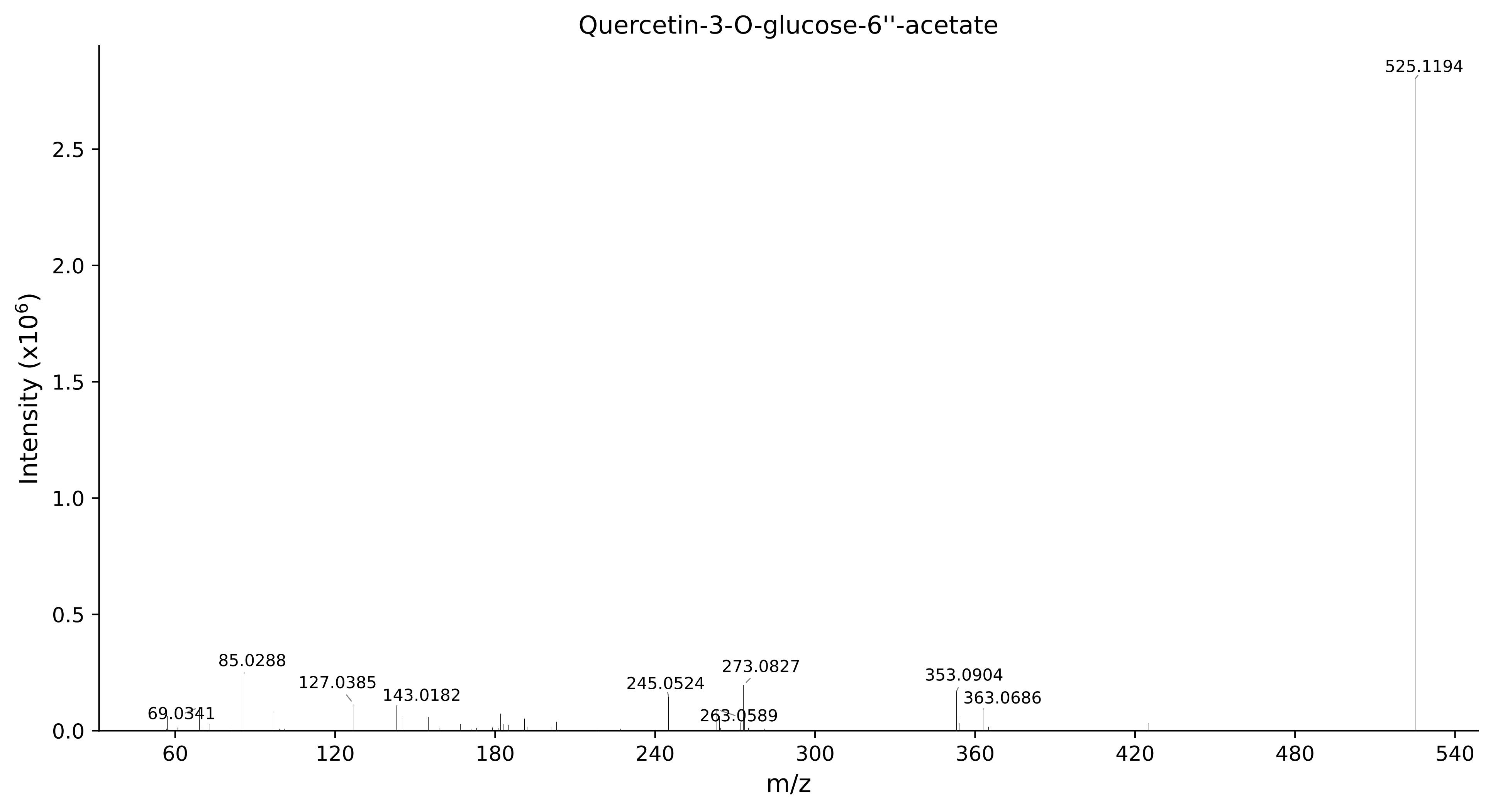

Quercetin-3-O-glucose-6''-acetate
Intensity (x10^6)
m/z
525.1194
85.0288
127.0385
143.0182
69.0341
245.0524
273.0827
263.0589
353.0904
363.0686
0.0
0.5
1.0
1.5
2.0
2.5
60
120
180
240
300
360
420
480
540
