# Supplementary material for: Mafu Yishen Formula ameliorates membranous nephropathy by promotion of regulatory T cell differentiation: a multi-omics study
Source: Chin Med. 2026 Jan 6;21:5. doi: 10.1186/s13020-025-01272-1 (PMC12771757; doi:10.1186/s13020-025-01272-1)
Supplement: Supplementary file 2 — Supplementary material 2. [file 13020_2025_1272_MOESM2_ESM.zip › pos.Kushenol_X.pdf]

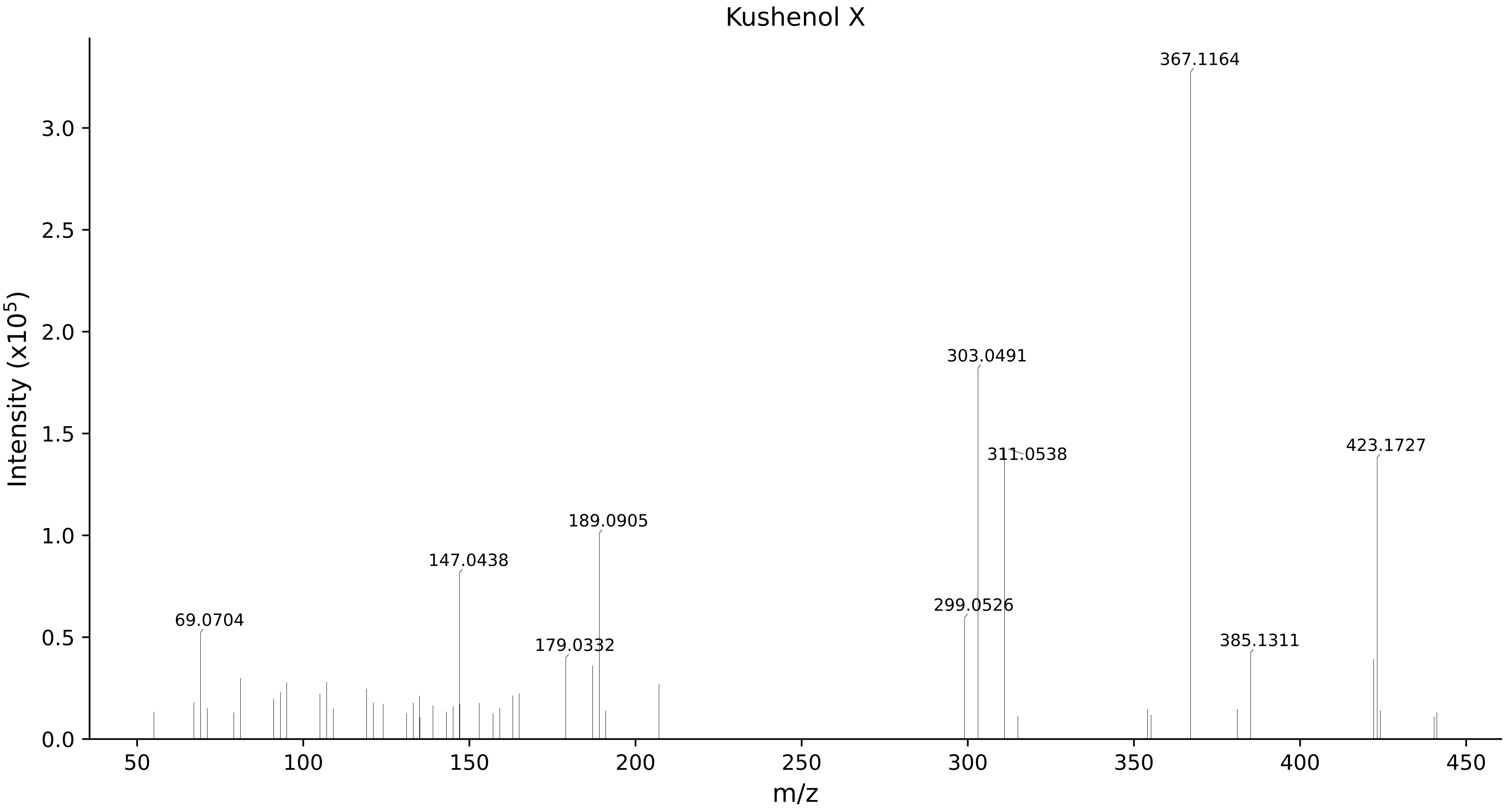

Kushenol X
Intensity (x10^5)
m/z
367.1164
303.0491
311.0538
423.1727
189.0905
147.0438
299.0526
69.0704
179.0332
385.1311
0.0
0.5
1.0
1.5
2.0
2.5
3.0
50
100
150
200
250
300
350
400
450
