# Supplementary material for: Mafu Yishen Formula ameliorates membranous nephropathy by promotion of regulatory T cell differentiation: a multi-omics study
Source: Chin Med. 2026 Jan 6;21:5. doi: 10.1186/s13020-025-01272-1 (PMC12771757; doi:10.1186/s13020-025-01272-1)
Supplement: Supplementary file 2 — Supplementary material 2. [file 13020_2025_1272_MOESM2_ESM.zip › pos.Okaramine_B.pdf]

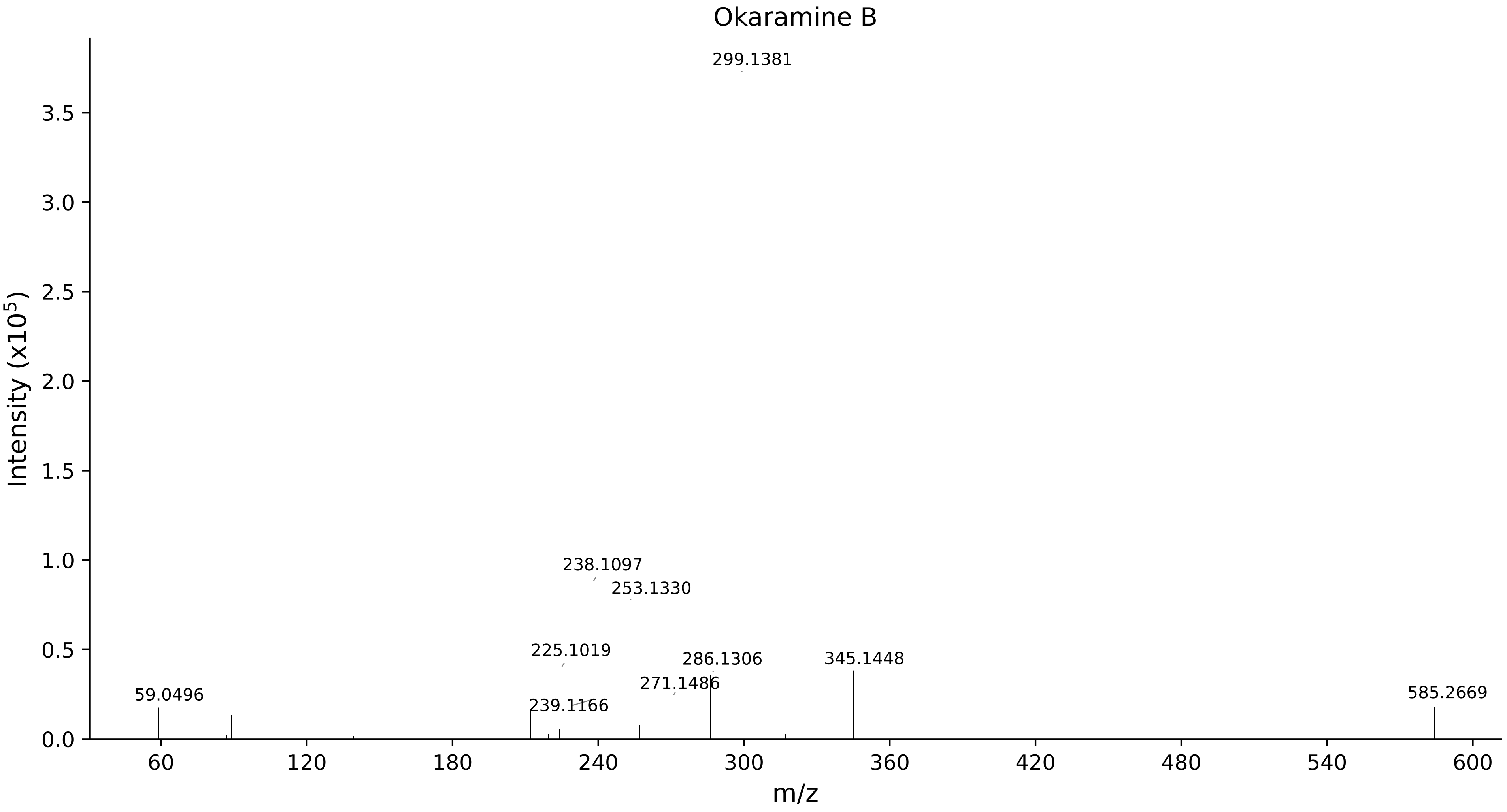
Okaramine B
299.1381
238.1097
253.1330
225.1019
286.1306
345.1448
271.1486
59.0496
239.1166
585.2669
Intensity (x10^5)
0.0
0.5
1.0
1.5
2.0
2.5
3.0
3.5
60
120
180
240
300
360
420
480
540
600
m/z
