# Supplementary material for: Mafu Yishen Formula ameliorates membranous nephropathy by promotion of regulatory T cell differentiation: a multi-omics study
Source: Chin Med. 2026 Jan 6;21:5. doi: 10.1186/s13020-025-01272-1 (PMC12771757; doi:10.1186/s13020-025-01272-1)
Supplement: Supplementary file 2 — Supplementary material 2. [file 13020_2025_1272_MOESM2_ESM.zip › pos.Coagulin_L.pdf]

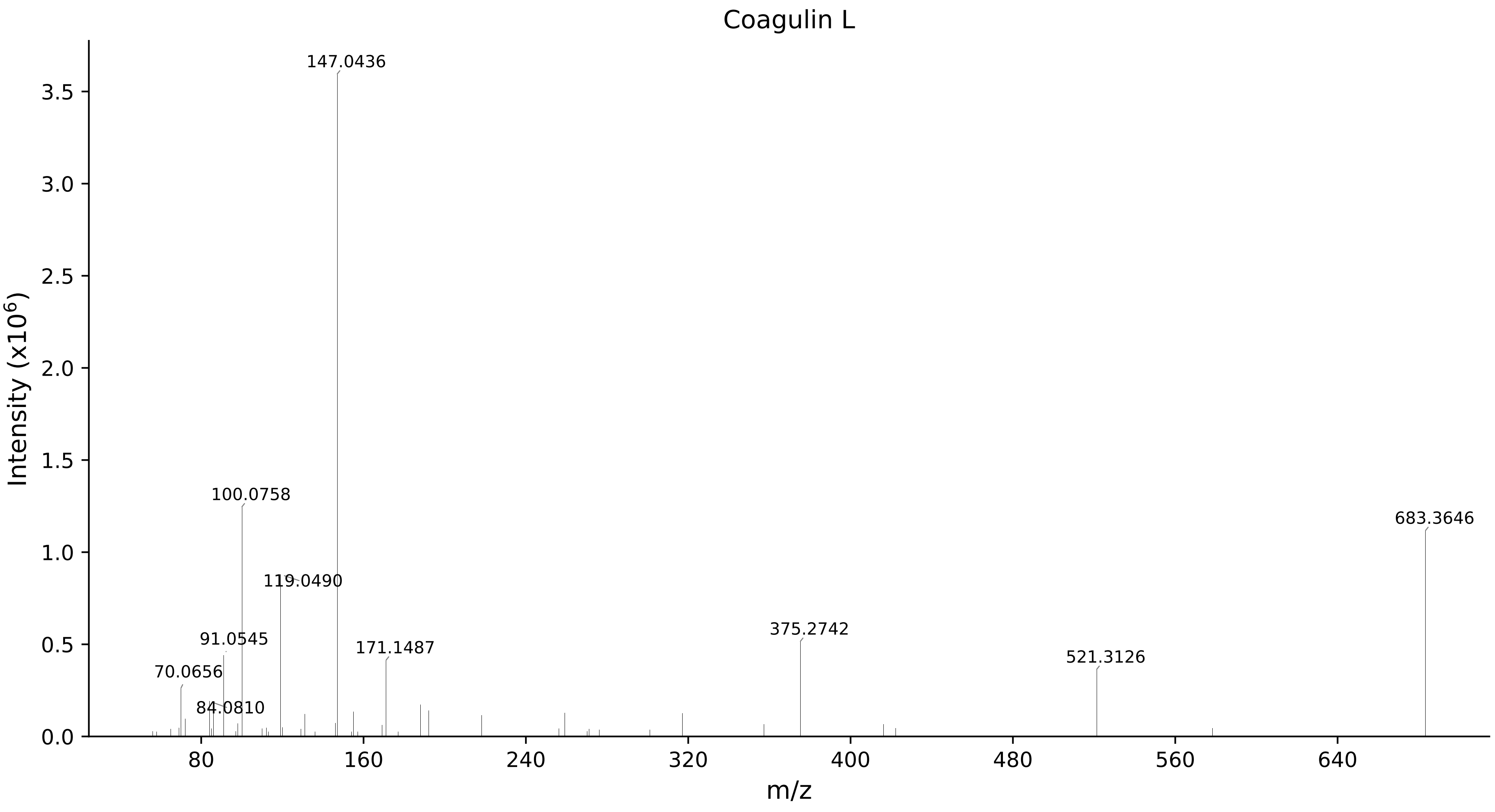
Coagulin L
Intensity (x10^6)
0.0
0.5
1.0
1.5
2.0
2.5
3.0
3.5
m/z
80
160
240
320
400
480
560
640
147.0436
100.0758
683.3646
119.0490
375.2742
91.0545
171.1487
521.3126
70.0656
84.0810
