# Supplementary material for: Mafu Yishen Formula ameliorates membranous nephropathy by promotion of regulatory T cell differentiation: a multi-omics study
Source: Chin Med. 2026 Jan 6;21:5. doi: 10.1186/s13020-025-01272-1 (PMC12771757; doi:10.1186/s13020-025-01272-1)
Supplement: Supplementary file 2 — Supplementary material 2. [file 13020_2025_1272_MOESM2_ESM.zip › neg.1,2-Didehydrotanshinone_IIA.pdf]

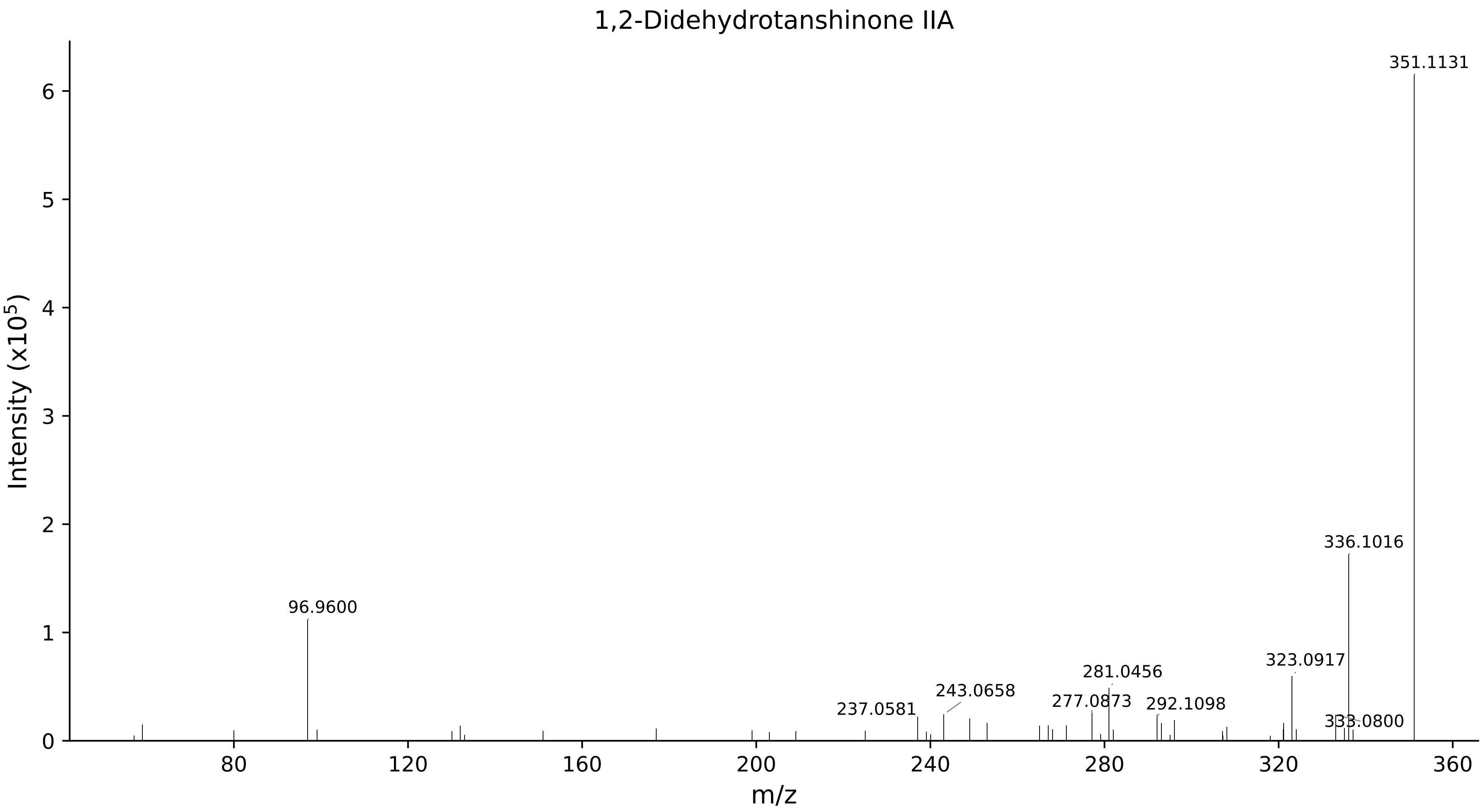
1,2-Didehydrotanshinone IIA
Intensity (x10^5)
m/z
351.1131
336.1016
96.9600
323.0917
281.0456
243.0658
237.0581
277.0873
292.1098
333.0800
0
1
2
3
4
5
6
80
120
160
200
240
280
320
360
