# Supplementary material for: Mafu Yishen Formula ameliorates membranous nephropathy by promotion of regulatory T cell differentiation: a multi-omics study
Source: Chin Med. 2026 Jan 6;21:5. doi: 10.1186/s13020-025-01272-1 (PMC12771757; doi:10.1186/s13020-025-01272-1)
Supplement: Supplementary file 2 — Supplementary material 2. [file 13020_2025_1272_MOESM2_ESM.zip › neg.Engeletin.pdf]

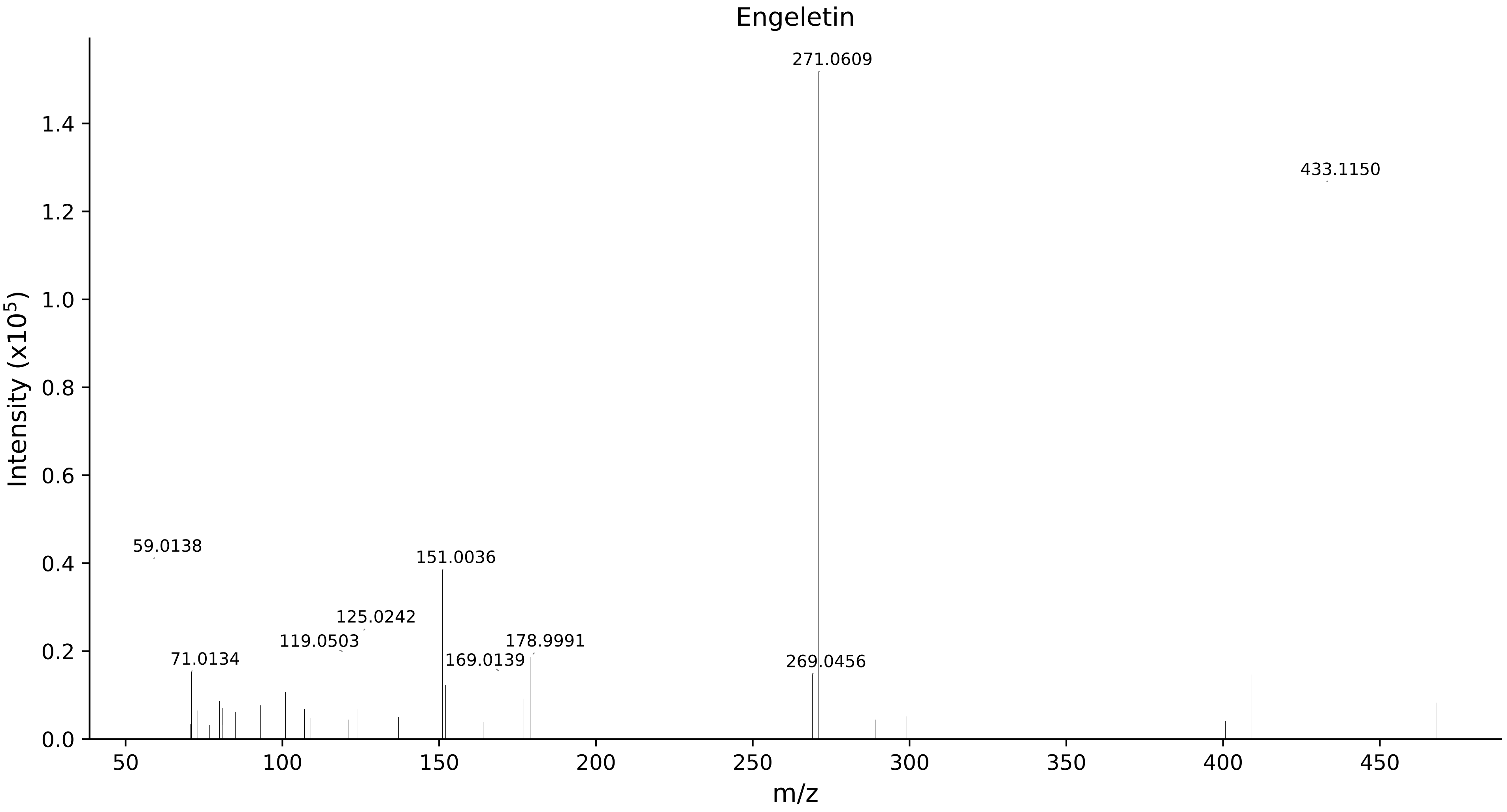

Engeletin
271.0609
433.1150
59.0138
151.0036
125.0242
119.0503
178.9991
71.0134
169.0139
269.0456
Intensity (x10^5)
0.0
0.2
0.4
0.6
0.8
1.0
1.2
1.4
50
100
150
200
250
300
350
400
450
m/z
