# Supplementary material for: Mafu Yishen Formula ameliorates membranous nephropathy by promotion of regulatory T cell differentiation: a multi-omics study
Source: Chin Med. 2026 Jan 6;21:5. doi: 10.1186/s13020-025-01272-1 (PMC12771757; doi:10.1186/s13020-025-01272-1)
Supplement: Supplementary file 2 — Supplementary material 2. [file 13020_2025_1272_MOESM2_ESM.zip › neg.4'-Demethylpodophyllotoxin.pdf]

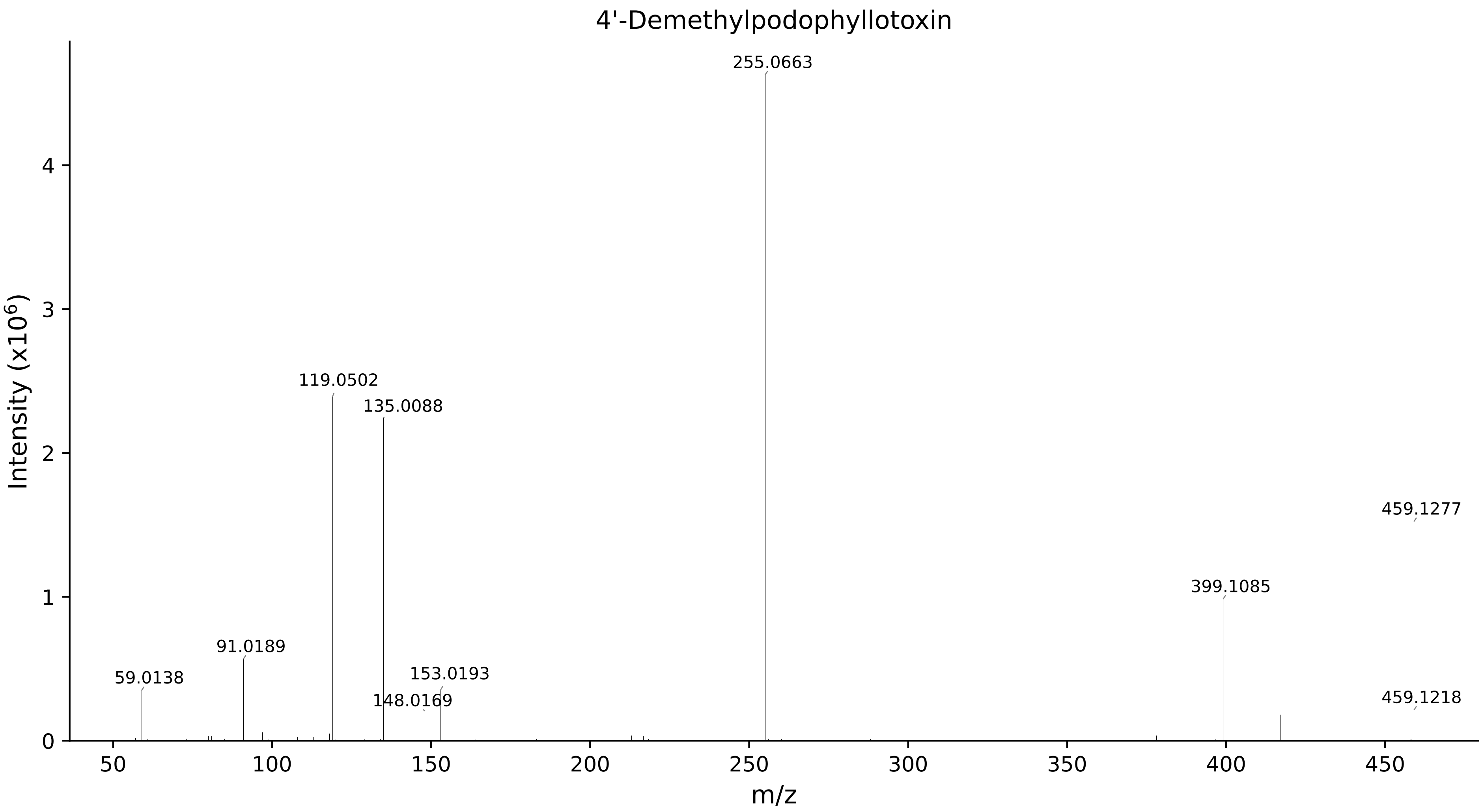

4'-Demethylpodophyllotoxin
255.0663
119.0502
135.0088
459.1277
399.1085
91.0189
59.0138
153.0193
148.0169
459.1218
Intensity (x10^6)
0
1
2
3
4
50
100
150
200
250
300
350
400
450
m/z
