# Supplementary material for: Mafu Yishen Formula ameliorates membranous nephropathy by promotion of regulatory T cell differentiation: a multi-omics study
Source: Chin Med. 2026 Jan 6;21:5. doi: 10.1186/s13020-025-01272-1 (PMC12771757; doi:10.1186/s13020-025-01272-1)
Supplement: Supplementary file 2 — Supplementary material 2. [file 13020_2025_1272_MOESM2_ESM.zip › pos.Ecgonine.pdf]

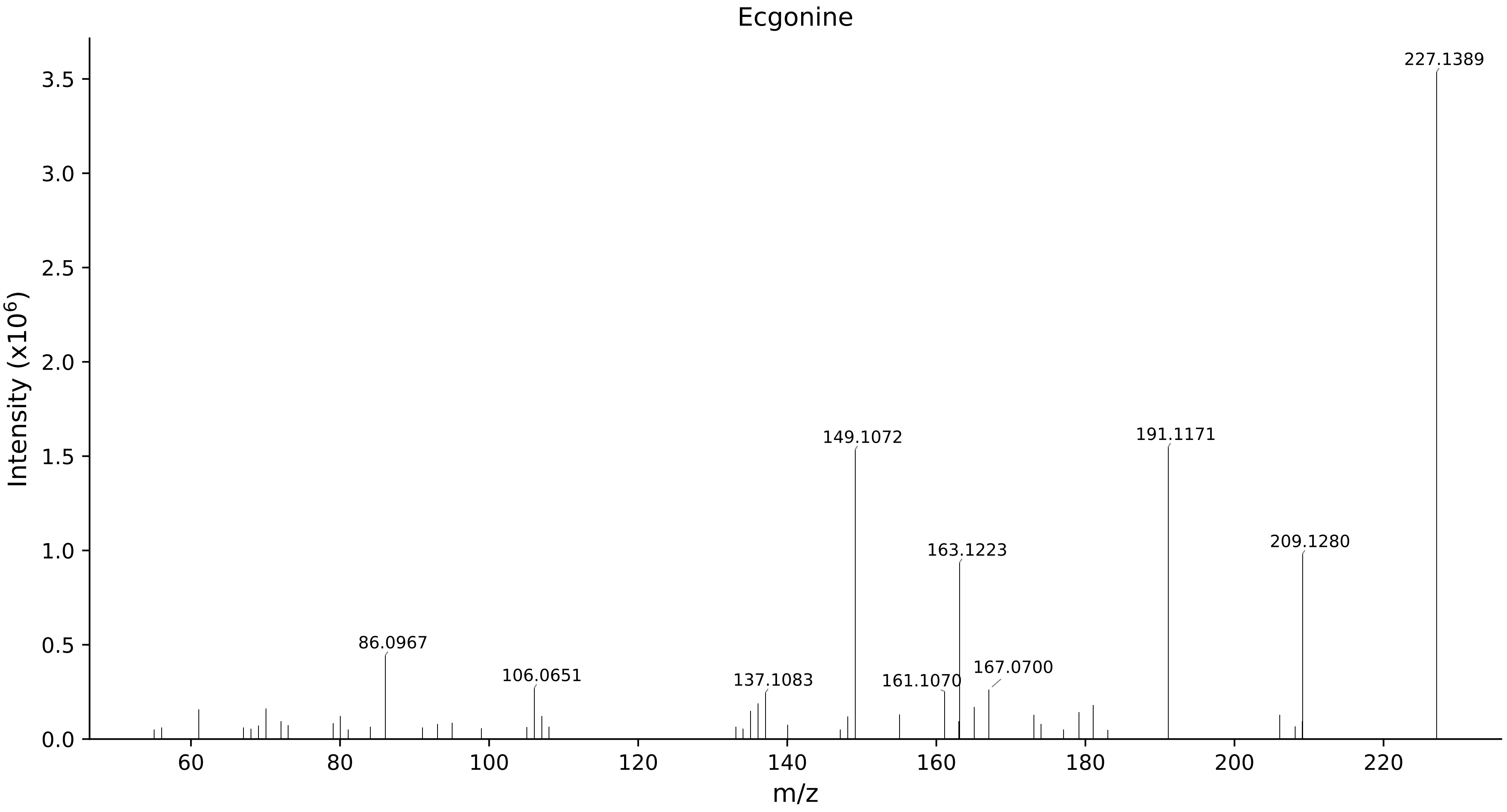
Ecgonine
227.1389
149.1072
191.1171
163.1223
209.1280
86.0967
106.0651
137.1083
161.1070
167.0700
Intensity (x10^6)
0.0
0.5
1.0
1.5
2.0
2.5
3.0
3.5
60
80
100
120
140
160
180
200
220
m/z
