# Supplementary material for: Mafu Yishen Formula ameliorates membranous nephropathy by promotion of regulatory T cell differentiation: a multi-omics study
Source: Chin Med. 2026 Jan 6;21:5. doi: 10.1186/s13020-025-01272-1 (PMC12771757; doi:10.1186/s13020-025-01272-1)
Supplement: Supplementary file 2 — Supplementary material 2. [file 13020_2025_1272_MOESM2_ESM.zip › pos.Calealactone_C.pdf]

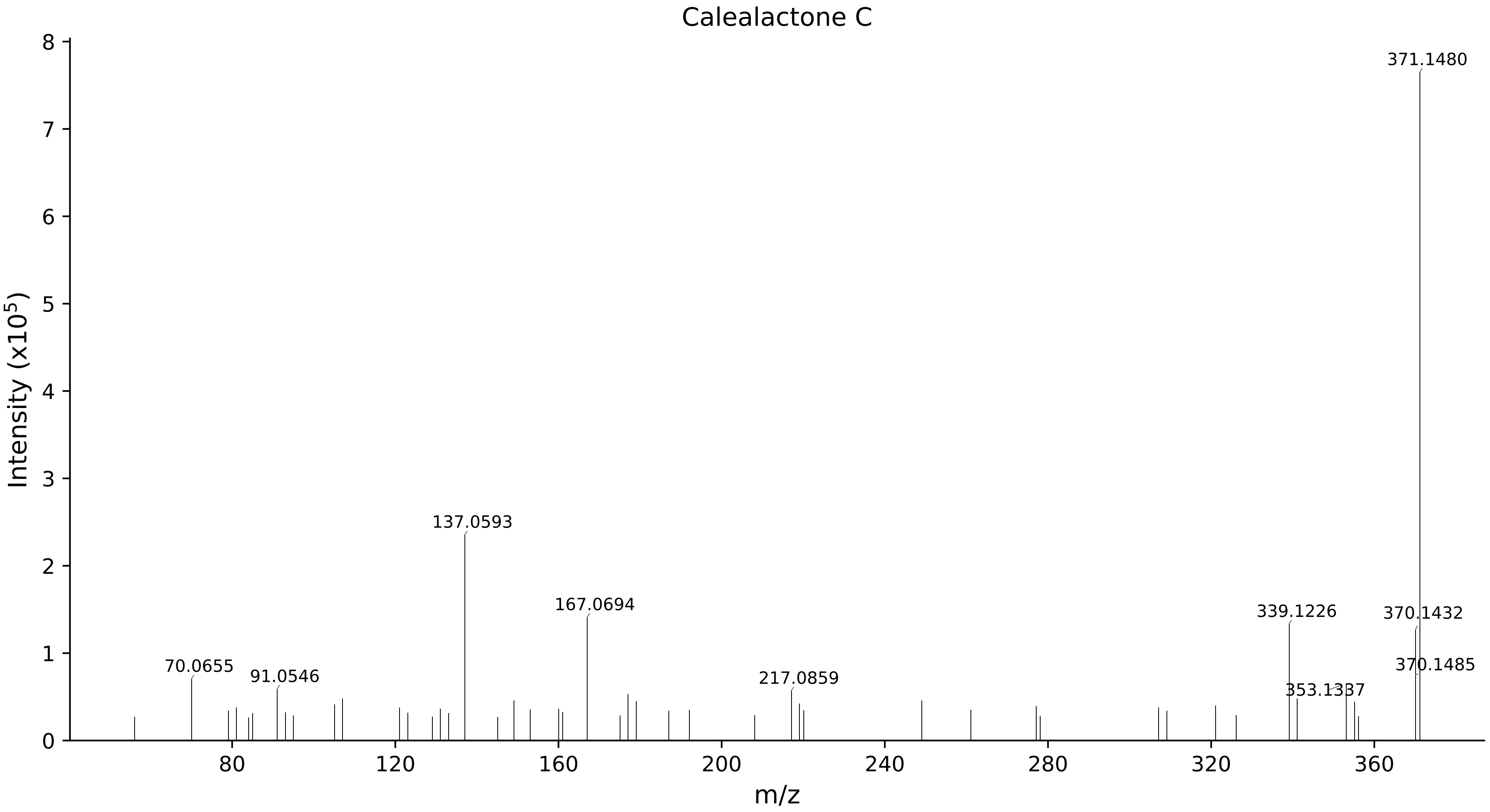
Calealactone C
Intensity (x10^5)
m/z
371.1480
137.0593
167.0694
339.1226
370.1432
370.1485
70.0655
91.0546
217.0859
353.1337
0
1
2
3
4
5
6
7
8
80
120
160
200
240
280
320
360
