# Supplementary material for: Mafu Yishen Formula ameliorates membranous nephropathy by promotion of regulatory T cell differentiation: a multi-omics study
Source: Chin Med. 2026 Jan 6;21:5. doi: 10.1186/s13020-025-01272-1 (PMC12771757; doi:10.1186/s13020-025-01272-1)
Supplement: Supplementary file 2 — Supplementary material 2. [file 13020_2025_1272_MOESM2_ESM.zip › neg.Denudaquinol.pdf]

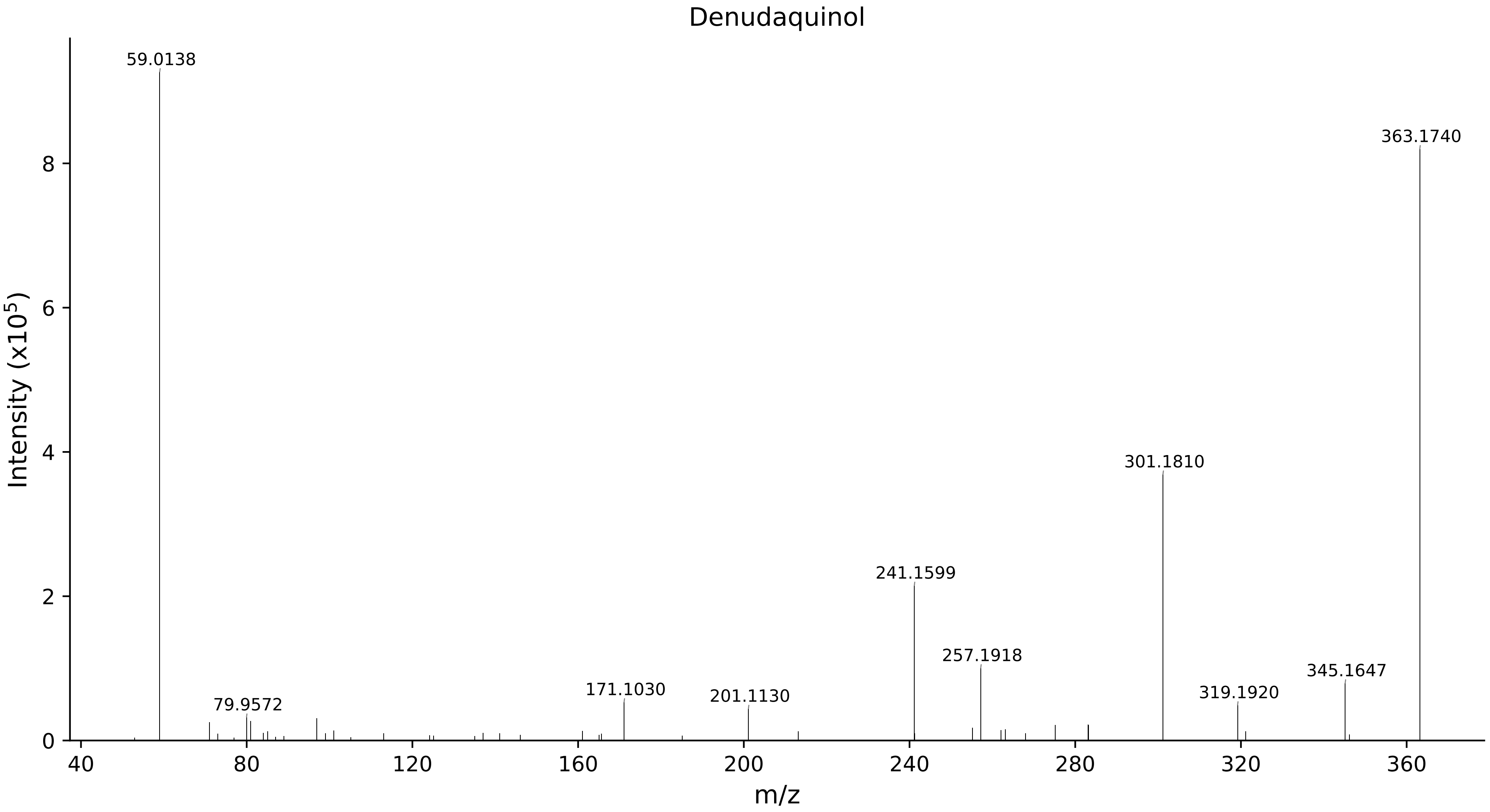
Denudaquinol
59.0138
363.1740
301.1810
241.1599
257.1918
345.1647
319.1920
171.1030
201.1130
79.9572
Intensity (x10^5)
m/z
0
2
4
6
8
40
80
120
160
200
240
280
320
360
