# Supplementary material for: Mafu Yishen Formula ameliorates membranous nephropathy by promotion of regulatory T cell differentiation: a multi-omics study
Source: Chin Med. 2026 Jan 6;21:5. doi: 10.1186/s13020-025-01272-1 (PMC12771757; doi:10.1186/s13020-025-01272-1)
Supplement: Supplementary file 2 — Supplementary material 2. [file 13020_2025_1272_MOESM2_ESM.zip › neg.1,3-Dicaffeoylquinic_acid.pdf]

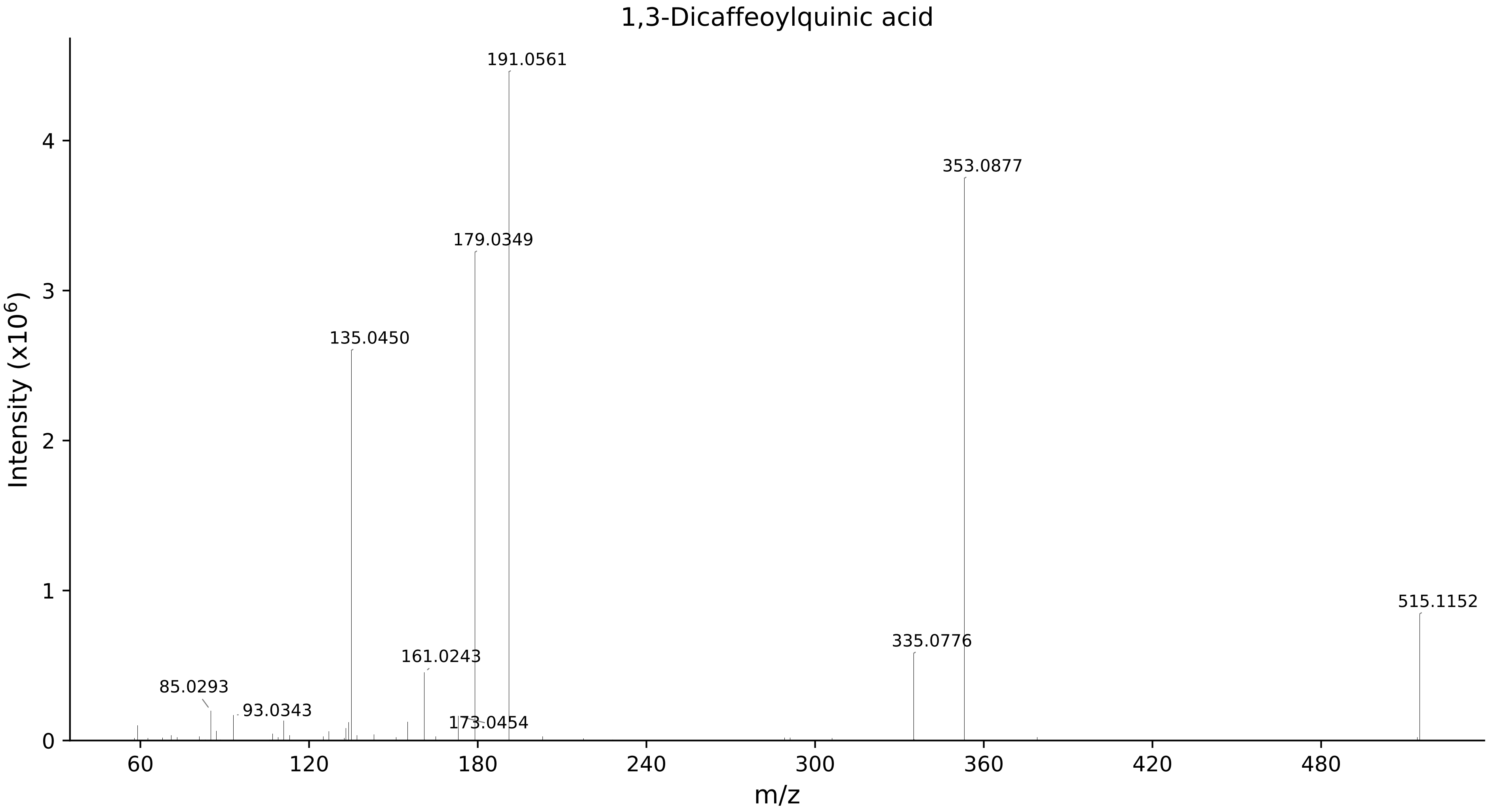

1,3-Dicaffeoylquinic acid
Intensity (x10^6)
m/z
0
1
2
3
4
60
120
180
240
300
360
420
480
191.0561
353.0877
179.0349
135.0450
515.1152
335.0776
161.0243
85.0293
93.0343
173.0454
