# Supplementary material for: Mafu Yishen Formula ameliorates membranous nephropathy by promotion of regulatory T cell differentiation: a multi-omics study
Source: Chin Med. 2026 Jan 6;21:5. doi: 10.1186/s13020-025-01272-1 (PMC12771757; doi:10.1186/s13020-025-01272-1)
Supplement: Supplementary file 2 — Supplementary material 2. [file 13020_2025_1272_MOESM2_ESM.zip › pos.Narchinol_B.pdf]

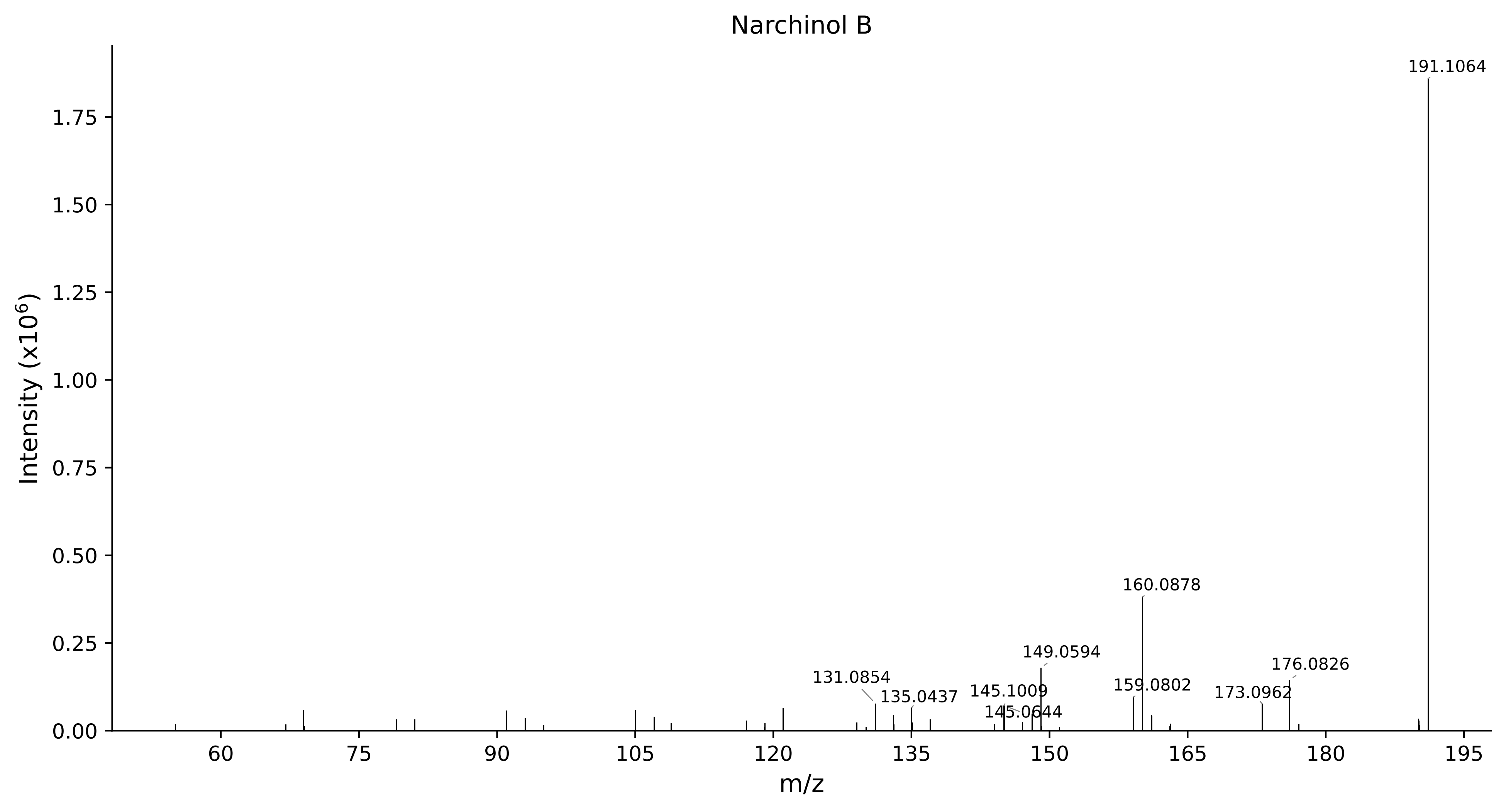
Narchinol B
Intensity (x10^6)
0.00
0.25
0.50
0.75
1.00
1.25
1.50
1.75
m/z
60
75
90
105
120
135
150
165
180
195
191.1064
160.0878
149.0594
176.0826
131.0854
159.0802
145.1009
173.0962
135.0437
145.0644
