# Supplementary material for: Mafu Yishen Formula ameliorates membranous nephropathy by promotion of regulatory T cell differentiation: a multi-omics study
Source: Chin Med. 2026 Jan 6;21:5. doi: 10.1186/s13020-025-01272-1 (PMC12771757; doi:10.1186/s13020-025-01272-1)
Supplement: Supplementary file 2 — Supplementary material 2. [file 13020_2025_1272_MOESM2_ESM.zip › pos.Taxifolin_6-C-glucoside.pdf]

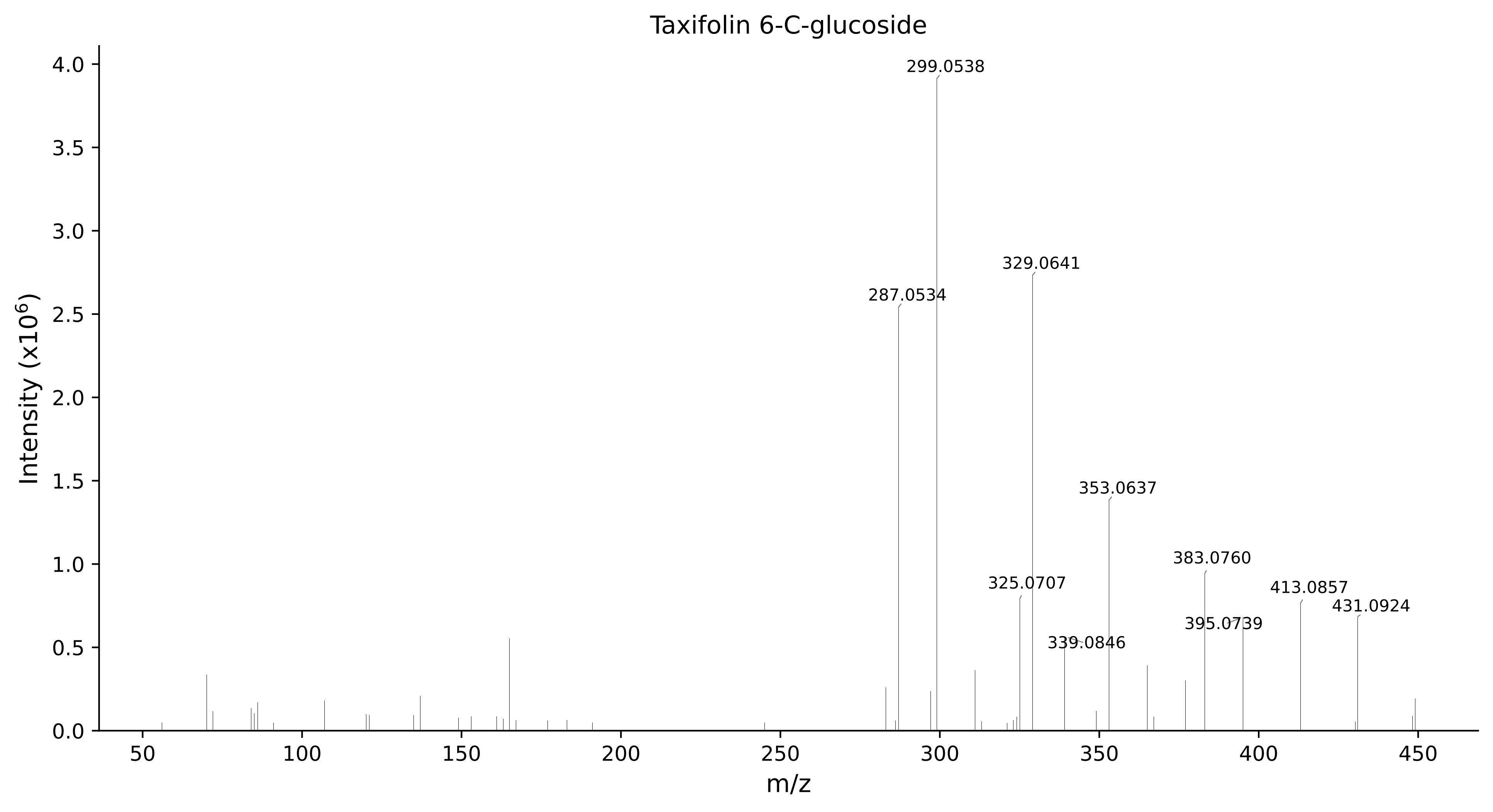

Taxifolin 6-C-glucoside
Intensity (x10^6)
m/z
299.0538
329.0641
287.0534
353.0637
383.0760
325.0707
413.0857
431.0924
395.0739
339.0846
