# Supplementary material for: Mafu Yishen Formula ameliorates membranous nephropathy by promotion of regulatory T cell differentiation: a multi-omics study
Source: Chin Med. 2026 Jan 6;21:5. doi: 10.1186/s13020-025-01272-1 (PMC12771757; doi:10.1186/s13020-025-01272-1)
Supplement: Supplementary file 2 — Supplementary material 2. [file 13020_2025_1272_MOESM2_ESM.zip › pos.Gnidilatidin.pdf]

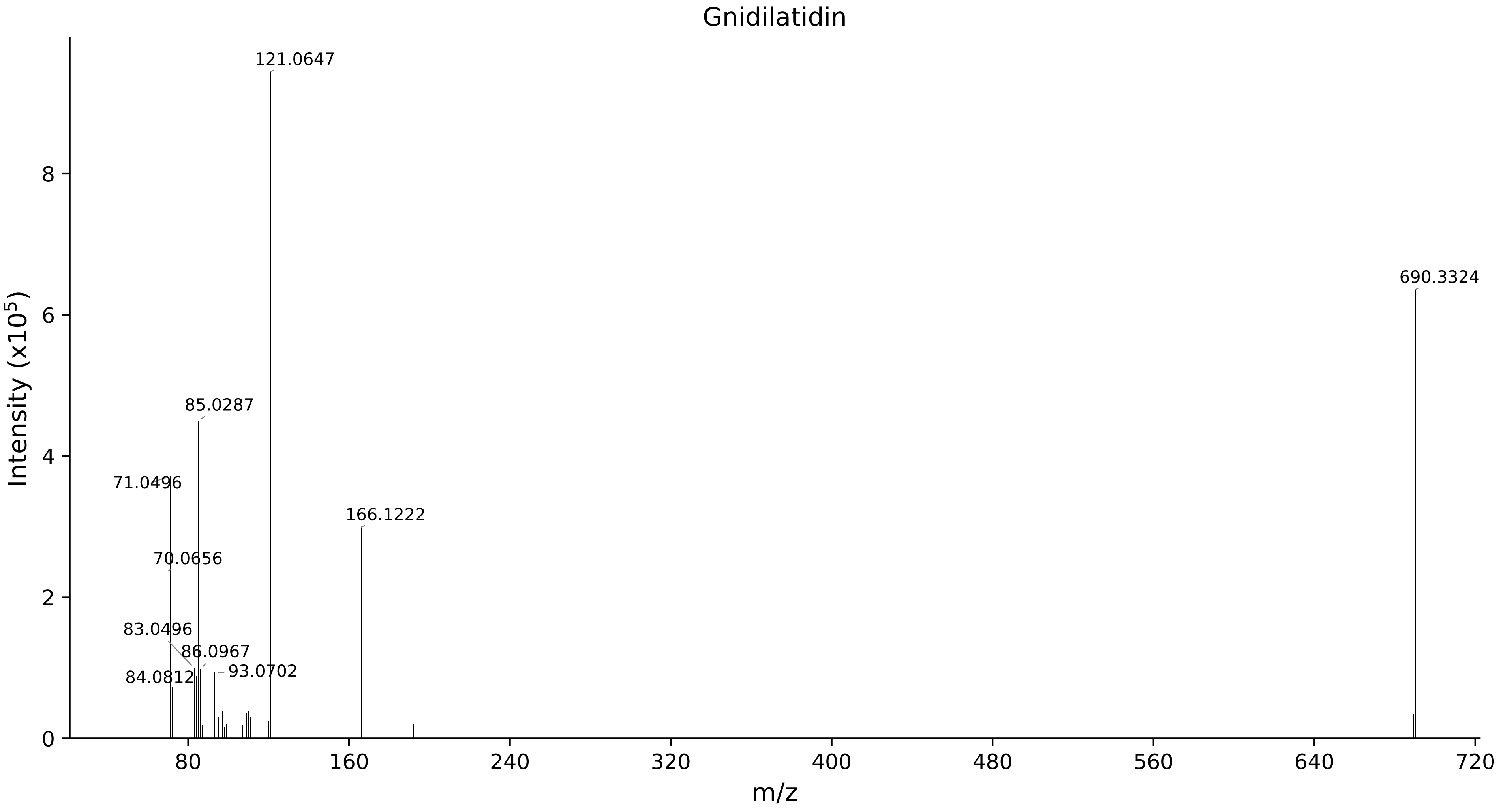
Gnidilatidin
121.0647
690.3324
85.0287
71.0496
166.1222
70.0656
83.0496
86.0967
93.0702
84.0812
Intensity (x10^5)
8
6
4
2
0
80
160
240
320
400
480
560
640
720
m/z
