# Supplementary material for: Mafu Yishen Formula ameliorates membranous nephropathy by promotion of regulatory T cell differentiation: a multi-omics study
Source: Chin Med. 2026 Jan 6;21:5. doi: 10.1186/s13020-025-01272-1 (PMC12771757; doi:10.1186/s13020-025-01272-1)
Supplement: Supplementary file 2 — Supplementary material 2. [file 13020_2025_1272_MOESM2_ESM.zip › pos.13-Hydroxygermacrone.pdf]

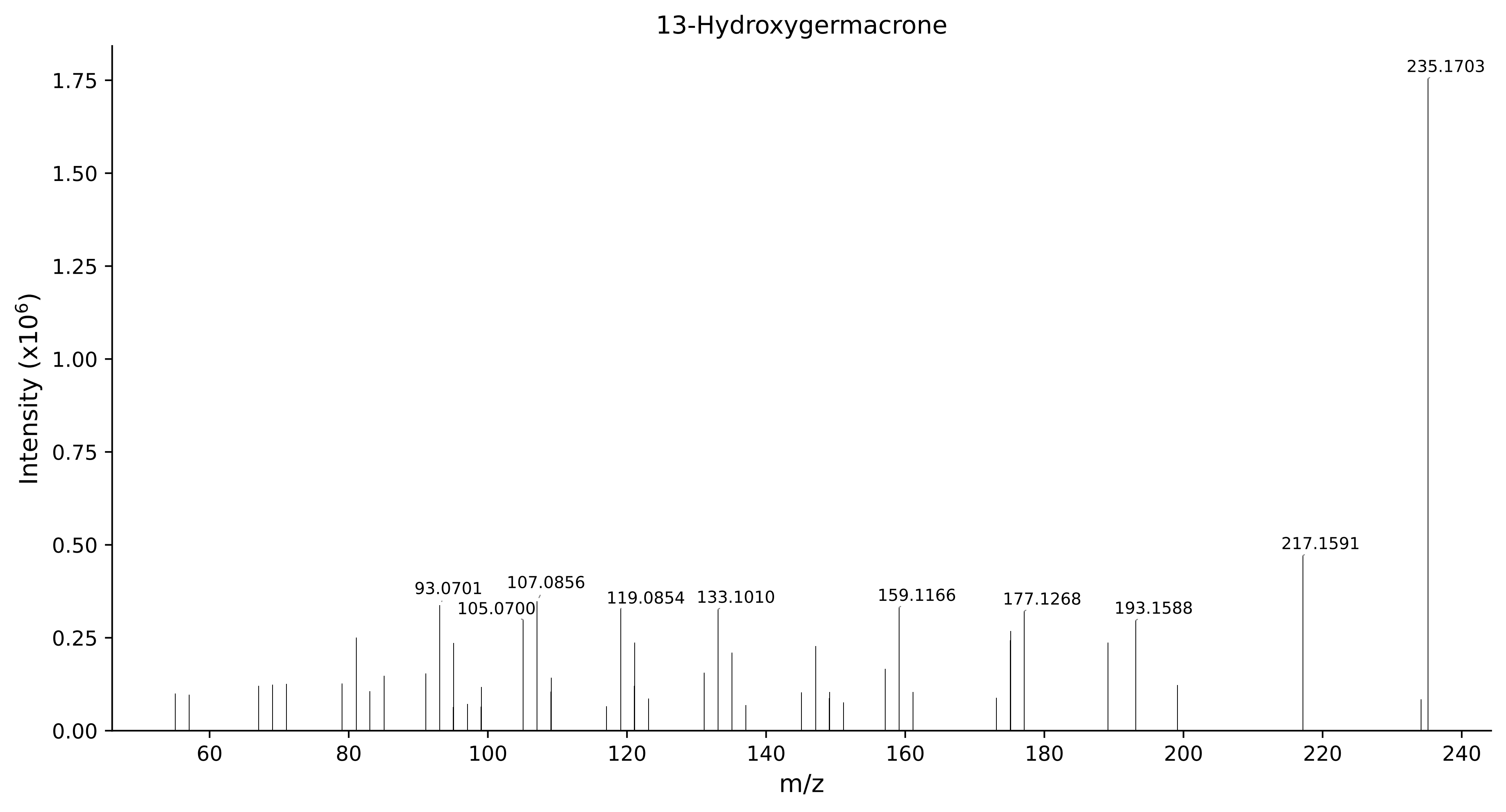
13-Hydroxygermacrone
Intensity (x10^6)
m/z
0.00
0.25
0.50
0.75
1.00
1.25
1.50
1.75
60
80
100
120
140
160
180
200
220
240
235.1703
217.1591
93.0701
107.0856
105.0700
119.0854
133.1010
159.1166
177.1268
193.1588
