# Supplementary material for: Mafu Yishen Formula ameliorates membranous nephropathy by promotion of regulatory T cell differentiation: a multi-omics study
Source: Chin Med. 2026 Jan 6;21:5. doi: 10.1186/s13020-025-01272-1 (PMC12771757; doi:10.1186/s13020-025-01272-1)
Supplement: Supplementary file 2 — Supplementary material 2. [file 13020_2025_1272_MOESM2_ESM.zip › pos.7-Acetylscorpioidine.pdf]

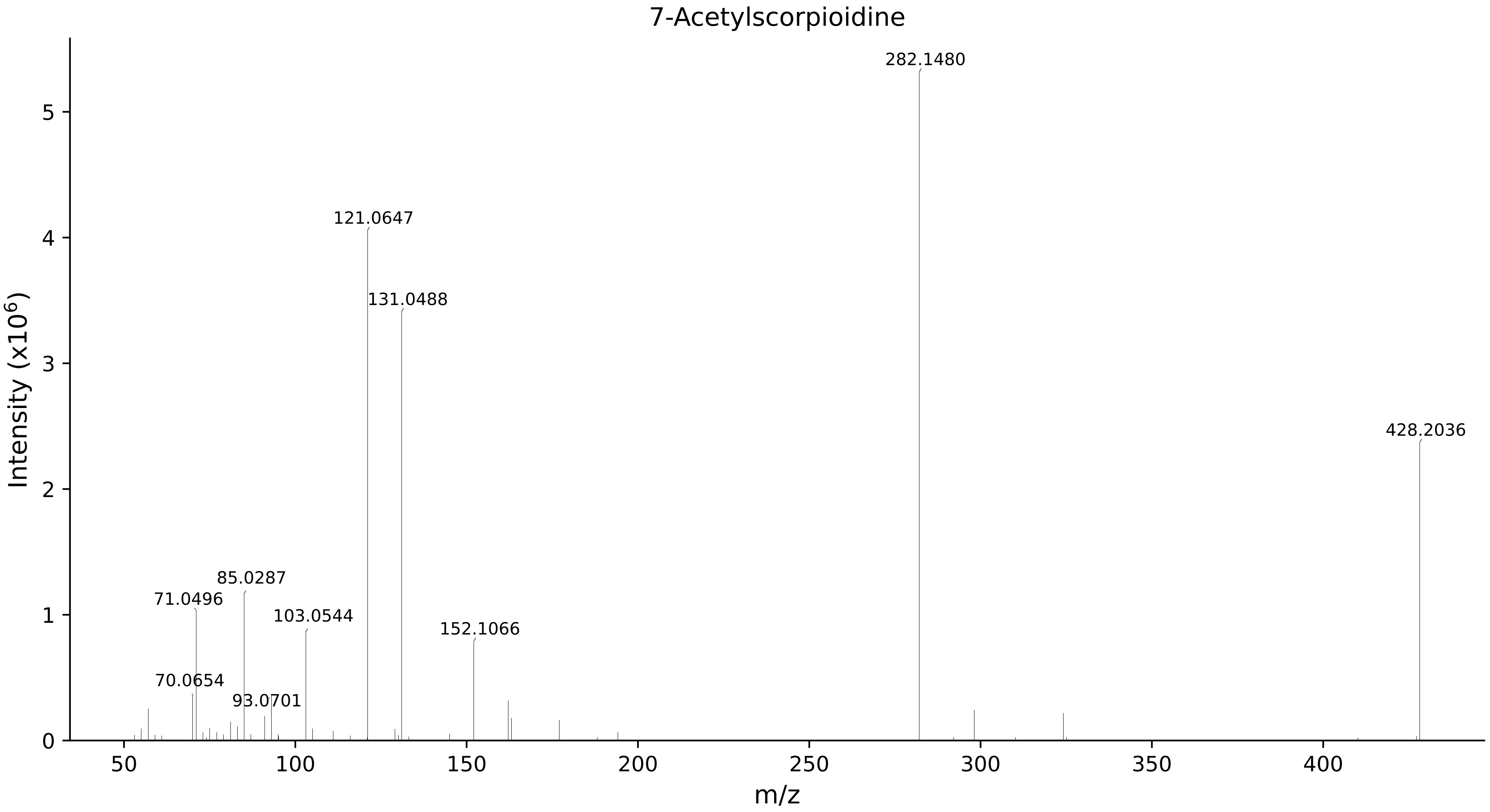

7-Acetylscorpioidine
282.1480
121.0647
131.0488
428.2036
85.0287
71.0496
103.0544
152.1066
70.0654
93.0701
Intensity (x10^6)
0
1
2
3
4
5
50
100
150
200
250
300
350
400
m/z
